# Predicting Drug Loading Capacity for PLA-Amorphous Drug System Using Hansen Solubility Parameters

**DOI:** 10.3390/pharmaceutics18030396

**Published:** 2026-03-23

**Authors:** Artūrs Paulausks, Artjoms Iljičevs, Jurga Bernatoniene, Līga Pētersone, Konstantīns Logviss

**Affiliations:** 1 Laboratory of Finished Dosage Forms, Faculty of Pharmacy, Rīga Stradiņš University, LV-1007 Riga, Latvia; 058103@rsu.edu.lv (A.I.); konstantins.logviss@rsu.lv (K.L.); 2 Baltic Biomaterials Centre of Excellence, Headquarters at Riga Technical University, LV-1048 Riga, Latvia; 3 Institute of Pharmaceutical Technologies, Lithuanian University of Health Sciences, LT-50161 Kaunas, Lithuania; jurga.bernatoniene@lsmu.lt; 4 Department of Drug Technology and Social Pharmacy, Faculty of Pharmacy, Lithuanian University of Health Sciences, LT-50161 Kaunas, Lithuania; 5 Lead Researcher Group, Faculty of Pharmacy, Rīga Stradiņš University, LV-1007 Riga, Latvia; liga.petersone@rsu.lv

**Keywords:** amorphous solid dispersions, solvent casting, solubility parameters, vacuum compression moulding, drug loading, XRD, HPLC, PLA

## Abstract

**Objective:** In this work, we look at Hansen solubility parameters (HSPs) to predict drug miscibility with polymers, in order to create a saturated amorphous drug phase. **Methods:** We used the Yamamoto molecular break (Y-MB) group contribution method (GCM) and solvent experiments to establish HSPs for PLA and 12 model drugs. Drug-loaded samples were made using solvent casting (SC) and vacuum compression moulding (VCM) in incremental drug concentrations until a saturated amorphous drug load was achieved. The amorphous drug phase was confirmed by X-ray diffraction after 24 h. These amorphous samples were further analysed by HPLC to confirm drug concentration. These drug concentrations were expressed as volume concentration in PLA, and they correlate with linearised HSP distance between drug and polymer. **Results:** This gives a statistically significant linear correlation between drug concentration and HSPs with R^2^ values ranging from 0.85 to 0.93 for SC and VCM methods. **Conclusions:** This work entails a possible concept for novel application of HSPs to predict miscible drug–polymer pairs and to estimate amorphous saturation concentration.

## 1. Introduction

Many active pharmaceutical ingredients (APIs) are formulated into polymer materials to be used as implantable drug delivery vehicles, nanoparticle-based drug delivery systems, or as amorphous solid dispersions (ASD). Since many new APIs are poorly water soluble, the formation of the amorphous drug phase is emphasised to increase their solubility [1,2]. Drug molecules in an amorphous state are trapped in a high-energy non-crystalline form, which require less energy to dissolve [3]. In combination with soluble polymers, the API dissolution rate is also partially dependent on polymer dissolution. The formulation of API–polymer dosage forms is often a challenging and time and resource consuming process, since there are a wide variety of APIs, polymers, possible amounts of API to be loaded in each polymer, and different drug loading methods.

To quickly find a possible good match between a given API and a polymer carrier, several computational methods are used, one of them being the Hansen solubility parameters (HSPs) [3]. The HSPs were first proposed as semi-empirical method to find a solvent for polymer materials, which involves establishing three solubility parameters—*σD* (dispersion force interactions), σH (hydrogen-bonding interactions) and *σP* (polar force interactions). These parameters have the same unit MPa^0.5^ and can be 3D plotted for polymer and solvents. The distance in this ‘Hansen space’ between the polymer and solvent tends to predict the miscibility, with solvents with closer parameters typically being better candidates. This approach has been used to predict drug miscibility in polymers, where the polymer is assumed to be a solvent.

There are several methods to obtain HSPs: group contribution methods (GCMs), solvent experiments, inverse gas chromatography, and other calculation methods derived from physical chemistry calculations. From these methods, GCM and solvent experiments are the most widely used, because they are easy to perform and do not require sophisticated equipment or complex physical chemistry calculations. Examples of GCMs include the Hoftyzer–Van Krevelen (H-VK), Fedors, Just–Breitkreutz [4], Hoy [5], Yamamoto molecular break (Y-MB) [6], and Beerbower [7] methods. These methods account for chemical groups in a given molecule, where each group contributes to HSP values, which then are summed to give HSPs for whole molecule. The most popular GCM is the H-VK, but it should be noted that this is an older method, which accounts mostly for simple chemical groups like hydroxy-, methyl-, benzyl-, etc., while APIs usually consist of heteroatoms and heterocycles that are not present in most GCM tables. In our study, we chose the Y-MB GCM, since this method is based on a large database of molecules and uses artificial intelligence to find HSPs from Simplified Molecular Input Line Entry System (SMILES) notation, which also eliminates human error [8].

Although HSPs have been widely used to predict API–polymer compatibility, the predictions are not always satisfactory, as these parameters do not perfectly predict the possible drug load in a polymer. Studies applying HSPs and GCMs to predict API–polymer miscibility have been compilated in Table 1.

One approach described in the literature to predict drug and polymer miscibility is to use the total difference in Hansen solubility parameters: Δ*σt* (Equations (1) and (2)).

(1) $$\sigma t=\sqrt{{\sigma d}^{2}+{\sigma p}^{2}{+\sigma h}^{2}}$$

Δ*σt* = |*σt*_1_ − *σt*_2_| (2)

where *σd*_1,2_—dispersion force interaction components;

*σp*_1,2_—polar interaction components;
*σh*_1,2_—hydrogen interaction components;
subscripts 1 and 2 representing drug and polymer, respectively.

In this case, it is generally assumed that the drug and polymer will be miscible if Δ*σt* < 7 MPa^1/2^ and immiscible if Δ*σt* > 10 MPa^1/2^. Other studies predicted miscibility between a drug and several kinds of carriers if Δ*σt* < 4.0 [2], <6.8 [1], ≤7 [6,19,21,22], or <13.8 [23]. The use of Δσt is often used and said to be appropriate for melted API–polymer ASDs [19]. Small Δ*σt* values (less than 5 MPa^0.5^) have predicted good drug and polymer miscibility, even achieving highly drug-loaded ASDs up to 80 wt%. On the contrary, other studies using the same Δ*σt* parameter reported unsatisfactory results or no correlation, including reports testing up to 20 formulations meeting the low Δ*σt* criteria, with only one or few formulations being considered miscible [11]. There are several things to consider here. First, there are several GCMs and experimental ways to calculate HSPs, with each approach giving slightly or even highly different Δ*σt* values. Second, it is believed that Δ*σt* calculations work best if there are no strong interactions between the API and polymer, like hydrogen bonds or complex formation [4].

We found fewer studies using the distance values in Hansen space—*D* values (Equation (3)), which represent the three-dimensional distance between the API and polymer in the Hansen solubility graph.

(3) $$D=\sqrt{4{\left(\sigma d1-\sigma d2\right)}^{2}+{\left(\sigma p1-\sigma p2\right)}^{2}{+\left(\sigma h1-\sigma h2\right)}^{2}}$$

*σd* values multiplied by 4—recommended by Hansen, as this better matches the experimental results.

This approach is in accordance with Hansen’s theory and fully involves all three HSP parameters. There are few reports of miscibility prediction using this approach that achieved ASD drug loads of 10–12.6%. However, Lutbow et al. concluded the HSP distance is overall unsatisfactory to predict miscibility [15].

HSP values can be used to calculate the Florry–Huggins interaction parameter *χ* [7,17] or plot the two-dimensional Bagley plot [4]. These calculations have been used to predict drug–polymer miscibility for some samples. Regarding the Florry–Huggins χ parameter, values less than 1 are considered to predict miscibility, while large *χ* values, above 10, tend to predict immiscibility. Values in between seem to be ambiguous.

We found only one study in which the authors used HSP values to calculate the ASD saturation concentration [12]. These calculations require HSP values, experimentally obtained heat capacity, density, and regression analysis. For one given formulation, the predicted ASD saturation concentration was relatively close to the prediction.

In this work, to investigate the correlation of HSPs and amorphous drug loads, the PLA was chosen, since this polymer is one of the polyesters widely used in biomedical and drug delivery applications. Model drugs were chosen to cover the widest possible range of solubility parameters with respect to the PLA.

API concentrations for amorphous phases typically range from 10% up to 70% by weight. Most of the time, the drug loading capacity is expressed as weight percentage, but this approach does not allow one to objectively compare the loading capacities between APIs, since molar concentrations can differ when the molecular weight is taken into consideration. We went further and expressed the drug loading capacity as the summary volume of drug molecules—*Cv* (µL/g) (Equation (4)). This way, all drug-loaded samples can be compared in terms of the total volume drug molecules take up in one gram of polymer (PLA). These *Cv* values were further correlated with the HSP drug–polymer distance (*D*).

*Cv* = *Mv* × *N* × 1000/*m* (4)

*Mv*—molecular volume (cc/mol);

*N*—amount of drug (mol);

m—mass of PLA.

## 2. Materials and Methods

### 2.1. Chemicals and Materials

The following model drugs of pharmaceutical grade were used: ketoprofen (Farmalabor, Assago, Italy), benzocaine (BLD Pharmatech GmbH, Reinbeh, Germany), prednisolone (Sigma Aldrich, Vienna, Austria), metronidazole (TCI, Eschborn, Germany), naproxen (Biosynth, Berkshire, UK), ibuprofen (Supelco, Darmstadt, Germany), celecoxib (BLD Pharmtech, Reinbeh, Germany), diclofenac (Fagron, Kopenhagen, Denmark), piracetam (Sigma Aldrich Vienna, Austria), paracetamol (Sigma Aldrich, Vienna, Austria), lidocaine hydrochloride monohydrate (Biosynth, Berkshire, UK), and indomethacin (TCI, Eschborn, Germany). PLA was purchased from Nature Works (NatureWorks Ingeo Biopolymer 4032D, Naarden, The Netherlands). Solvents were of analytical grade: chloroform, dichloromethane, 1,1-dichloroethane, aniline, tetrahydrofuran, acetonitrile, chlorobenzene, toluene, acetone, dimethyl formamide, ethyl acetate, methanol, dimethyl sulfoxide, formic acid, hexane, diethyl ether, triethyl amine, m-cresol, acetic anhydride, cyclohexyl chloride. For chromatography, we used acetonitrile gradient grade (Fischer Scientific, Waltham, MA, USA), methanol HPLC grade (Honeywell, Charlotte, NC, USA), glacial acetic acid analytical grade (Supelco, Darmstadt, Germany), and phosphoric acid analytical grade (Sigma Aldrich, Vienna, Austria).

### 2.2. Determining HSPs for PLA and Model Drugs

Hansen solubility parameters were established using the Hansen Solubility Parameters in Practice (HSPiP v 6.0.04) software. About ~200 mg of PLA was placed in each test tube; then, ~2 mL of solvent was added to each test tube. PLA was subjected to 17 different solvents: chloroform, dichloromethane, 1,1-dichloroethane, aniline, tetrahydrofuran, acetonitrile, chlorobenzene, toluene, acetone, dimethylformamide, ethyl acetate, methanol, dimethyl sulfoxide, formic acid, hexane, diethyl ether, triethylamine. After 72 h, each test tube was inspected, and each solvent was relatively graded in scale from 1 to 6, where ‘1’ corresponds to completely dissolved, and ‘6’ represents no effect at all. Values 1–3 (dissolves/swells) were considered ‘inside’ the solubility sphere of PLA, while 4–6 (poorly dissolves/no effect), were considered ‘outside’. These values were entered into the software. Then, a solubility sphere check was performed by testing the software-proposed solvents on PLA, this time grading 1 if soluble/swells, and 0 if no effect was observed. The solubility parameters (*σD*, *σP*, *σH*) were calculated using a genetic algorithm.

For model drugs, Yamamoto molecular break (Y-MB) calculations were performed in same software by entering SMILES notations for each model drug. Model drugs were selected so that the HSP distance (*D*) is close, in between, and far away from PLA.

### 2.3. Drug Loading by Solvent Casting

Two grams of PLA were dissolved in 45 mL chloroform; then, 1 mL of this solution was added to a pre-weighed amount of the model drug and vortex mixed until dissolved. For drugs that did not dissolve completely, 100 µL of THF was added and sonicated in a bath. The solution was poured onto glass slide and allowed to evaporate. Finally, the sample was placed in a vacuum drying oven (Jeiotech OV4-30, Jeio Tech, Daejeon, Republic of Korea) at 50 °C and full vacuum for 24 h to remove the residual solvents. Each PLA drug batch was prepared in an incrementally increasing drug load from 0.10 to 2.00 mmol/g. The drug weights used for samples, which were found to be amorphous saturated via solvent casting, are presented in (Table 2).

### 2.4. Drug Loading by Vacuum Compression Moulding (VCM)

A predetermined amount of model drugs was weighed on analytical balances in a 2 mL Eppendorf tube, and 100 ± 0,5 mg of PLA fillings was added; then, 1 mL of chloroform was added, and the tube was vortex mixed, allowed to evaporate, and placed in a vacuum drying oven (Jeiotech OV4-30, Korea) at 50 °C, with full vacuum for 24 h. For VCM (VCM Essentials, MeltPrep GmbH, Graz, Austria), PLA–drug films were cut into pieces and loaded in 8 mm cylindrical die. The heating plate was set to 180 °C, and the sample temperature was monitored using a K-type thermocouple reader (ThermaQ Blue, ETI Ltd., Worthing, UK). Once the temperature in the sample compartment reached 170 °C, it was held there for 5 more minutes; afterwards, the sample compartment was transferred to a cooling plate and cooled with compressed air. Each PLA–drug batch was prepared with an incrementally increasing drug load from 0.10 to 2.00 mmol/g. The drug weights used for samples, which were found to be amorphous saturated via VCM, are found in (Table 3).

### 2.5. Preparation of Physical Mixtures

Physical mixtures of PLA and drugs were prepared by mixing a pre-weighed amount of drug and PLA powder, which was further mixed and ground with a pestle and mortar. Each drug–PLA mixture represents the highest achieved amorphous content of that drug.

### 2.6. XRD Analysis

The samples were tested via XRD after 24 h of their creation. The XRD analysis of samples was done in a reflectance setup using a MiniFlex 600-C (Rigaku Corporation, Tokyo, Japan), 2ϴ range 3–90°, at 10°/min, with a step size 0.01°, lamp voltage 40 kV, lamp current 15 mA, 1.25° divergence slit, and Cu Kα radiation with Cu Kβx1.5 filter. The solvent-casted film samples were placed in a ring-type aluminium sample holder. For VCM samples, parafilm was used to hold the sample tablet in the middle of the same sample holder. For physical mixtures of the drug and PLA powder, a rotating sample stage with a low background silicone sample holder was used.

Data analysis was done using SmartLab studio 64 (v4.6.411.0). Peak detection was done using the ‘peak search’ tool with the following settings—search method: top, Sigma cut: 2, smooth factor 10, peak shape: Split Pseudo-Voigt, with profile fitting and refine background.

### 2.7. FTIR-ATR Analysis

Fourier transformation infrared–attenuated total reflection (FTIR-ATR) analysis was done using (Nicolet IS20, Thermo Fisher Scientific, Waltham, MA, USA) an FTIR equipped with (Quest, Specac Ltd., Orpington, UK) an ATR accessory with a diamond prism. Each sample was pressed against the diamond prism and measured in triplicate with 32 scans and a resolution setting of 4. Each spectrum was corrected using the ‘advanced ATR correction’ algorithm provided in the software (Omnic v9.13.1224). For correction operation, the following settings were selected: prism: diamond, refractive index 1.50, number of bounces 1. Then, an average spectrum from three corrected measurements was created in the same software using the average spectra tool (*Y*-axis).

### 2.8. Chromatographic Analysis of Drug-Loaded PLA Samples

For sample preparation, ~20 mg of the PLA sample was accurately weighed on analytical balances (XA 110.5Y, Radwag, Radom, Poland) in a 15 mL PP centrifuge tube. Then, 1 mL of chloroform was added, and the PLA was allowed to fully dissolve. Afterwards, 5 mL of methanol was added, and the sample tube was vortexed and ultrasonicated in a water bath for 5 min at high setting using Sonorex digiplus (Bandelin electronics, Berlin, Germany). Finally, the solution was filtered into an HPLC vial using 0.22 µm PTFE syringe filters with PP housing and a PP syringe.

For calibration, ~20 mg of each drug was accurately weighed on analytical balances and dissolved in 10.00 mL of methanol to make a stock solution. Calibration solutions in 5 levels were made by taking 50, 150, 250, 350 and 450 µL and diluting each to 1.00 mL volume with methanol.

Regarding the chromatographic conditions, three methods were developed to ensure adequate retention times of all model drugs. The HPLC conditions are summarised in Table 4. Analysis was done on an HPLC equipped with a UV diode array detector (Vanquish, Thermo Fisher Scientific, Waltham, MA, USA).

Linear calibration ranges and limits of quantitation (*LOQ*) (Equation (5)) were assessed from the calibration graphs.

(5) $$\mathrm{LOQ}=\frac{10\;\times \;SDb}{K}$$

where *SDb*—standard deviation of Y axis intercept and *K*—slope of a calibration graph.

To determine the method of recovery, PLA solution (20 mg PLA in 1 mL chloroform) was spiked with 1 mL of the drug–methanol solution to make a representative solution of dissolved sample with a drug concentration equivalent to 5% *w*/*w*. This solution was further treated with 4 mL of methanol (in total 5 mL of methanol added), ultrasonicated, filtered and analysed as previously described. A reference solution of 1 mL chloroform and 4 mL methanol was spiked with same 1 mL drug–methanol solution, filtered and analysed. For each drug, the chromatographic peak area of the spiked PLA solution was compared against the reference solution, with the ratio expressed as the percentage of recovery.

The drug loading was calculated by Formula (4), where the amount of the drug was calculated from Formula (6).

$$N=\frac{6\times C}{Mw}\;(\mathrm{mmol})$$

where C—concentration of prepared sample solution obtained with HPLC and Mw—molecular weight of analysed drug.

### 2.9. Regression Analysis of HSP Distance and Drug Loading Capacity

The drug loading capacity (*Cv*) in PLA was expressed as the total volume taken up by drug molecules per one gram of PLA (Equation (4)). The differences in Hansen solubility parameters D (distance between two points in 3D graph, MPa^0.5^) were calculated using (Equation (3)) for each PLA–model drug pair. To linearise the graph *Cv* vs. *D* the *Cv* values were plotted against 1/*D*, *1*/*D*^2^, and *lgD*.

Regression analysis was done using the in-built excel add-in ‘Analysis ToolPak’, where the ANOVA F-Test was used to calculate *p*-value (Significance F) using a 95% confidence interval. The significance of the linear equations’ intercept value was evaluated from the calculated *p*-value of the intercept. If this value was below 0.05, it was deemed statistically significant and kept; otherwise, the best fit line was calculated through the origin. The coefficient of determination (R^2^) was also calculated.

## 3. Results and Discussion

### 3.1. Determining HSPs for PLA with Solvent Tests

HSPs for PLA were established based on the observed solvent effects (Table 5). Chlorinated solvents dissolved PLA within 2–3 h, graded as 1. Aniline also dissolved PLA, yet not as good as chlorinated solvents, therefore graded as 2. Meanwhile, THF induced noticeable swelling, graded as 3. These solvents were deemed to be ‘inside’ PLA’s solubility sphere. Acetonitrile produced a slightly cloudy solution, but the overall shape and appearance of PLA granules seemed unaffected. After evaporating acetonitrile in a petri dish, a slight amount of PLA residue was left; therefore, this solvent was deemed to be on the edge of PLA’s solubility sphere, graded as 4. Solvents known from the literature to affect different PLA grades but showing no effect in this study were graded as 5, while the remaining solvents with no observable effect were graded as 6. In total, 20 solvents were tested on PLA, including software-proposed solvents for checking the solubility sphere radius.

All the solvents already had HSPs established and were available in the software package. There were no solvents wrongly assigned ‘inside’ or ‘outside’ the solubility sphere, and the model fit of 1.000 gives confidence in the established HSPs for PLA (Table 6).

The solvent grading scale employed in this study provides a semi-quantitative assessment of drug behaviour in selected solvents under standardised preparation conditions. While such grading inherently involves a degree of subjectivity, it reflects experimentally observable outcomes such as complete dissolution, partial solubility, or precipitation, which are directly relevant to formulation screening and process feasibility. The solvent grading scale is not intended to represent equilibrium saturation solubility. Rather, it serves as a rapid and practical indicator of relative drug–polymer affinity and compatibility under conditions encountered during ASD preparation.

### 3.2. Determining HSPs for Model Drugs Using Y-MB GCM

HSPs for model drugs were established using HSPiP software (Table 7). The Y-MB calculation method was chosen, because it is one of the newest GCMs for HSP calculations, it is easy to use, and it is based on thousands of database entries. Other GCMs are generally older versions and do not always provide a clear and understandable way of accounting for chemical groups in many model drugs. The Y-MB model was selected, since model drugs in the current study were chosen to have wide variety of HSPs compared to PLA and have many complicated chemical groups. On the other hand, the Y-MB model also has limitations, such as a lack of information on the calculations and dataset used to establish the model. Therefore, ideally, to test established HSPs, more solvent experiments would need to be done.

### 3.3. Evaluation of Crystalline and Amorphous Phases by XRD

XRD analysis was done to solvent-casted and VCM samples, where each drug had an incrementally higher content. For some samples, visual assessment was enough to determine drug crystallite presence (Figure 1). Samples with the highest drug load before crystal peaks were observed were deemed amorphous saturated (Figure 2 and Figure 3). XRD analysis of these samples show no crystalline drug present (Supplementary Data Figures S1–S12).

**Figure 2:**
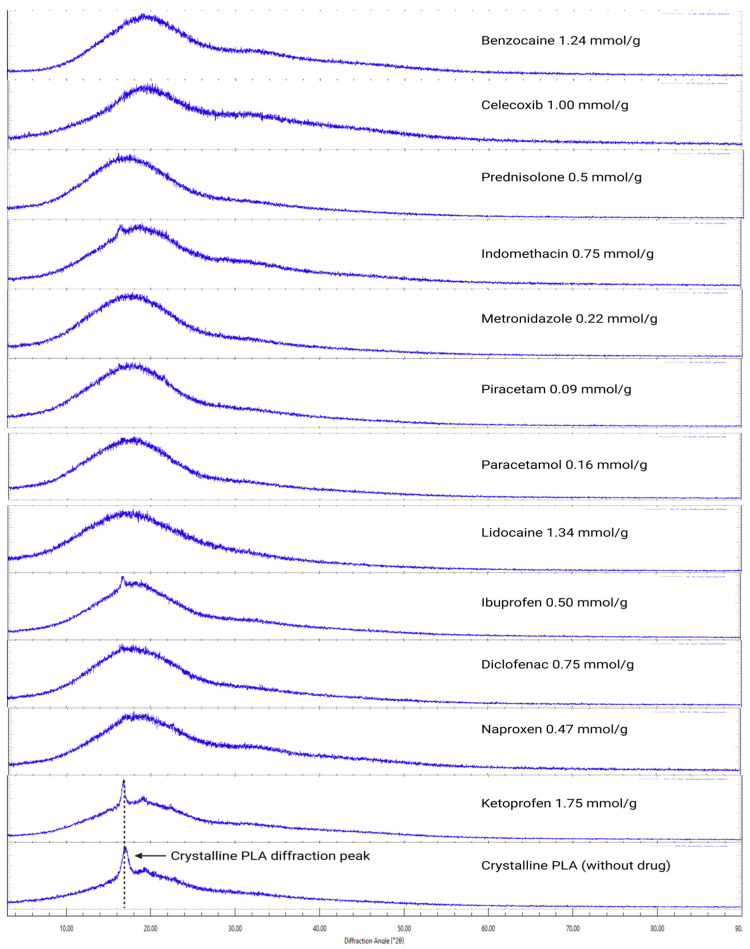
XRD of solvent-casted films.

**Figure 3:**
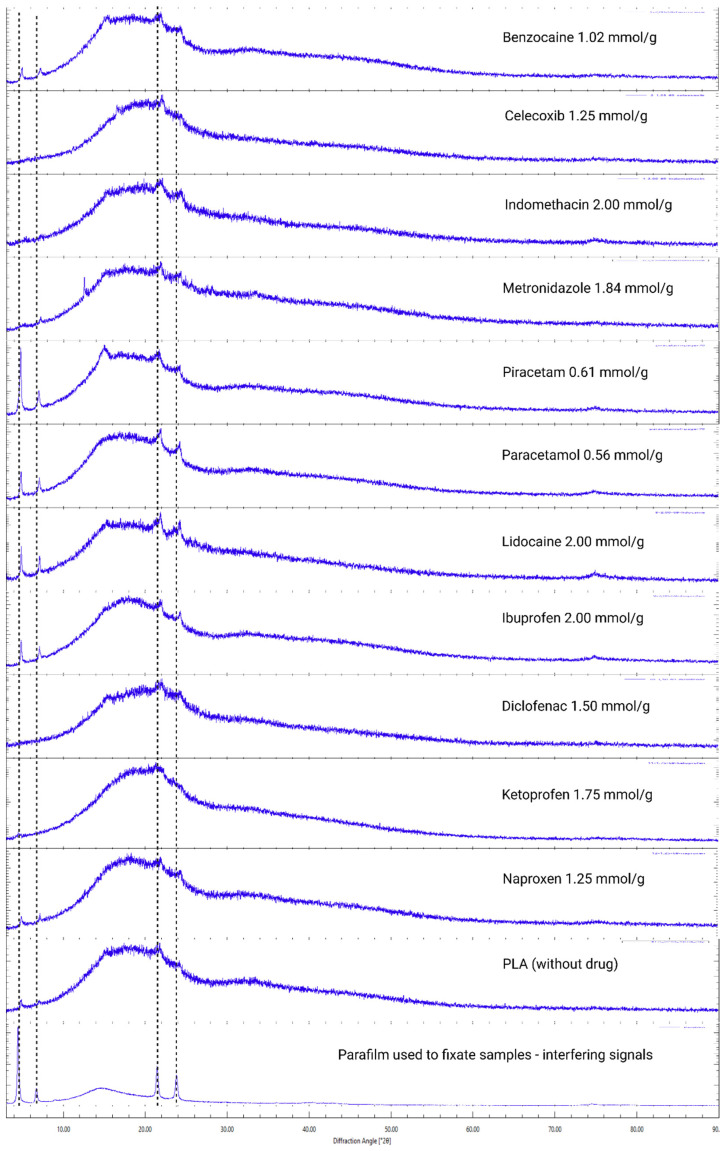
XRD of VCM samples.

XRD diffractograms of the amorphous saturated samples made with solvent casting and VCM contained either no diffraction peaks or peaks present at 2ϴ angles of 17.17° and 20.94, which were recognised as the crystalline PLA phase as seen from blank PLA samples (Figure 2). Diffractograms of VCM samples also contained interfering peaks from parafilm at 2ϴ angles of 4.49, 6.71, 21.47, and 23.84 for parafilm, which was used to hold the VCM (Figure 3) samples during analysis.

Diffractograms of the PLA and drug physical mixtures show the PLA crystalline phase and lower intensity peaks of the crystalline drug. Some drug diffraction peaks in physical mixture diffractograms are absent (Supplementary Figures S1–S13).

The PLA crystalline peak tends to appear right before the drug crystal peaks are observed for higher drug-loaded samples. When the sample is loaded beyond the saturation point of the drug, the formation of drug crystals could induce PLA crystallisation. This has been observed by Qian et al. using hot stage microscopy, where 50% drug-loaded PEG samples showed a portion of polymer crystallised first, followed by the crystallisation of the drug and remaining polymer [24].

### 3.4. FTIR-ATR Analysis

**Figure 4:**
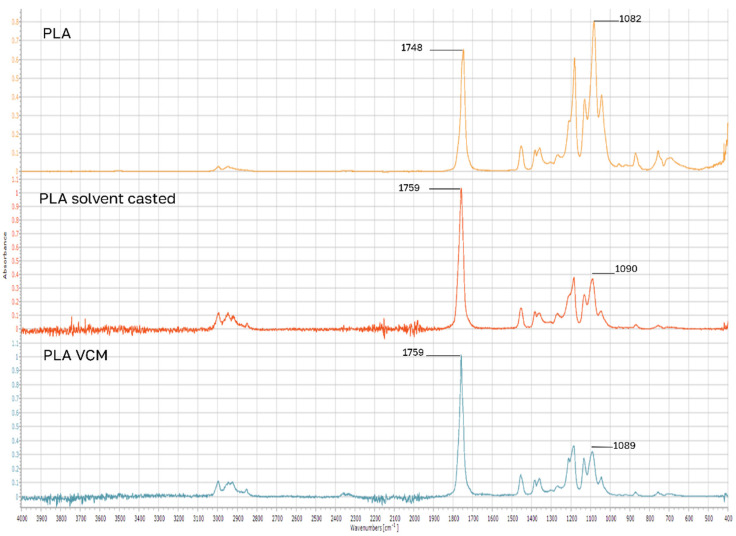
FTIR-ATR analysis of blank PLA samples.

**Figure 5:**
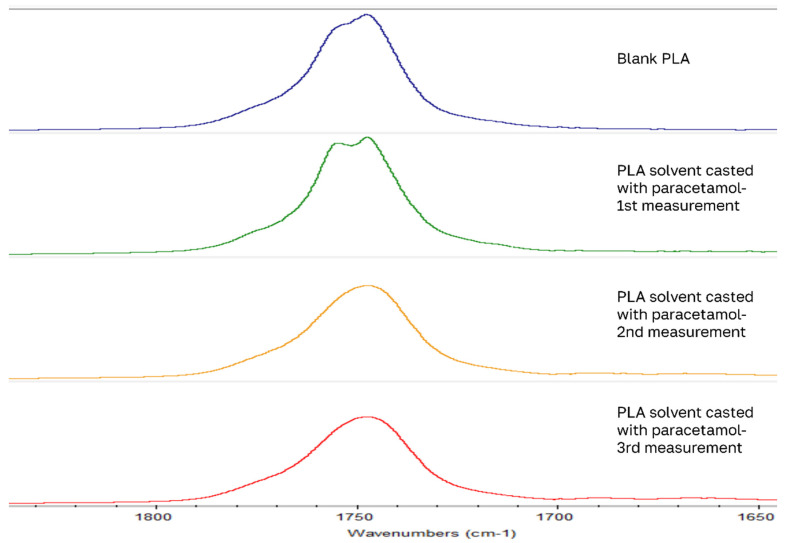
Example of carbonyl peak split.

FTIR-ATR analysis showed IR peak shifts > 10 cm^−1^. Blank PLA samples, containing no drug, show that after solvent casting and VCM workup, the carbonyl double bond stretching peak of 1748 cm^−1^ blue shifts to 1759 cm^−1^, and the ester C-O stretching peak of 1082 cm^−1^ blue shifts slightly to 1090 cm^−1^ (Figure 4). These shifts were also observed for the drug-loaded samples. Since these were the largest IR peak shifts from the PLA chemical groups, we can conclude that solvent casting and the VCM process affect the molecular vibration of the ester group in PLA. While measuring each sample three times, each time repositioning the sample on ATR crystal, we sometimes got a splitting of the carbonyl peak at 1748 (Figure 5). In the literature [25], this is explained as another vibrational mode of the carbonyl group due to crystalline PLA domains. This spectral peak overlap can cause this type of shift.

**Figure 6:**
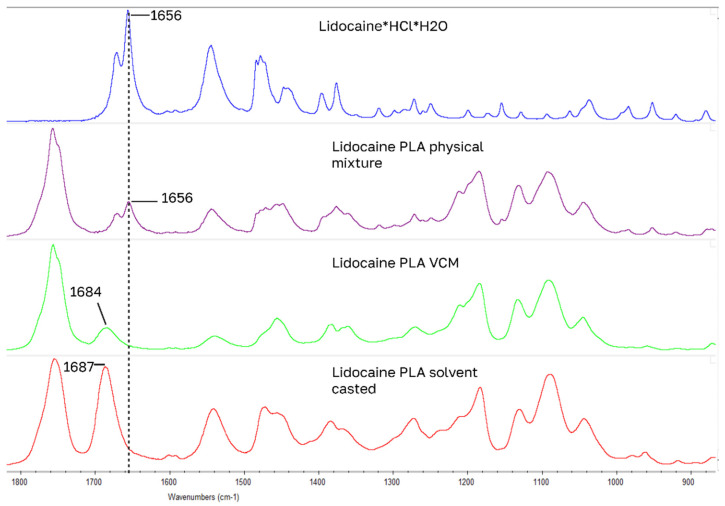
Largest peak shifts in IR spectrum: lidocaine samples.

**Figure 7:**
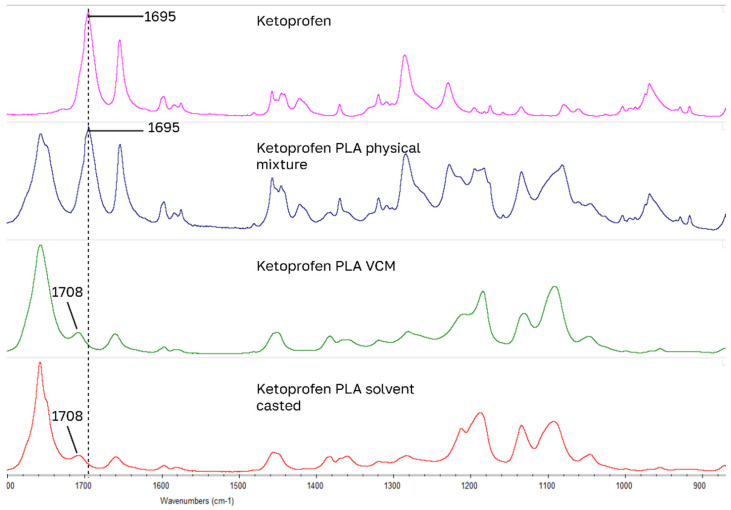
Largest peak shifts in IR spectrum: ketoprofen samples.

**Figure 8:**
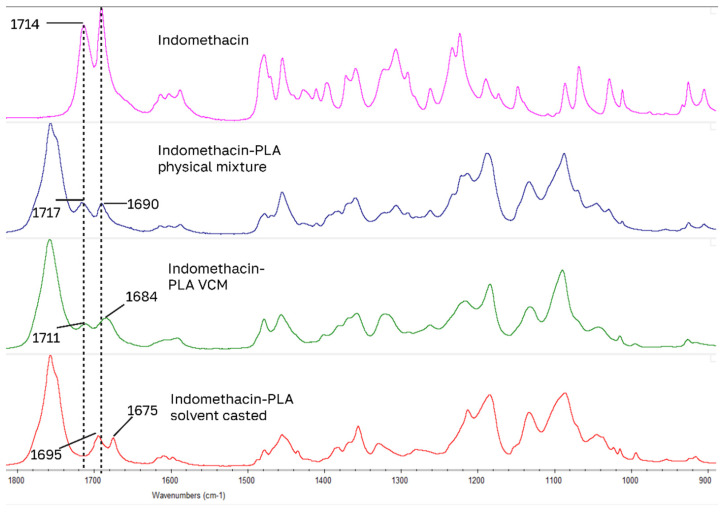
Largest peak shifts in IR spectrum: indomethacin samples.

**Figure 9:**
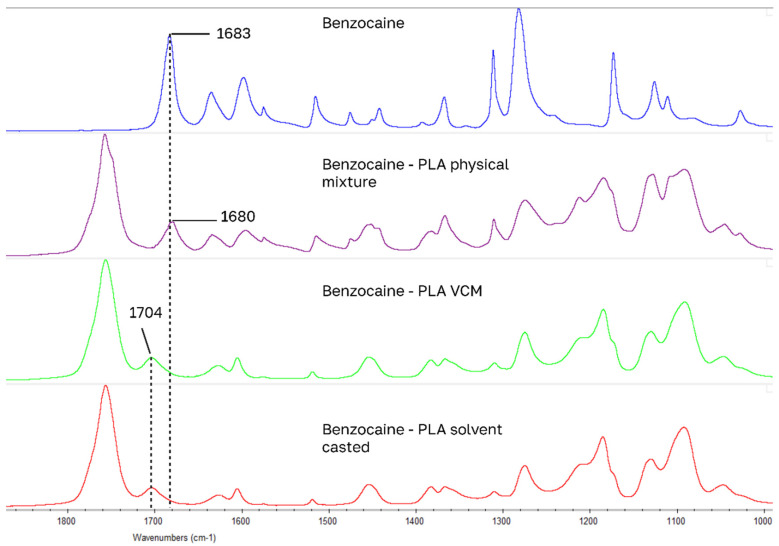
Largest peak shifts in IR spectrum: benzocaine samples.

For drug-loaded PLA samples, the largest IR peak shifts of model drugs occurred in the 1700 cm^−1^ carbonyl stretching region for carboxylic acid and amide functional groups (Figure 6, Figure 7, Figure 8 and Figure 9): for benzocaine (ester C=O 1680 to 1704), indomethacin (carboxylic acid C=O 1714 to 1695 and amide C=O 1691 to 1675), lidocaine (amide C=O 1656 to 1687), ketoprofen (carboxylic C=O 1695 to 1708). Blue shifted spectral peaks could be caused by changes in the loss of intra molecular hydrogen bonding when going from a native crystal structure into PLA. These four drugs have the closest HSPs to PLA.

There is a noticeable decrease in the model drug IR absorption peaks when comparing the PLA–drug physical mixture to solvent-casted or VCM samples (Supplementary data Figures S14–S75). This can be explained by the overall low drug content in PLA and by the lower drug surface content in solvent-casted and VCM samples compared to physical mixtures.

### 3.5. Determination of Drug Amount in Amorphous Saturated Samples by HPLC-UV

The analytical method of development for drug-loaded PLA samples was challenged by the sample preparation step. Firstly, the drug molecules need to be extracted from the PLA matrix; secondly, the final analysed solution should contain no traces of PLA, which would otherwise cause column clogging. This was achieved by employing two miscible solvents, where the first one (chloroform) dissolves the PLA matrix, while the second (methanol) dissolves the drug and precipitates PLA from solution. The evaluated chromatographic performance (Table 8) gave reasonable linearities, recoveries and limits of quantitation to evaluate the drug-loaded PLA samples. The drug content in the PLA samples is compiled in Table 9.

### 3.6. Regression Analysis of HSP Distance and Drug Loading Capacity

**Figure 10:**
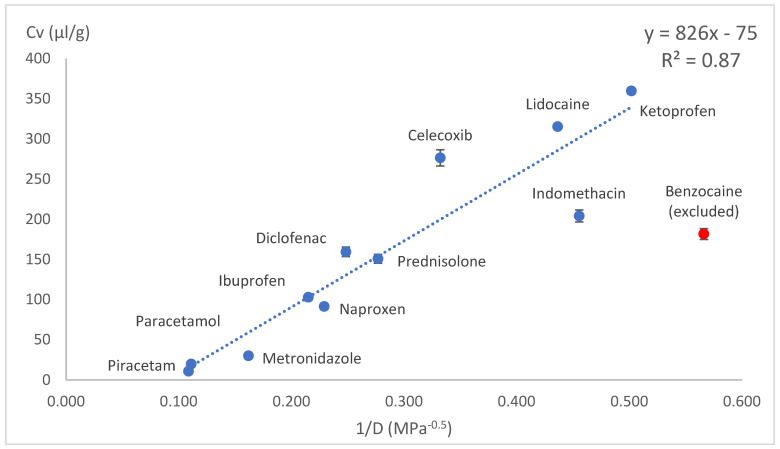
Drug load (Cv) vs. inverse HSP distance between polymer and drug (1/D) in solvent casting.

**Figure 11:**
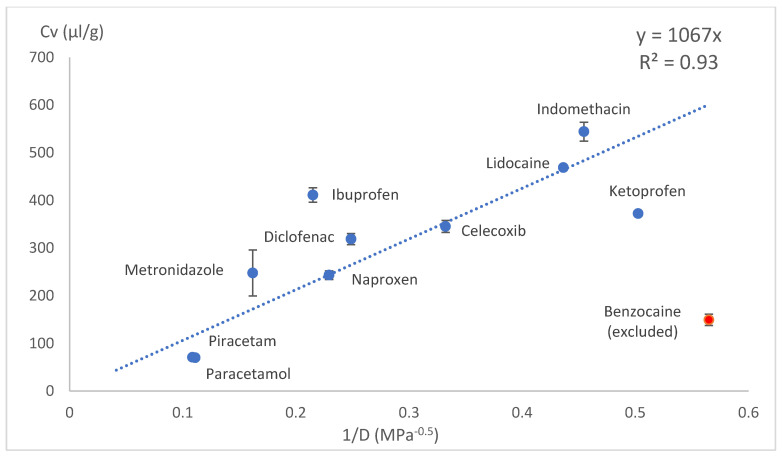
Drug load (Cv) vs. inverse HSP distance between polymer and drug (1/D) in VCM.

**Figure 12:**
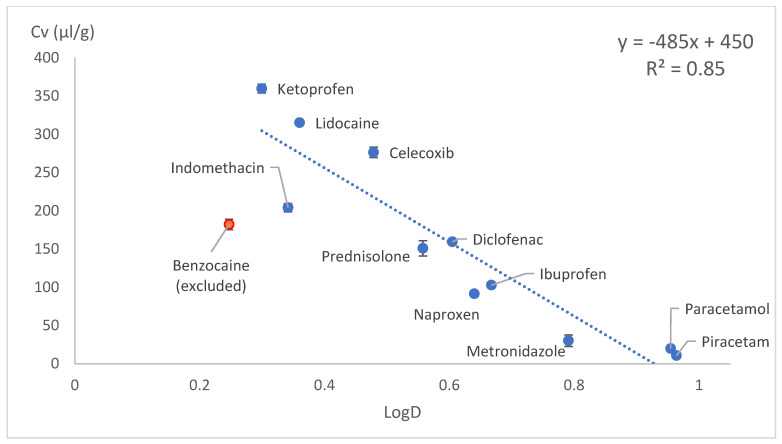
Drug load (Cv) vs. log10 of inverse HSP distance between polymer and drug (LogD) in SC.

**Figure 13:**
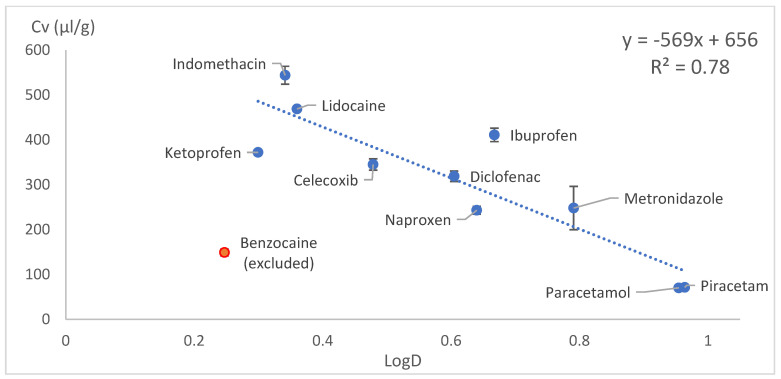
Drug load (Cv) vs. log10 of inverse HSP distance between polymer and drug (LogD) in VCM.

**Figure 14:**
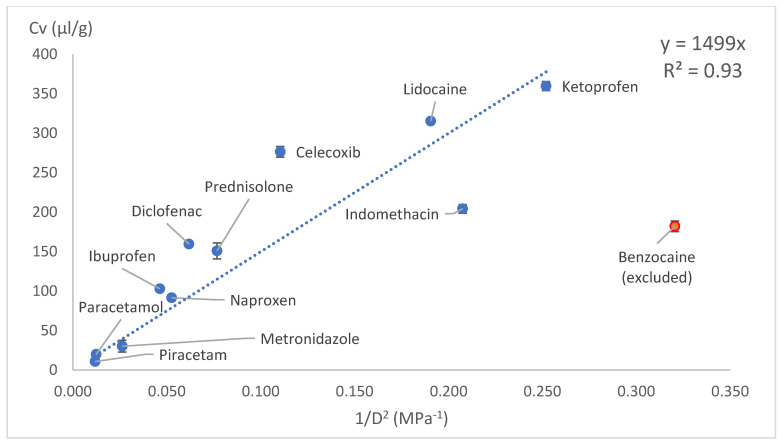
Drug load (Cv) vs. inverse HSP distance between polymer and drug squared (1/D^2^) in SC.

**Figure 15:**
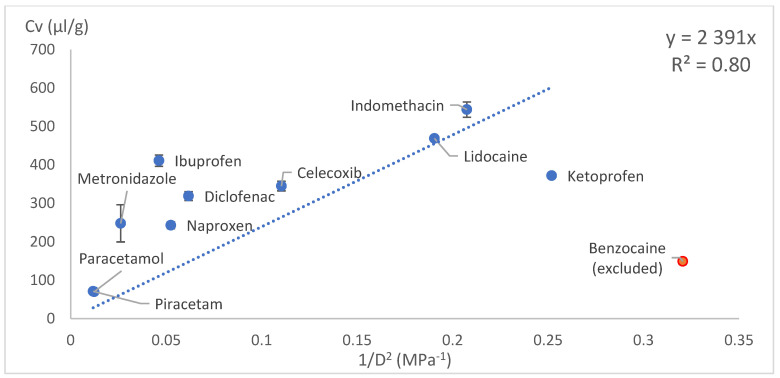
Drug load (Cv) vs. inverse HSP distance between polymer and drug squared (1/D^2^) in VCM.

The drug loading concentration was expressed as the total volume of drug molecules in a given amount of PLA (1 g). Other calculation methods involving Florry–Huggins also use a similar approach, where the solute in a polymer is expressed as the volume fraction. This approach allows a direct comparison of the drug loading capacities between model drugs. When the volumetric concentration (*Cv*) was plotted against the HSP distance (*D*) between the drug and PLA, concave graphs were obtained. These graphs were difficult to express with a mathematical equation; therefore, we tried several linearisation methods: *1/D*, *1/D^2^* and Log(D) on x axes. These kinds of mathematical operations are typical and allow the conversion of data back to original state if needed. Most of the data linearisation methods gave statistically significant linear graphs (Figure 10, Figure 11, Figure 12, Figure 13, Figure 14 and Figure 15). The ANOVA F-Test results compiled in Table 10 show R^2^ values ranging from 0.85 to 0.93 for all statistically significant graphs.

From regular solution theory and Flory–Huggins arguments, the enthalpic penalty of mixing scales increases with increased solubility–parameter mismatch. Using Hansen parameters, this mismatch is represented in a linearised fashion. Since the maximum miscible drug content in a polymer decreases as the interaction parameter χ increases, a nonlinear (concave) decrease in $C_{v}$ with increasing D is expected. Over the experimental range studied here, transforming the distance by 1/D, 1/D^2^, or LogD provides a simple compatibility index that linearises the relationship.

The volumetric loading capacity (*C*_v_) reported in this study is defined as the maximum experimentally achievable drug volume fraction in the polymer matrix prior to the onset of detectable phase separation or crystallisation. While *C*_v_ is expressed in volumetric terms and is therefore formally comparable to the drug volume fraction used in Flory–Huggins theory, it is important to emphasise that *C*_v_ is not a direct thermodynamic state variable obtained from free energy minimisation. In Flory–Huggins theory, the equilibrium drug volume fraction is determined by the condition of equal chemical potentials between coexisting phases and depends explicitly on the interaction parameter χ, which represents the enthalpic penalty of mixing. In contrast, *C*_v_ represents an experimentally accessible saturation limit that reflects the combined influence of thermodynamic driving forces and kinetic constraints, including limited molecular mobility, processing history, and time-dependent crystallisation phenomena.

Benzocaine showed plasticising properties, and it was not possible to load it into PLA in the amounts predicted by linear equations. Benzocaine was excluded from the linear models. This exception could be explained by benzocaine’s plasticising effects on PLA, as it decreased PLAs Tg from 55 °C (manufacturer reported) to somewhat below room temperature. The mixture of benzocaine and PLA showed the behaviour of an eutectic mixture with a melting point around 120 °C. This means increased polymer chain mobility, which allows drug molecules to move freely and crystalise. The benzocaine-loaded samples were unstable and became crystalline within few hours. Regarding VCM, prednisolone was excluded from this experiment due to the high melting point of 230 °C.

From the achieved drug loading capacities, the VCM method achieves higher amorphous drug loads compared to solvent casting. The higher maximum amorphous loadings achieved by VCM compared with solvent casting are attributed to fundamental differences in processing conditions. Solvent casting is constrained by drug solubility in the chloroform-rich casting solution and by kinetic demixing during solvent evaporation, which can induce early drug-rich domain formation and/or crystallisation, particularly for the more polar model drugs. In contrast, VCM involves melt processing under vacuum, where increased polymer chain mobility and faster diffusion enhance drug–polymer mixing and apparent solubility in the melt; subsequent rapid cooling kinetically traps a saturated amorphous drug phase.

## 4. Conclusions

Here, we report on the novel use case of Hansen solubility parameters, where we found a significant correlation between the drug saturation concentration and drug–polymer distance in Hansen solubility space, using Y-MB calculations and solvent tests on the polymer. When used within a reasonable drug loading range and excluding highly plasticising drug molecules, this approach could potentially serve as screening method for approximating the amorphous saturation of a drug in a given polymer. To validate this approach, more polymers need to be tested, and a longer stability time needs to be evaluated.

The relatively small number of drug–polymer systems investigated in this study reflects the experimental complexity and time requirements associated with preparing and characterising ASDs using multiple processing routes. Accordingly, the proposed relationship between *C*_v_ and the Hansen distance is intended as a mechanistically informed exploratory model, rather than a purely data-driven predictive tool derived from large training datasets. The selected compounds span a wide range of molecular weights, functional groups, and Hansen solubility parameters, ensuring that the analysed dataset covers a broad compatibility spectrum despite the limited sample size. It should be noted that the present study does not include external validation using drugs not employed in model development. Nevertheless, the current findings establish a physically interpretable framework that can guide rational formulation screening and inform the selection of additional compounds for subsequent predictive model development.

This use of Hansen solubility parameters seems not to work when plastification of PLA becomes an issue. Highly plasticising drugs substantially reduce the glass transition temperature of PLA, which may compromise the physical stability and increase the risk of recrystallisation during storage. Such APIs may therefore be unsuitable for stable PLA-based amorphous systems unless drug loading is restricted or additional stabilisation strategies are employed. Consequently, highly plasticising drugs may need to be excluded from PLA-based ASD development. Furthermore, the present work focused on single-polymer systems and short-term amorphous confirmation. Long-term physical stability studies under relevant storage conditions are necessary to verify the predictive value of our model. In addition, for APIs exhibiting limited miscibility or excessive plasticisation, screening of alternative polymers or polymer blends may be required.

## Figures and Tables

**Figure 1:**
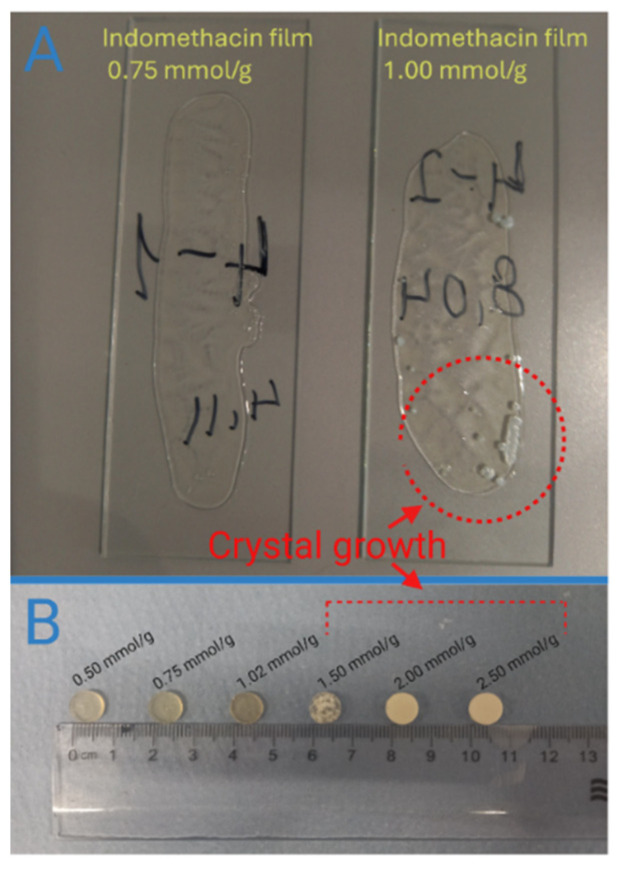
Visually assessed sample examples. (**A**)—Indomethacin solvent-casted films, (**B**)—benzocaine VCM sample.

**Table 1:** Studies using HSPs and GCMs to predict API–polymer miscibility.

Model Drug	Polymer	Drug Loading Method	HSPs Used	Conclusions/Remarks	Ref.
Ibuprofen	KOLVA64, KOL17PF, HPMCAS, Eudagrit EPO	No drug loading performed	Fedors, H-VK, Just–Breitkreutz	Drug and polymer are assumed to be miscible if total difference in HSPs < 7.0 MPa^1/2^. Provides good approximations only if the weak interactions are predominant.	[4]
Cimetidine, Sulphanilamide, Indomethacin, Ketoconazole	Kollidon 12 PF, Polyvinyl acetate	Melt quenching	H-VK	Drug and polymer combinations with small total difference in HSPs (<2 MPa^0.5^) or very large difference (>10 MPa^0.5^) gave reliable predictions of miscibility, with one exception.	[9]
Ibuprofen, Fenofibrate, Clonazepam, Alprazolam	PVP64 (non-ionic), HPMC (non-ionic), EPO (ionic)	Hot melt extrusion	Total HSP difference calculated using molecular dynamics simulation	HSPs predicted 10 drug–polymer combinations miscible, yet only three were successfully miscible: ibuprofen and fenofibrate in PVP64 and ibuprofen in EPO.	[10]
Indomethacin, Itraconazole, Griseofulvin	Eudragit EPO, Eudragit L-100-55, Eudragit L-100, HPMCAS-LF, HPMCAS-MF, Pharmacoat 603, Kollidon VA-64	Hot melt extrusion	H-VK	All 21 drug–polymer combinations had total HSP difference < 7 MPa^0.5^; only itraconazole and Eudagrit in EPO were experimentally immiscible.	[11]
Paliperidone	Poly(lactide-co-glycolide)	Microsphere preparation by emulsion solvent evaporation	Experimental solvent tests on polymer and API	Drug–polymer distance in Hansen space was 5.05 MPa^0.5^, where API lies within polymers solubility sphere (radius 7.8 MPa^0.5^). API solubility in polymer calculated mathematically 12.6% (*w*/*w*), DSC results showed 13–16% amorphous drug load.	[12]
Norfloxacin	Kollidon SR	Hot melt extrusion	Fedors, H-VK, Hoy	HSPs predict good drug and polymer miscibility and stability based on distance in Hansen space < 7 Mpa^0.5^, with 10% (*w*/*w*) drug load achieved.	[5]
Curcumin, Vinpocetine, Osthole, Resveratrol, Oridonin	Chitosan-grafted monooleate, Hyaluronic acid-grafted glyceryl monooleate, Dextran-grafted vitamin E succinate	Ultrasound-assisted maceration	Beerbower GCM used to calculate Flory–Huggins interaction parameters—*χ*	Drug and polymer Flory–Huggins *χ* parameter calculated separately for hydrophilic and hydrophobic parts of polymer. Drugs having good compatibility with the hydrophobic part and suitable compatibility with the hydrophilic part may have higher loading capacity.	[7]
5-Fluorouracil, Curcumin	Various lipids	Dissolving drug in lipid solution	H-VK and Greenhalgh GCMs	Distance between drug and lipid in Hansen space calculated using H-VK < 10 MPa^0.5^ and Greenhalgh < 5 MPa^0.5^ shows correlation with experimental solubility data.	[13]
Glibenclamide	Soluplus, Poloxamer 407, PEG 6000, HPMC	Solvent evaporation, Hot melt extrusion	H-VK	Soluplus and poloxamer had lowest total difference in HSPs (9.79, 8.91 MPa^0.5^) and produced highest percentage of amorphous content (60–66% and 43–63%).	[14]
Curcumin, Paclitaxel, Dexamethasone, Efavirenz, Tanshinone IIA	18 triblock copolymers, Soluplus, Resomer	Polymer micelles prepared by thin film method	H-VK, Y-MB, Fedors, solvent experiments	Drug–polymer distance in Hansen space able to correctly predict some trend regarding good and poor solubilisers, yet the overall predictive strength of Hansen solubility parameters are said to be clearly unsatisfactory.	[15]
Tacrolimus	Hydroxypropyl cellulose, Ethylcellulose, Soluplus, PEG 6000, Poloxamer-188 (Koliphor-188), Eudragit S100	Solvent evaporation	Y-MB	HSP calculations were useful in many cases, but there was also overestimation for some polymers to be miscible with the model drug tacrolimus.	[6]
Coumarin 6, Dexamethasone, Vorinostat/SAHA, Lutein	Poly(ε-caprolactone-b-ethylene oxide), Poly(styrene-b-ethylene oxide)	Three nano-precipitation processes: batch sonication, continuous flow flash, electrohydrodynamic mixing-mediated	H-VK, Te Nijenhuis	HSPs were compared to polymers’ encapsulation efficiency. Total difference in HSPs showed no correlations, with some correlations using σH parameter. HSP distance values in Hansen space were not predictive of encapsulation efficiency in experimental tests.	[16]
Phenylbutazone, Sucrose, Chloramphenicol	PEG 6000	Melting	Fedors GCM used to calculate Florry–Huggins interaction parameter *χ*	Possible to differentiate polymer immiscible drugs but cannot differentiate completely miscible from partially miscible drugs.	[17]
Ritonavir	Eudragit S100, HPMCAS-L, HPMCAS-H, PEG-6000, PVP-VA, Soluplus	Film casting	H-VK	Total HSP difference predicted miscibility with all polymers (ranged 2.9–3.4 MPa^0.5^) except PEG 6000 (12.54 MPa^0.5^). Prepared ASDs up to 40% drug load were possible, with the exception of PEG 6000.	[18]
Indomethacin, Lacidipine	PEG 8, PEG 10, PVP 12, PVP 30, PVP/VA, PVA	Hot melt extrusion	Average of Hoy and H-VK	Total difference in HSPs (<2 MPa^0.5^) predict all polymers to be miscible with both drugs, confirmed by DSC, hot stage microscopy and hot melt extrusion. Drug loads ranged from 50 to 80% (*w*/*w*).	[19]
Ibuprofen	Poloxamer 188, Polysorbate (20, 40,60, 80), Polyoxyethylene 40 stearate	Solution evaporation, melting	Hildebrand parameters obtained from vaporisation energies, molar volume obtained from Fedors	Drug miscibility increases with decreasing difference in Hildebrand solubility parameter.	[20]

**Table 2:** Drug amounts used to prepare amorphous solvent-casted samples.

Drug	Nominal Weight, (mg per 1 mL PLA Solution)	Nominal Cm, (mmol of Drug per 1 g of PLA)	Nominal Cv (µL of Drug per 1 g of PLA)	Nominal Drug Weight % Cw
Benzocaine	9.18	1.25	183	20.6
Celecoxib	16.95	1.00	276	38.1
Prednisolone	8.00	0.50	151	18.0
Indomethacin	11.9	0.75	204	26.8
Metronidazole	9.50 *	0.25	34	4.3
Piracetam	3.15 *	0.10	12	1.4
Paracetamol	5.35 *	0.15	19	2.3
Lidocaine	17.33 **	1.35	316	31.6
Ibuprofen	4.58	0.50	103	10.3
Diclofenac	6.58	0.50	106	14.8
Ketoprofen	19.78	1.75	372	44.5
Naproxen	5.12	0.50	97	11.5

* Solution made in 5 mL PLA chloroform solution due to impracticality of weighing tiny amounts of drug. ** Weight adjusted for lidocaine hydrochloride monohydride.

**Table 3:** Drug amounts used to prepare amorphous VCM samples.

Drug	Nominal Weight, (mg per 1 mL PLA Solution)	Nominal Cm, (mmol of Drug per 1 g of PLA)	Nominal Cv (µL of Drug per 1 g of PLA)	Nominal Drug Weight % Cw
Benzocaine	16.52	1.00	147	16.5
Celecoxib	47.67	1.25	345	47.7
Prednisolone *	-	-	-	-
Indomethacin	71.56	2.00	544	71.6
Metronidazole	31.66	1.85	249	31.7
Piracetam	8.53	0.60	70	8.5
Paracetamol	9.07	0.60	75	9.1
Lidocaine	57.77 **	2.00	469	46.9
Ibuprofen	41.26	2.00	411	41.3
Diclofenac	44.42	1.50	319	44.4
Ketoprofen	44.50	1.75	372	44.5
Naproxen	28.78	1.25	243	28.8

* Excluded from VCM experiments due to its high melting point. ** Weight adjusted for lidocaine hydrochloride monohydride.

**Table 4:** HPLC conditions for drug determination in PLA.

Model Drugs	Column	Flow Rate, mL/min	Column Temperature, °C	Gradient	Mobile Phases	UV Wavelength, nm	Injection Volume, µL
Benzocaine Celecoxib Prednisolone Indomethacin Ibuprofen Diclofenac Ketoprofen Naproxen	Waters xSelect CSH, C18, 3 × 150 mm, 2.5 µm	0.35	40	80% A 10% B for 5 min. B increased to 50% and A decreased to 40% over 10 min. C held at 10% whole analysis	A: Water pH = 2 with H_3_PO_4_ B: Acetonitrile C: Methanol	294 256 243 320 220 275 259 234	2
Metronidazole Piracetam Paracetamol	Waters XBridge BEH C18, 4.6 × 100 mm, 2.5 µm	0.35	40	Isocratic 90% A, 10% B	A: Water B: Methanol	311 214 243	2
Lidocaine	Waters Xbridge BEH Phenyl, 4.6 × 150 mm, 2.5 µm	0.7	40	Isocratic 75% A 25% B	A: 50 mmol acetic buffer pH = 5.5 B: Acetonitrile	254	2

**Table 5:** Graded solvent effects on PLA.

Solvents	Grade (Solubility Sphere)	Comment
Chloroform, Dichloromethane, 1,1-dichloroethane	1 (inside)	Completely dissolved
Aniline	2 (inside)	Dissolves into viscous liquid
Tetrahydrofuran	3 (inside)	Swells
Acetonitrile	4 (outside)	Slightly cloudy solution, tiny amount of residue after evaporation
Chlorbenzene, Toluene, Acetone, Dimethylformamide, Ethyl acetate	5 (outside)	Evidence from the literature to affect different PLA grades, but no observable effect in our study
Methanol, Dimethylsulfoxide, Formic acid, Hexane, Diethylether, Triethylamine	6 (outside)	No observable effect
**Solubility sphere check**		
m-cresol	1 (inside)	Swells
Acetic anhydride, Cyclohexyl chloride	0 (outside)	No observable effect

**Table 6:** Hansen solubility parameters obtained for PLA.

Parameter	Value
*σD*, MPa^0.5^	18.9
*σP*, MPa^0.5^	8.0
*σH*, MPa^0.5^	7.6
*σTotal*, MPa^0.5^	21,9
Solubility sphere radius, MPa^0.5^	6.6
Fit	1000
Wrong solvents inside sphere	0
Wrong solvents outside sphere	0
Total solvents tested	20

**Table 7:** Y-MB calculations for model drugs.

Model Drug	*σD*, MPa^0.5^	*σP*, MPa^0.5^	*σH*, MPa^0.5^	D (Drug-PLA) MPa^0.5^	*MVol*, cc/mol
Benzocaine	18.7	9.4	8.6	1.766	146.5
Celecoxib	19.7	10.5	7.1	3.010	276.3
Prednisolone	19.0	7.8	11.2	3.611	301.7
Indomethacin	19.7	6.5	7.7	2.195	272.1
Metronidazole	19.7	11.8	12.2	6.177	134.6
Piracetam	19.2	15.7	12.6	9.201	116.2
Paracetamol	19.9	13.3	14.6	9.005	125
Lidocaine	17.9	7.5	6.6	2.291	234.4
Ibuprofen	17.5	4.3	7.9	4.650	205.6
Diclofenac	20.4	6.8	10.0	4.025	212.6
Ketoprofen	19.3	6.2	7.9	1.992	212.8
Naproxen	19.0	4.5	10.2	4.365	194.5

**Table 8:** HPLC-UV method testing results.

Drug	Calibration Range, µg/mL	Linearity Coefficient, R^2^	Limit of Quantitation, wt%	Recovery, %
Benzocaine	100–1500	0.9993	0.84	89.9
Celecoxib	100–1500	0.9994	1.3	102.3
Prednisolone	100–1550	0.9995	0.96	94.2
Indomethacin	100–1500	0.9997	2.8	103.8
Metronidazole	100–1550	0.9998	1.2	99.3
Piracetam	100–1500	0.9997	0.7	101.6
Paracetamol	100–1550	0.9996	2.1	102.3
Lidocaine	100–1500	0.9998	0.81	99.0
Ibuprofen	100–1500	0.9997	2.7	97.6
Diclofenac	100–1500	0.9996	3.45	93.9
Ketoprofen	100–1150	0.9997	1.2	104.1
Naproxen	100–800	0.996	1.5	112.2

**Table 9:** Maximum amorphous drug load capacities achieved.

Model Drug	Highest Drug Load Achieved with Solvent Casting				Highest Drug Load Achieved with VCM			
	*C molar* mmol/g_PLA_	*C weight*, wt%	*Cv*. µL/g_PLA_	RSD.%	*C molar* mmol/g_PLA_	*C weight*, wt%	*Cv*. µL/g_PLA_	RSD.%
Benzocaine *	1.24	20.5	182	7.5	1.02	16.8	149	5.3
Celecoxib	1.00	38.1	276	7.3	1.25	47.7	345	7.3
Prednisolone **	0.50	18.0	151	7.3	-	-	-	-
Indomethacin	0.75	26.8	204	7.3	2	71.6	544	7.3
Metronidazole	0.22	3.82	30	7.3	1.84	31.5	248	39
Piracetam	0.09	1.31	11	7.3	0.61	8.7	71	6.7
Paracetamol	0.16	2.39	20	7.3	0.56	8.5	70	1.9
Lidocaine	1.34	31.5	315	1.35	2.0	46.9	469	1.4
Ibuprofen	0.50	10.3	103	7.3	2.0	41.3	411	7.3
Diclofenac	0.74	22.2	159	7.3	1.5	44.4	318.9	7.3
Ketoprofen	1.69	43.0	360	1.3	1.75	44.5	372	1.3
Naproxen	0.47	10.8	91	7.5	1.25	28.8	243	7.3

* Excluded from mathematical models due to high plasticisation. ** Excluded from VCM due to its high melting point.

**Table 10:** ANOVA F-test results for data linearisation.

Linearisation Method	Drug Loading Method	Statistic	Value	Conclusion
1/D (with intercept)	Solvent casting	R^2^	0.87	
		Significance F	2.73 × 10^−5^	Linear model is statistically significant (F < 0.05)
		Intercept *p*-value	0.048	Intercept is statistically significant (*p*-value < 0.05)
		Coefficient	826.034	
		Intercept	−74.601	
1/D (without intercept)	Solvent casting	R^2^	0.93	
		Significance F	4.81 × 10^−7^	Linear model is statistically significant (F < 0.05)
		Intercept *p*-value	N/A	
		Coefficient	606.797	
		Intercept	N/A	
1/D (with intercept)	VCM	R^2^	0.68	
		Significance F	0.003261	Linear model is statistically significant (F < 0.05)
		Intercept *p*-value	0.404814	Intercept is not statistically significant (*p*-value > 0.05)
		Coefficient	893.426	
		Intercept	59.130	
1/D (without intercept)	VCM	R^2^	0.93	
		Significance F	7.65 × 10^−6^	Linear model is statistically significant (F < 0.05)
		Intercept *p*-value	N/A	
		Coefficient	1067	
		Intercept	N/A	
1/D^2^ (with intercept)	Solvent casting	R^2^	0.81	
		Significance F	1.45 × 10^−4^	Linear model is statistically significant (F < 0.05)
		Intercept *p*-value	0.233438	Intercept is not statistically significant (*p*-value > 0.05)
		Coefficient	1296.892	
		Intercept	32,86	
1/D^2^ (without intercept)	Solvent casting	R^2^	0.92	
		Significance F	7.04 × 10^−7^	Linear model is statistically significant (F < 0.05)
		Intercept *p*-value	N/A	
		Coefficient	1498.805	
		Intercept	N/A	
1/D^2^ (with intercept)	VCM	R^2^	0.568	
		Significance F	0.011	Linear model is statistically significant (F < 0.05)
		Intercept *p*-value	0.008958	Intercept is statistically significant (*p*-value < 0.05)
		Coefficient	1327.632	
		Intercept	180.272	
1/D^2^ (without intercept)	VCM	R^2^	0.801	
		Significance F	0.000196	Linear model is statistically significant (F < 0.05)
		Intercept *p*-value	N/A	
		Coefficient	2390.871	
		Intercept	N/A	
Log(D) (with intercept)	Solvent casting	R^2^	0.85	
		Significance F	4.799 × 10^−5^	Linear model is statistically significant (F < 0.05)
		Intercept *p*-value	2.47 × 10^−6^	Intercept is statistically significant (*p*-value < 0.05)
		Coefficient	−484.520	
		Intercept	449.674	
Log(D) (with intercept)	VCM	R^2^	0.785	
		Significance F	6.47 × 10^−4^	Linear model is statistically significant (F < 0.05)
		Intercept *p*-value	1.2 × 10^−5^	Intercept is statistically significant (*p*-value < 0.05)
		Coefficient	−569.161	
		Intercept	656.424	

## Data Availability

The data presented in this study are available on request from the corresponding author.

## Supplementary Materials

The following supporting information can be downloaded at: https://www.mdpi.com/article/10.3390/pharmaceutics18030396/s1, Figures S1–S13: “XRD difractograms of samples”, Figures S14–S75: “IR spectra of sample”; Table S1: “XRD peak list”.
