# Supplementary material for: Predicting Drug Loading Capacity for PLA-Amorphous Drug System Using Hansen Solubility Parameters
Source: Pharmaceutics. 2026 Mar 23;18(3):396. doi: 10.3390/pharmaceutics18030396 (PMC13030427; doi:10.3390/pharmaceutics18030396)
Supplement: Supplementary file 1 [file pharmaceutics-18-00396-s001.zip › pharmaceutics-4109247-supplementary.pdf]

Supplementary Materials: Predicting Drug Loading Capacity for PLA-Amorphous Drug System Using Hansen Solubility Parameters

Artūrs Paulausks, Artjoms Iljičevs, Jurga Bernatoniene, Līga Pētersone and Konstantīns Logviss

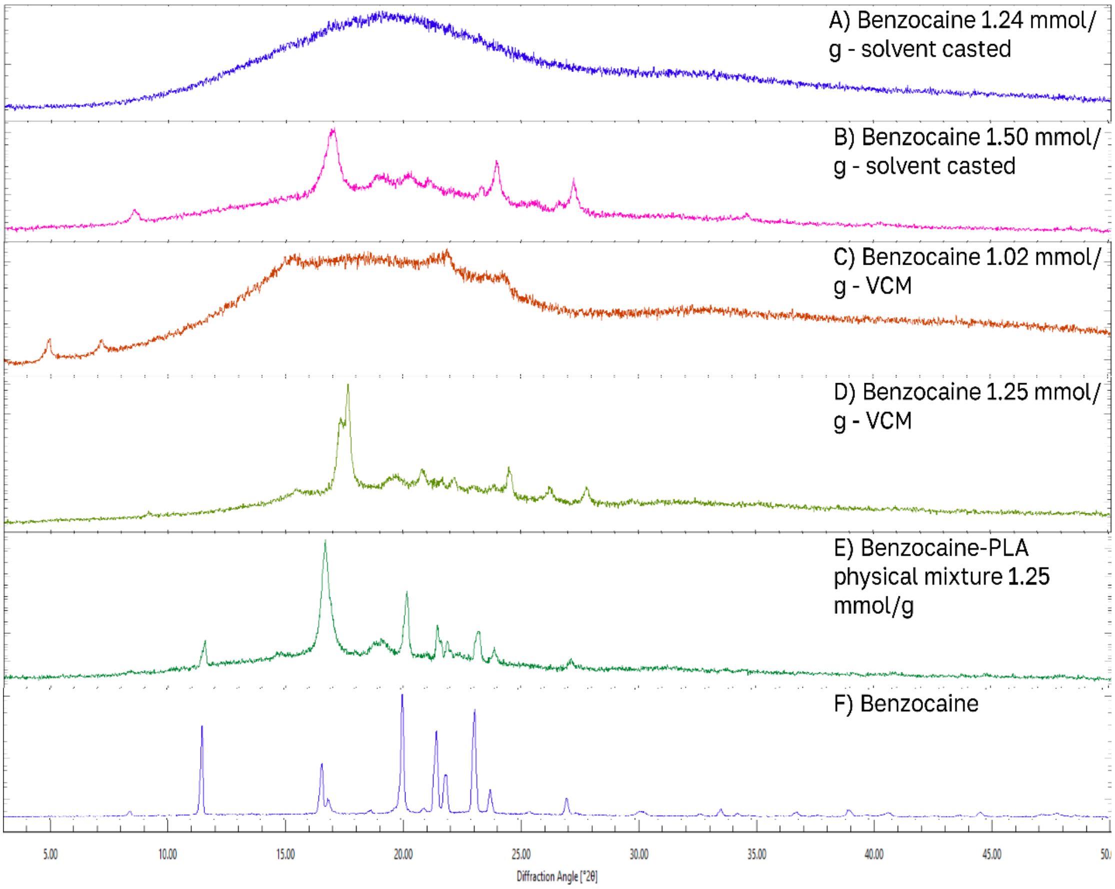

Figure S1. – XRD diffractograms of benzocaine samples.

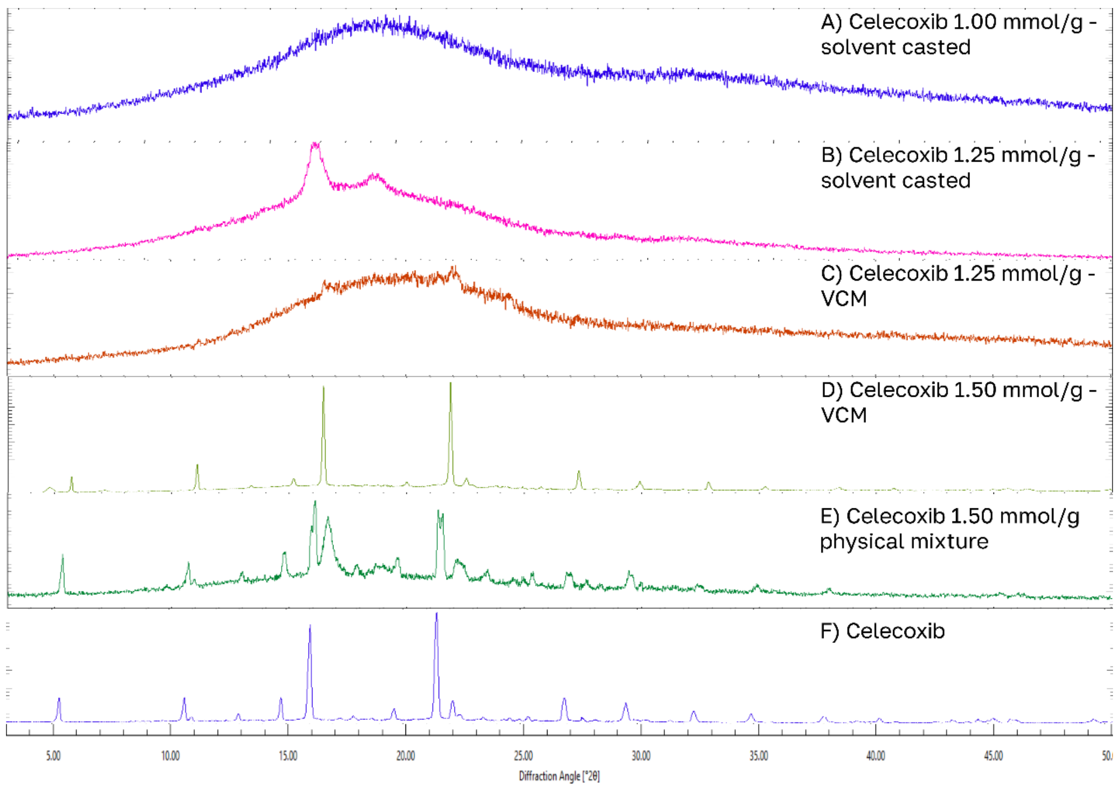

Figure S2. – XRD diffractograms of celecoxib samples.

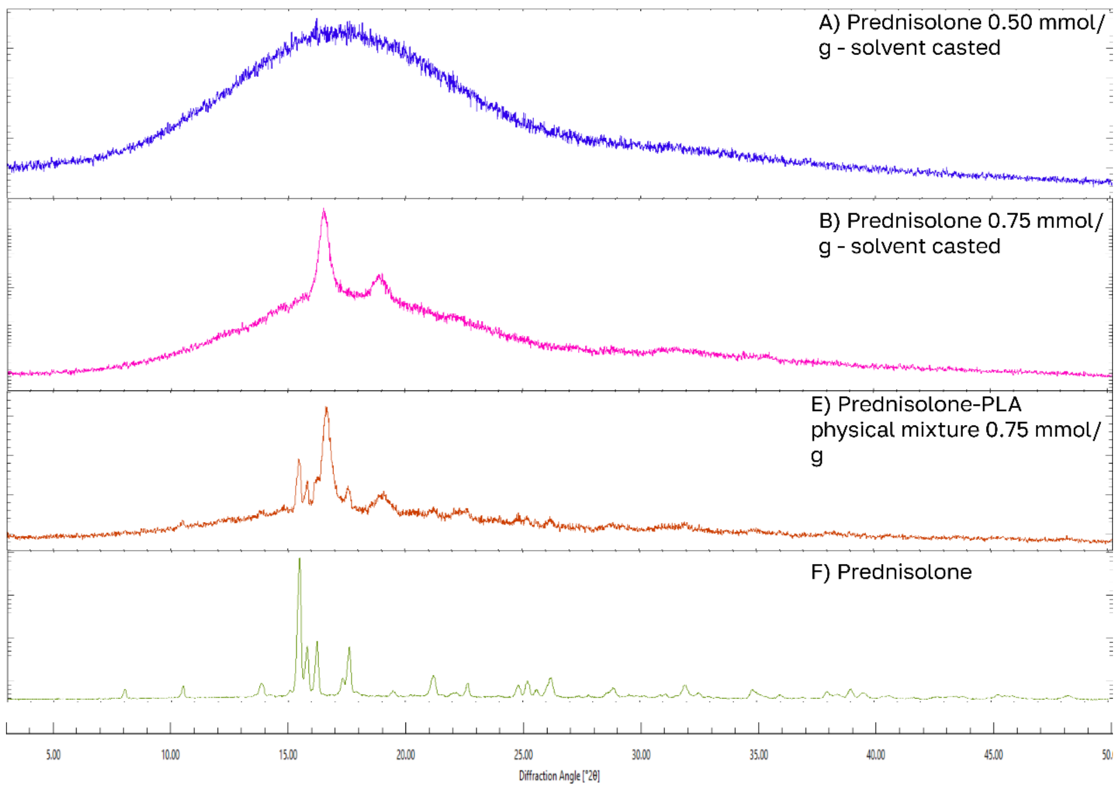

Figure S3. – XRD diffractograms of prednisolone samples.

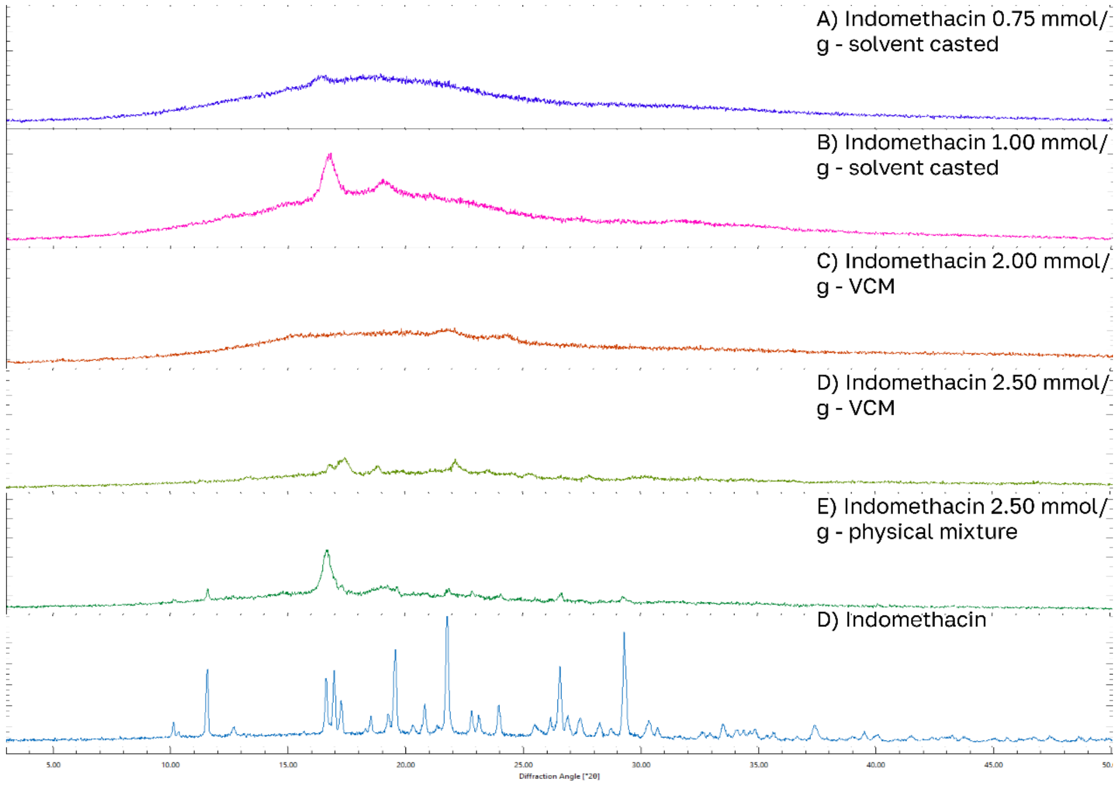

Figure S4. – XRD diffractograms of indomethacin samples.

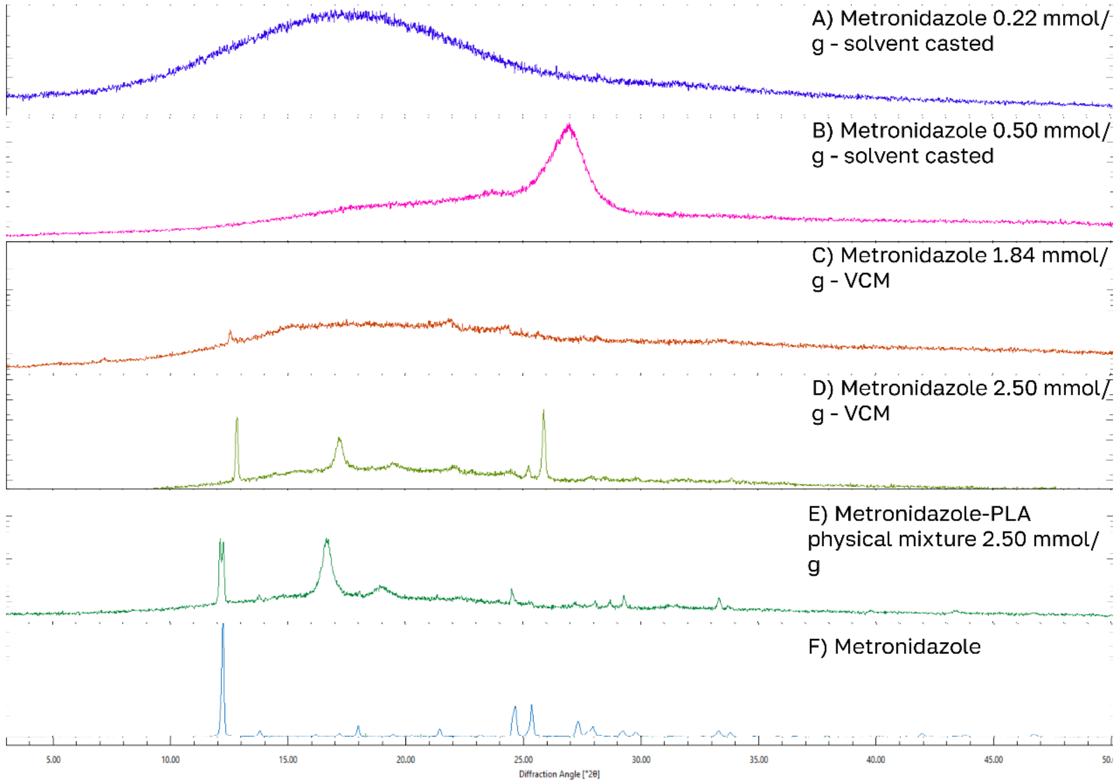

Figure S5. – XRD diffractograms of metronidazole samples.

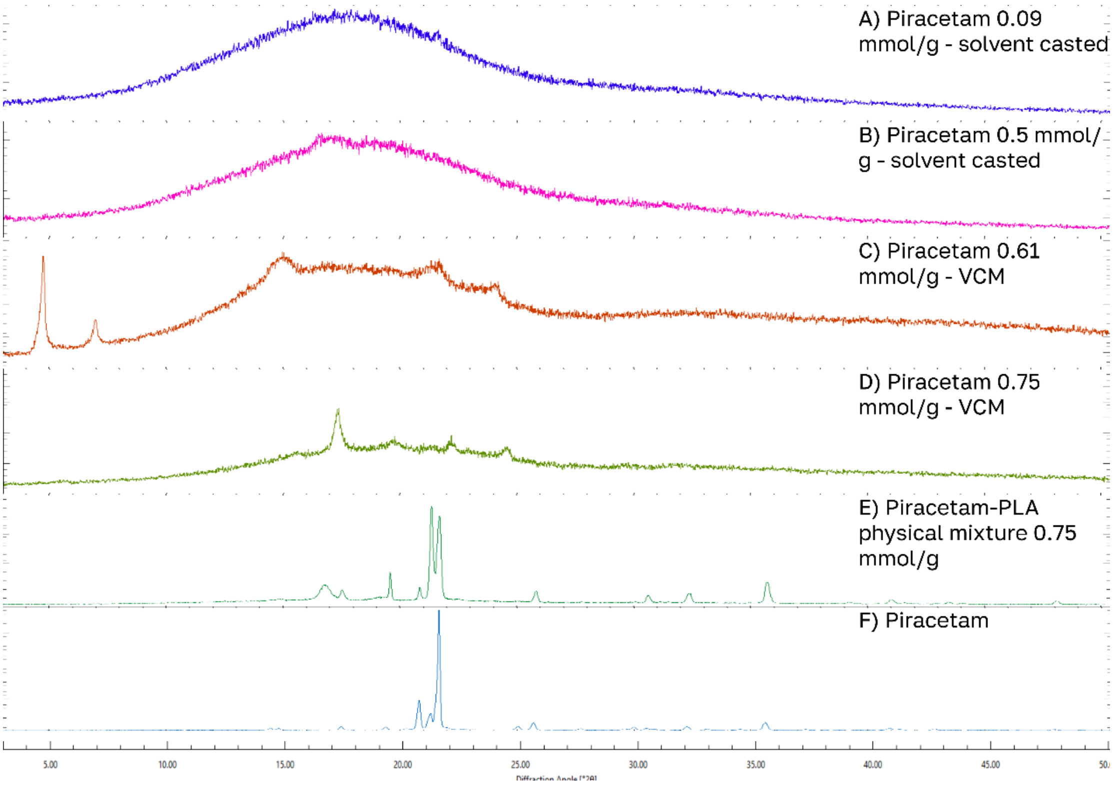

Figure S6. – XRD diffractograms of piracetam samples.

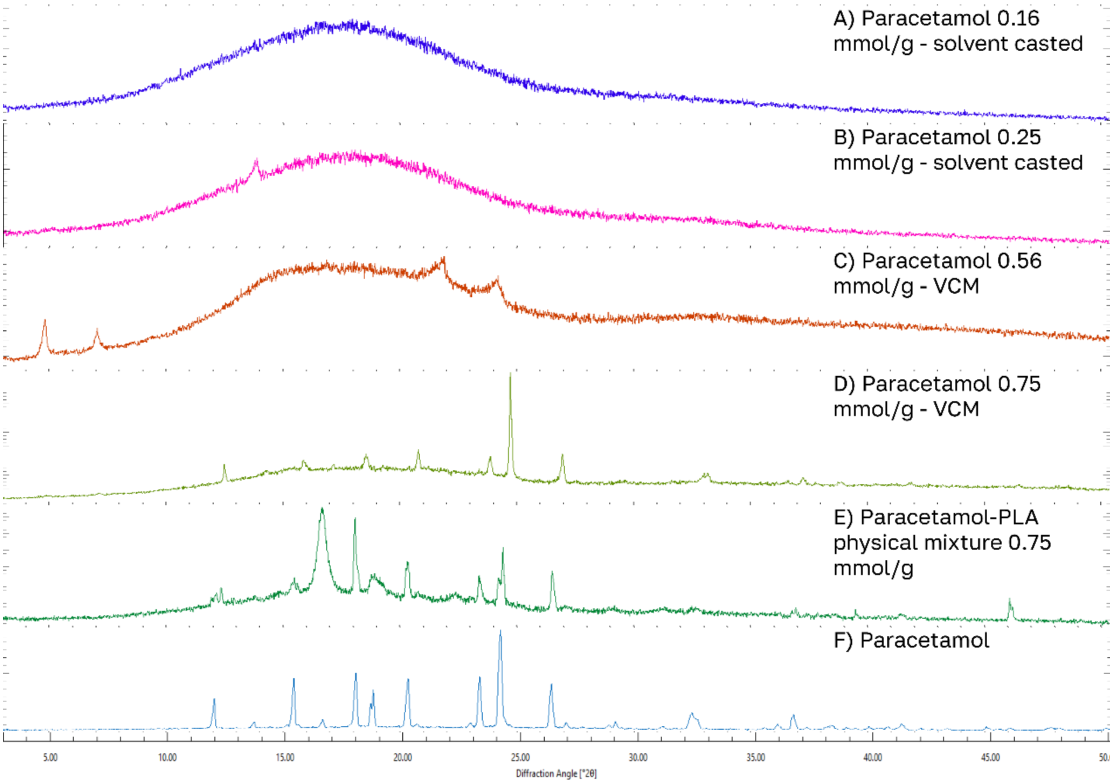

Figure S7. – XRD diffractograms of paracetamol samples.

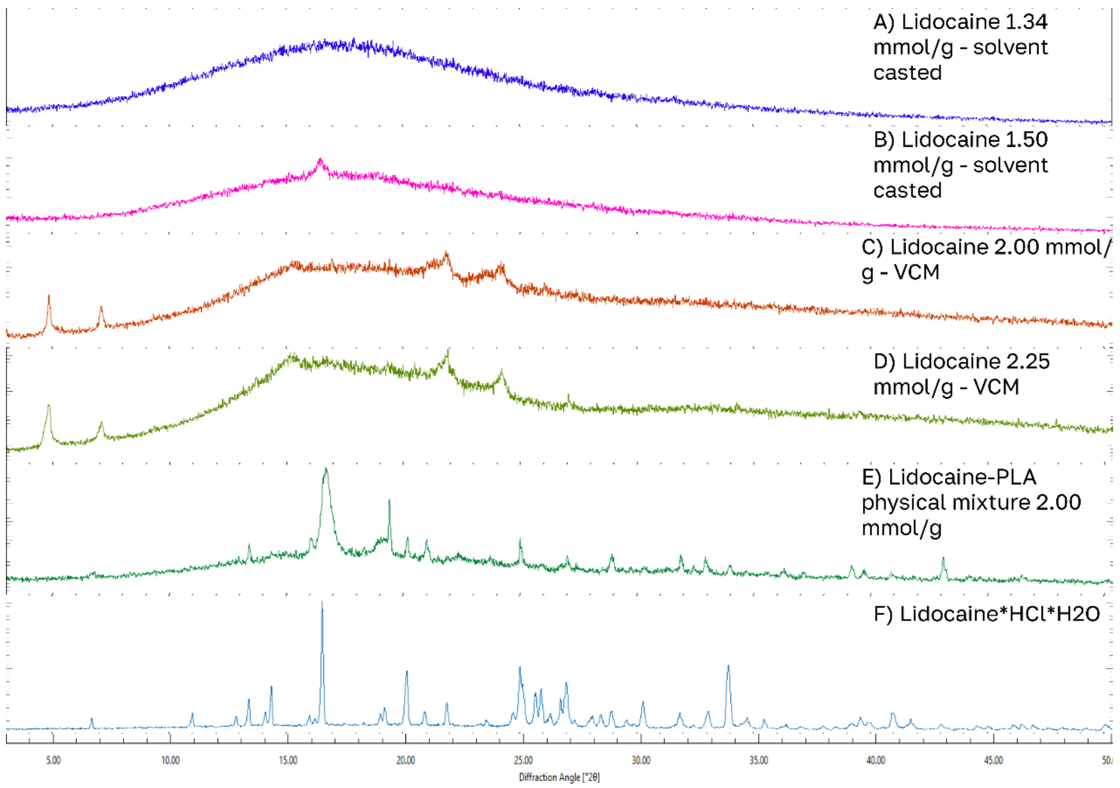

Figure S8. – XRD diffractograms of lidocaine samples.

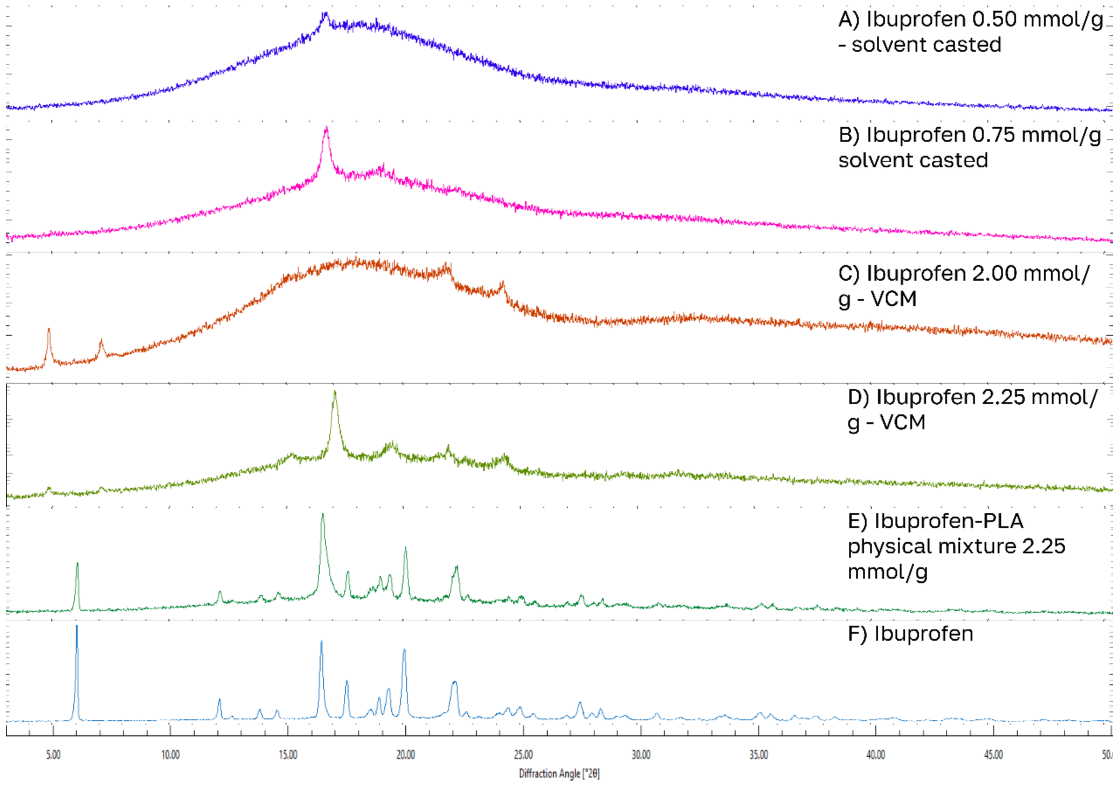

Figure S9. – XRD diffractograms of ibuprofen samples.

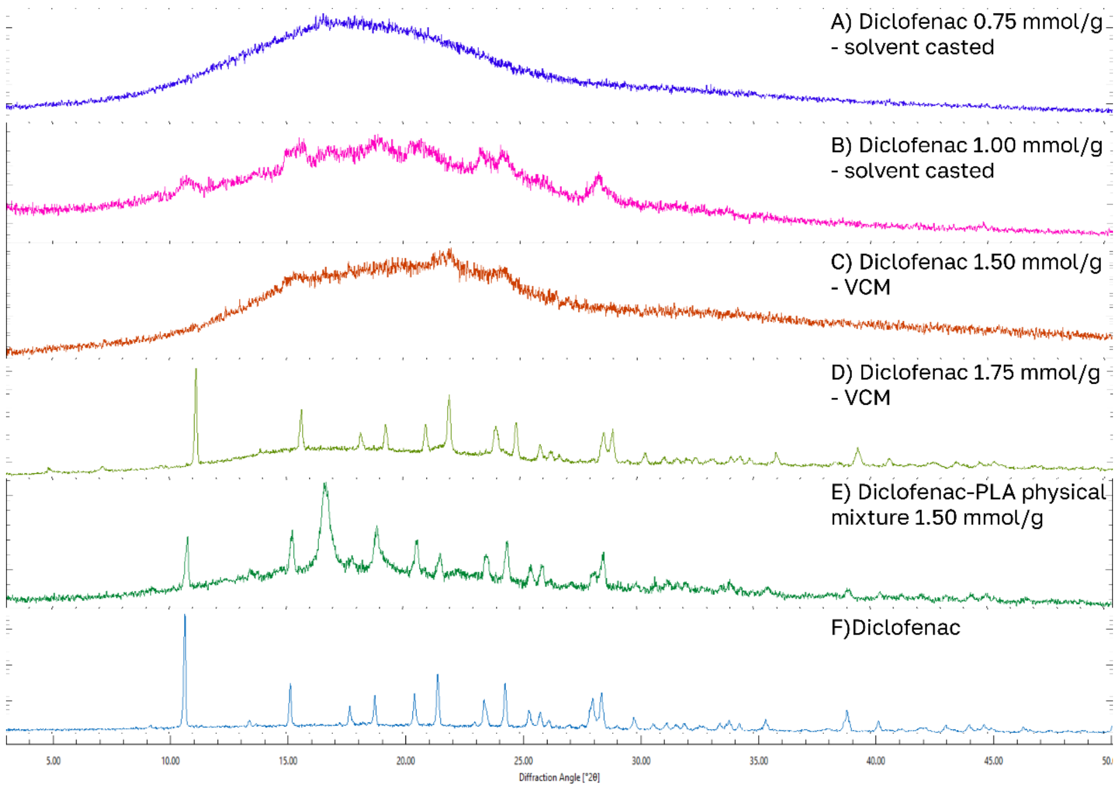

Figure S10. – XRD diffractograms of diclofenac samples.

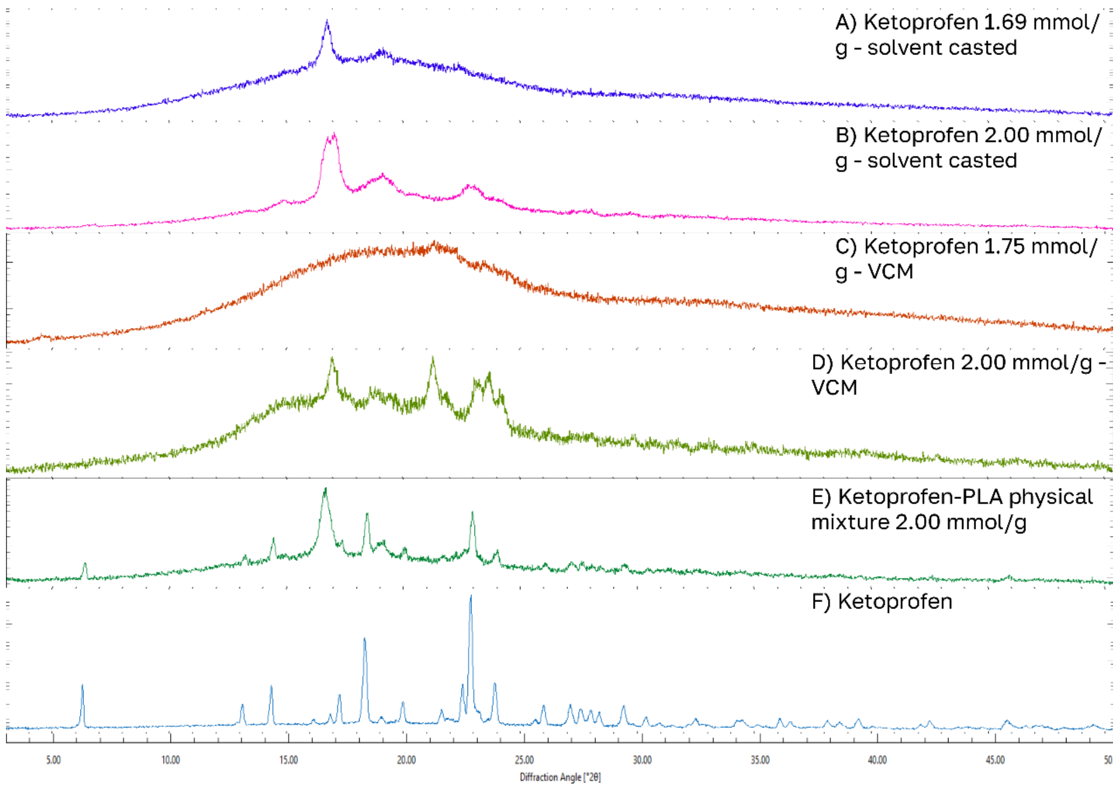

Figure S11. – XRD diffractograms of ketoprofen samples.

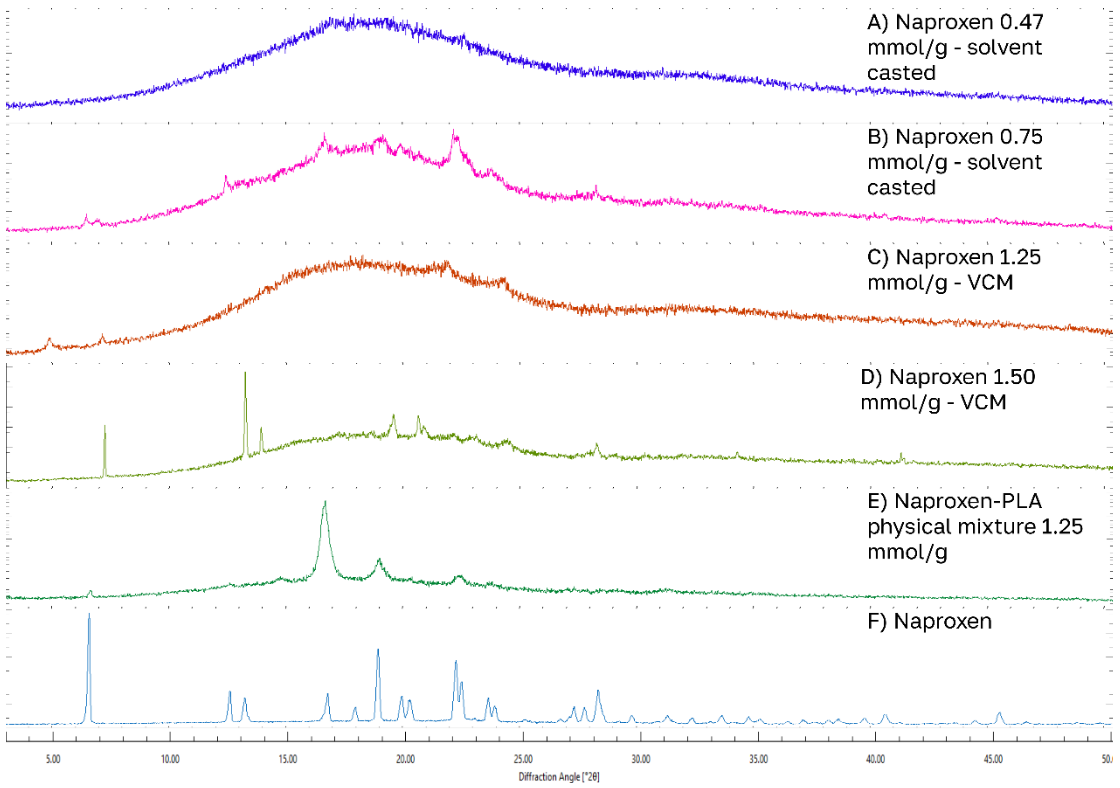

Figure S12. – XRD diffractograms of naproxen samples.

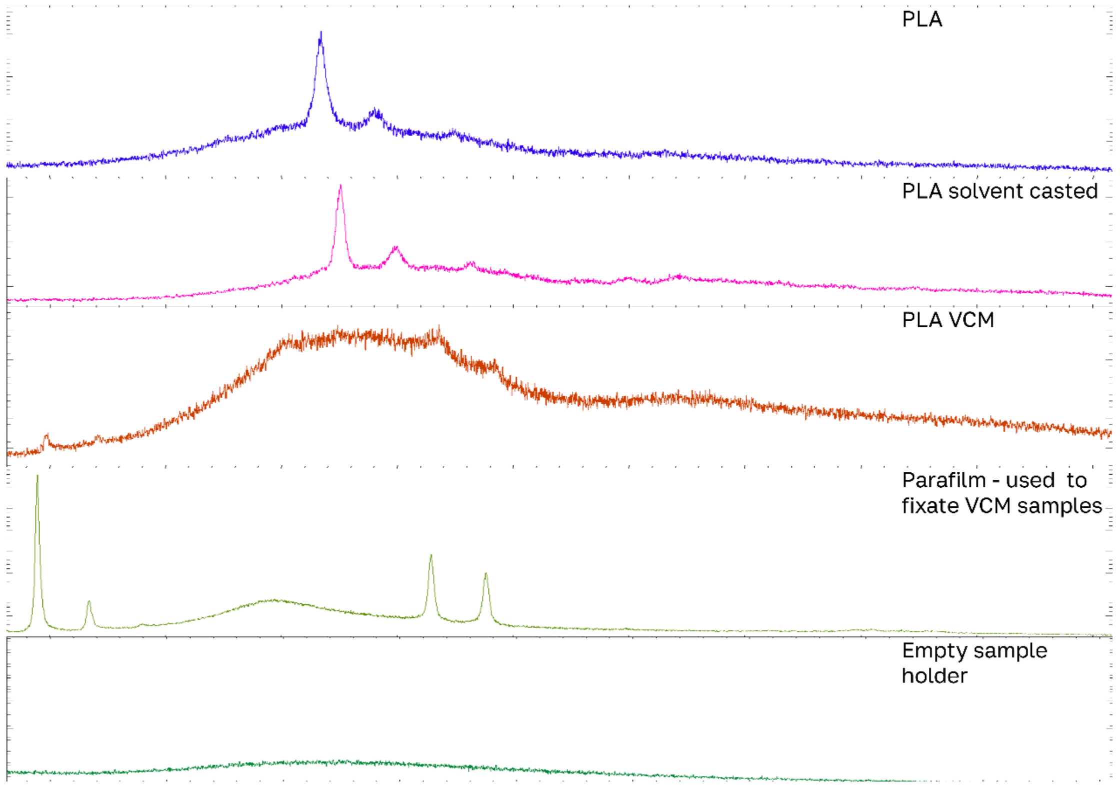

Figure S13. – XRD diffractograms of naproxen samples.

Table 1. – XRD peak list of amorphous saturated and drug crystaline samples (oversaturated samples).

Model drug	Drug loading method	Amorphous drug sample XRD peaks (Difr. angle 2Θ)	Crystaline drug sample XRD peaks (Difr. angle 2Θ)	Physical mixture XRD peaks (Difr. angle 2Θ)	Pure drug XRD peaks (Difr. angle 2Θ)

Benzocaine	SC	None	8.54084<br>17.16435<br>18.94221<br>20.16718<br>22.52552<br>23.31857<br>23.97684<br>27.23323<br>34.09908	11,5907<br>16,64261<br>18,7681<br>19,15553<br>20,1636<br>21,4542<br>21,8765<br>23,20253<br>23,86655<br>27,14455<br>34,45128	8.42831<br>11.5291<br>16.64074<br>16.94193<br>18.71571<br>20.0729<br>21.0297<br>21.53518<br>21.93932<br>23.16482<br>23.83011<br>25.50861<br>27.0995<br>27.50517<br>30.29184<br>32.80576<br>33.68825<br>34.37793<br>36.16838<br>36.91368<br>37.83225<br>39.16804<br>40.84427<br>43.88817<br>44.78074<br>45.88526<br>47.43743<br>48.03316
	VCM	4.95331<br>7.14309<br>78.32427	17.3821<br>17.65682<br>19.66343<br>20.83028<br>22.18358<br>24.53278<br>26.26703<br>27.82195		
Celecoxib	SC	None	16.98322<br>20.03869	5.37378<br>10.72314<br>13.00747<br>14.81375<br>16.12564<br>16.6574<br>17.89531<br>19.65931<br>21.34434<br>21.53482<br>22.1778<br>23.47435<br>25.37968<br>26.93892<br>29.54349<br>32.40663<br>34.93407<br>37.99754	5.31224<br>10.66409<br>10.97112<br>12.97826<br>14.79386<br>16.03719<br>17.33357<br>17.88681<br>19.62617<br>21.44228<br>22.12622<br>22.42039<br>23.42429<br>24.57444<br>24.97763<br>25.36624<br>26.90018<br>27.655<br>28.22671<br>29.54581<br>30.40168<br>31.42846<br>32.42212<br>34.89343<br>37.99576<br>39.40913<br>40.36705<br>41.05567<br>42.77889<br>43.46825<br>44.09416<br>44.58426<br>45.25723<br>45.97764
	VCM	None	4.79968<br>5.72517<br>7.14797<br>11.08219<br>13.37916<br>15.19031<br>16.45746<br>18.2675<br>19.99709<br>21.87548<br>22.5558<br>23.76872<br>24.21899<br>25.72511<br>27.3346<br>28.05008<br>29.95844<br>31.75544<br>32.85015<br>35.26947<br>38.43554<br>39.8259<br>40.74232<br>43.17962<br>43.88891<br>45.00578<br>45.59136<br>46.4647<br>49.91793		

					49.50314
Prednisolone	SC	None	16.67801<br>19.37362	15.50288<br>15.84754<br>16.63474<br>17.59415<br>18.98777<br>22.32586<br>25.25521<br>26.2117<br>31.80988	8.04133<br>10.52648<br>13.89797<br>15.48849<br>15.81547<br>16.23912<br>17.34322<br>17.62158<br>19.47755<br>21.21964<br>22.1315<br>22.65685<br>24.83464<br>25.23155<br>25.61421<br>26.20552<br>27.37707<br>27.81823<br>28.91853<br>31.12508<br>31.9413<br>32.51133<br>32.94609<br>34.85631<br>35.97893<br>36.92224<br>38.01167<br>39.00262<br>39.54055<br>40.64598<br>41.68093<br>42.73235<br>45.28262<br>45.68045<br>48.28395
	VCM	N/A	N/A		
Indomethacin	SC	None	16.94088<br>20.0081	11.60388<br>16.64583<br>19.20816<br>21.83751<br>22.83632<br>24.08175<br>26.66362<br>29.2557	10.182<br>11.60898<br>dec.33<br>16.67678<br>17.00525<br>17.29957<br>18.55913<br>19.31256<br>19.60439<br>20.35986<br>20.86338<br>21.80295<br>22.85281<br>23.16488<br>24.00091<br>25.56636<br>26.20301<br>26.59638<br>26.92187<br>27.45638<br>28.31082<br>28.78025<br>29.34555<br>30.38432<br>30.75805<br>31.64107
	VCM	None	13.50766<br>17.26045<br>18.88658<br>22.18482<br>24.62509<br>28.36809<br>55.18638		

					32.67138<br>33.53487<br>34.91531<br>35.69065<br>36.69776<br>37.44953<br>39.05477<br>39.55099<br>40.07208<br>41.5617<br>45.04485<br>45.62835<br>46.71797<br>47.47532<br>48.68869
Metronidazole	SC	None	27,20985	12,1424<br>12,27296<br>16,85151<br>19,08413<br>24,62247<br>28,02454<br>29,40505<br>33,38562<br>43,51453	12,2684<br>13,82113<br>16,19184<br>17,20914<br>17,99434<br>19,47476<br>20,26723<br>20,64811<br>21,4544<br>23,17614<br>24,01115<br>24,65402<br>25,35256<br>27,34626<br>27,96383<br>29,21586<br>29,77933<br>31,2514<br>33,2961<br>33,79682<br>34,8102<br>35,80796<br>36,52085<br>37,90324<br>38,42348<br>39,0274<br>40,19472<br>41,2693<br>41,92294<br>42,30453<br>43,31786<br>43,7503<br>46,06918<br>46,67441<br>49,07005<br>49,98785
	VCM	14,2992<br>19,76101<br>22,88176	12,83522<br>17,18614<br>19,47881<br>22,17947<br>25,25581<br>25,90744<br>29,86327		
Piracetam	SC	None	4.78623<br>6.98921	14.87879<br>16.78957<br>17.53488<br>19.21448<br>19.6019<br>20.88672<br>21.37419<br>21.69475<br>22.49851<br>23.35939<br>25.8452	10.81116<br>14.47875<br>14.83191<br>17.49766<br>19.39377<br>20.28138<br>20.83423<br>21.31204<br>21.68627<br>22.51232<br>25.06117
	VCM	4,78623<br>6,98921	17.49459<br>19.67714<br>24.62599<br>33.248		

				27.77201<br>30.63047<br>32.3768<br>35.70873<br>39.22259<br>41.0408<br>41.592<br>42.98434<br>43.48211<br>48.1156	25.70728<br>27.72377<br>28.84188<br>29.62878<br>30.01558<br>30.55463<br>30.90216<br>31.48126<br>32.26989<br>33.13107<br>34.00706<br>34.51676<br>35.02444<br>35.62452<br>37.3433<br>37.85823<br>38.43575<br>39.18844<br>40.39699<br>40.93405<br>41.3731<br>42.33954<br>42.86335<br>43.38103<br>44.12149<br>44.80506<br>47.97684<br>49.11429
Paracetamol	SC	None	17.9418	15.43465<br>16.67366<br>18.07849<br>18.88825<br>20.34384<br>22.39266<br>23.40753<br>24.23833<br>24.41401<br>26.53503<br>29.1074<br>41.435<br>46.1089	12.07605<br>13.77558<br>15.47508<br>16.70521<br>18.12815<br>18.74739<br>18.8676<br>20.34827<br>20.73468<br>23.02922<br>23.41693<br>24.29777<br>26.47435<br>27.11465<br>28.93965<br>29.22913<br>31.22984<br>32.47011<br>35.59195<br>36.14768<br>36.79804<br>37.40407<br>38.46187<br>39.11577<br>40.03737<br>40.86325<br>41.4626<br>42.25799<br>43.40358<br>45.07654<br>46.09035<br>47.83323
	VCM	4,85001<br>7,06379<br>21,84605<br>24,19174<br>37,31295<br>74,71857	4.99612<br>12.49798<br>15.93459<br>18.57123<br>20.8005<br>23.88007<br>24.74767<br>26.96463<br>31.68273<br>33.20672<br>37.25126<br>38.86258		
Lidocaine	SC	None	16.51411	6.78617	6.71455
	VCM	4,86084	4.99733	13.41009	11.01503

		7,08781	7.05668	16.6656	12.88683
		17,8438	20.41529	19.0805	13.43197
			24.25663	19.40329	14.13154
			59.2408	20.18558	14.38657
				20.99502	16.03095
				25.01155	16.56987
				27.00753	19.04929
				28.92772	19.23658
				31.88656	20.18121
				32.92398	20.94389
				33.96618	21.90085
				37.1329	23.30428
				39.19137	23.58029
				39.71597	24.70326
				40.87011	25.02812
				43.101	25.68171
				46.45379	25.92575
					26.31823
					26.7654
					27.0114
					28.09079
					28.49013
					28.93945
					29.55776
					30.28387
					31.85384
					32.45608
					33.07197
					33.93294
					34.69529
					35.45919
					37.05735
					38.00808
					39.59147
					39.97257
					40.96115
					41.73459
					43.03197
					44.59768
					45.03945
					46.13023
					46.49653
					46.96333
					49.24198
Ibuprofen	SC	None	17.80414	6.08861	6.07444
	VCM	4,8838	4.81723	12.17641	12.18664
		7,11903	17.04322	13.94137	12.70878
		22,0126	19.51565	14.6671	13.89875
		24,30388		16.55796	14.64043
				17.63246	16.55103
				19.03489	17.61801
				19.44521	18.65697
				20.10858	19.00934
				22.29888	19.41579
				22.77456	20.07749
				25.11616	22.24266
				25.66303	22.73604
				27.62951	24.49222
				29.40146	25.02153
				30.9111	25.60591
				33.80427	27.03729
				37.69005	27.59916

				43.42219<br>43.83985	28.1218<br>28.49214<br>29.51311<br>30.89585<br>31.8875<br>32.66804<br>35.28006<br>35.75208<br>36.75408<br>37.67745<br>38.47978<br>39.46343<br>40.99564<br>42.42903<br>43.59907<br>44.92077<br>47.41485
Diclofenac	SC	None	23.90932<br>19.38115<br>28.37957	10.77815<br>15.26397<br>16.67645<br>18.905<br>20.59909<br>21.61295<br>23.5711<br>24.47926<br>25.49264<br>25.97125<br>28.17663<br>28.58462<br>35.61406<br>39.06824<br>44.32317	10.69076<br>13.45296<br>15.20526<br>17.73853<br>18.81331<br>20.51404<br>21.50396<br>23.52131<br>24.38913<br>25.41307<br>25.89298<br>26.25388<br>28.12248<br>28.51529<br>29.89988<br>30.72524<br>31.26865<br>32.03408<br>32.74723<br>33.58637<br>33.96685<br>34.41082<br>35.52471<br>38.2291<br>39.02278<br>40.3573<br>41.35807<br>42.31914<br>43.2251<br>44.22026<br>44.8617<br>46.56633<br>47.7333<br>49.08501
	VCM	17,15214<br>21,89735	4.88965<br>7.1873<br>11.16339<br>15.66245<br>18.19112<br>19.26627<br>20.97917<br>21.98113<br>23.99323<br>24.83737<br>25.86903<br>26.318<br>28.5945<br>28.96153<br>30.35088<br>31.1608<br>32.5617<br>33.27386<br>34.41122<br>34.86238<br>35.97902<br>38.47496<br>39.462<br>40.79052<br>41.73548<br>42.64012<br>43.71678<br>44.70866<br>45.31576<br>46.99588		
Naproxen	SC	None	6,48594<br>6,98561<br>12,63711<br>16,71729<br>19,37543<br>22,26245	6.67584<br>14.78913<br>16.66001<br>18.9912<br>22.42299	6.61625<br>12.63237<br>13.28578<br>16.82398<br>17.99372<br>18.96558<br>19.97366<br>20.33205<br>22.29725<br>22.5452<br>23.67188
	VCM	4,97409<br>7,17917<br>18,01224	7.24946<br>13.25855<br>13.93237<br>19.61022<br>20.65824		

<table>
<tr><th></th><th></th><th></th><th></th><th></th><th></th></tr>
<tr><td></td><td></td><td></td><td>22.13533
24.4464
28.29988
31.95556
41.30447</td><td></td><td>23.94956
25.24727
26.78275
27.33417
27.80057
28.35763
29.81172
31.34432
32.38905
33.66291
34.81005
35.26632
36.47727
37.13823
37.60958
38.21471
38.63662
39.76375
40.64286
41.19624
43.67261
44.50244
45.52787
46.69389
48.47843
49.84703</td></tr>
<tr><td rowspan="2">Ketoprofen</td><td>SC</td><td>16.76728
19.10556</td><td>17.15045
19.04809
22.85686
31.82772</td><td rowspan="2">6,3955
13.26339
14.45931
16.65246
17.38409
18.45616
19.10389
20.07599
22.99194
24.02814
26.0921
27.21645
27.645
29.479</td><td rowspan="2">6.31409
13.13724
14.38088
16.1749
16.90754
17.2942
18.37805
19.08023
19.98535
21.64609
22.55024
22.8951
23.91393
26.00564
27.1464
27.60626
28.01126
28.3738
29.4436
30.38922
30.95302
32.53122
34.41321
35.16373
36.10543
36.54304
38.11425
38.63549
39.46735
42.51363
45.77619
48.26494
49.52</td></tr>
<tr><td>VCM</td><td>4,53462</td><td>17.10021
18.97956
21.28629
23.18609
24.11634</td></tr>
</table>

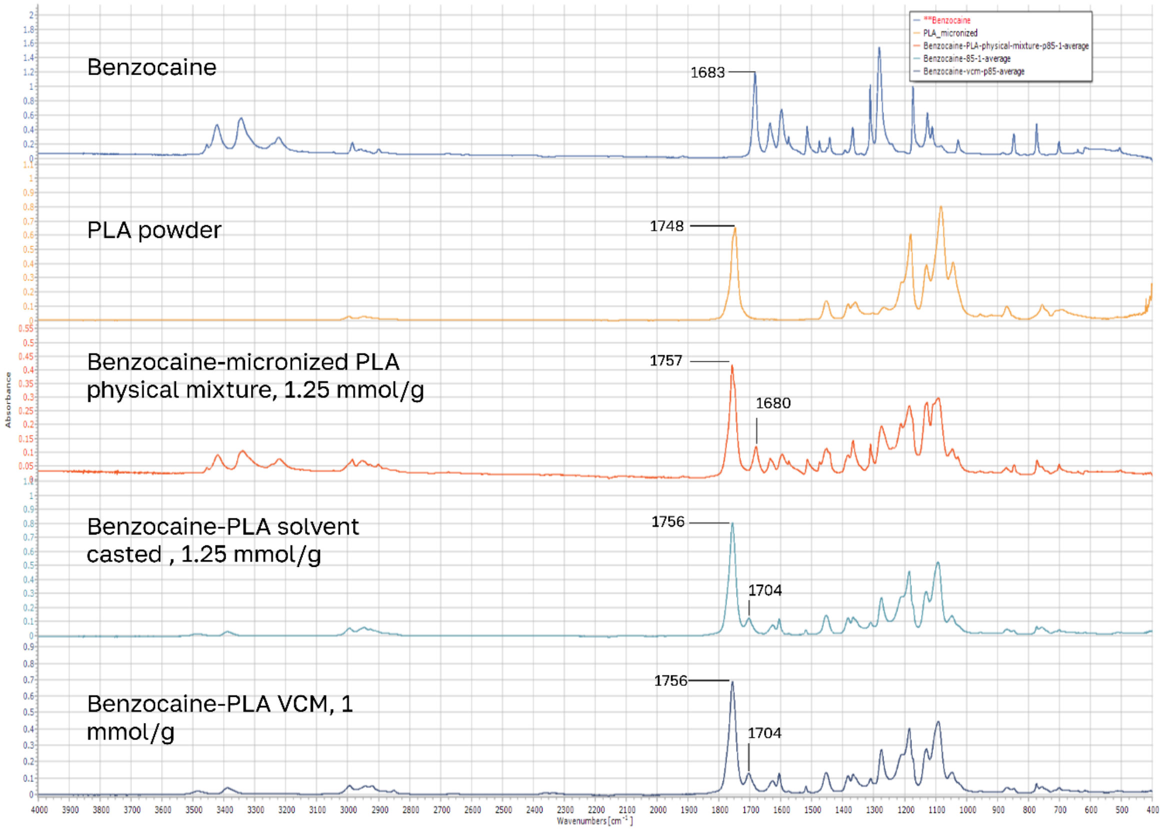

Figure S14. – FTIR-ATR analysis of benzocaine samples.

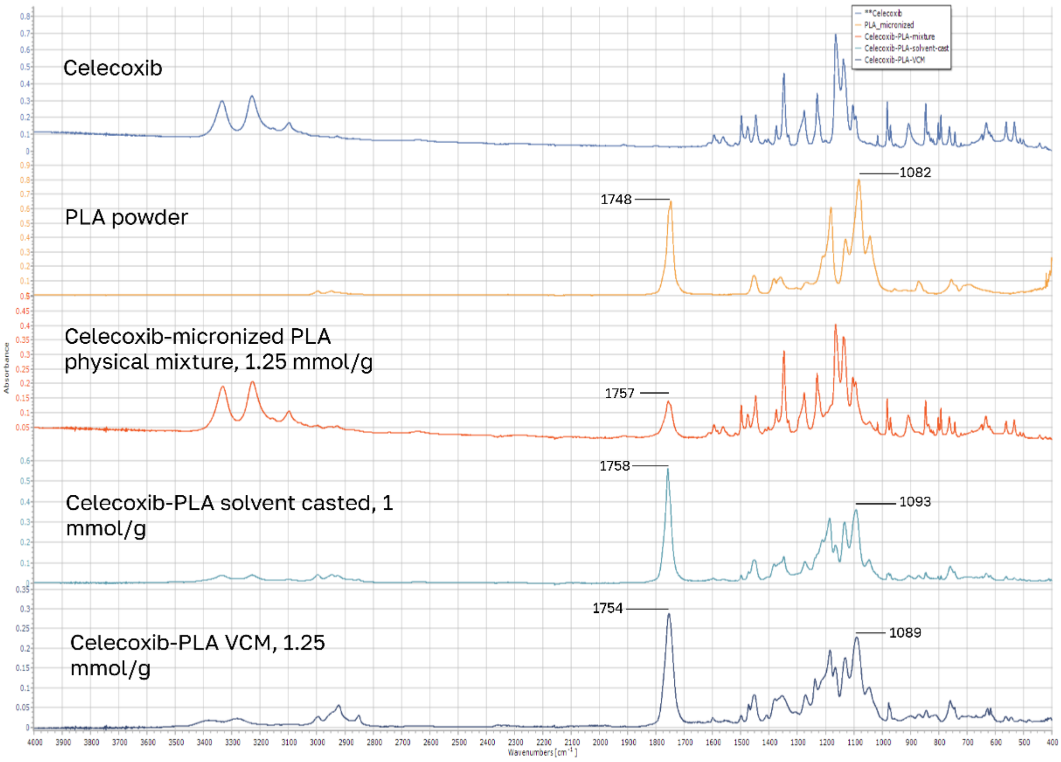

Figure S15. – FTIR-ATR analysis of celecoxib samples.

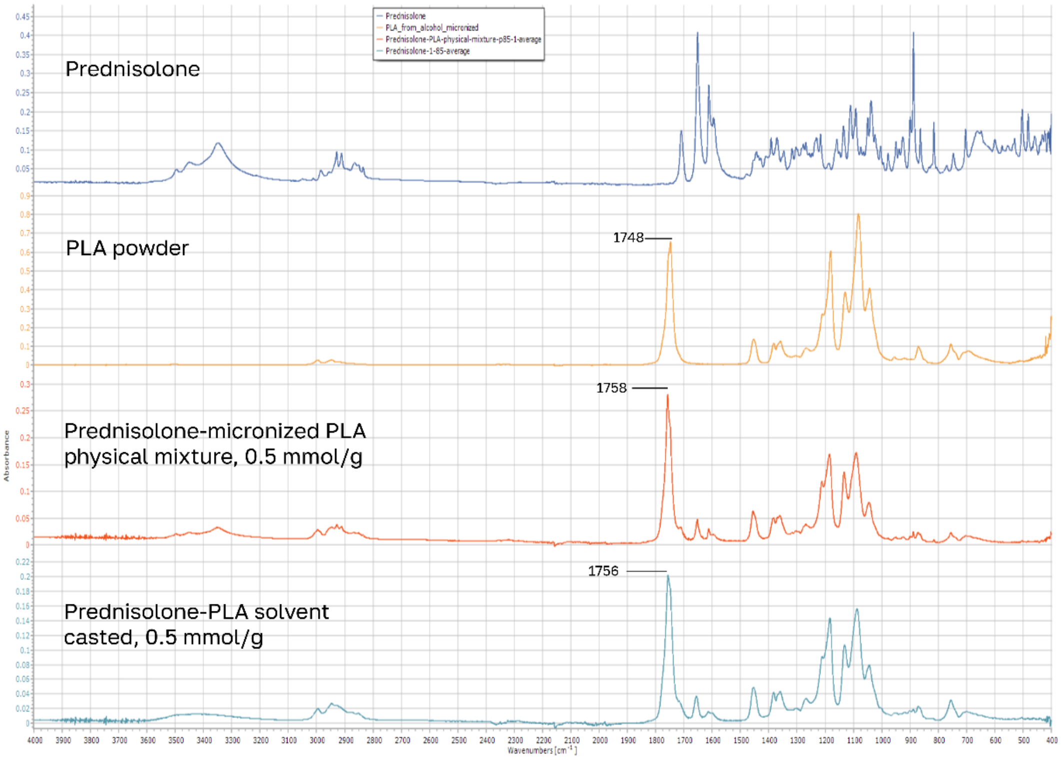

Figure S16. – FTIR-ATR analysis of prednisolone samples.

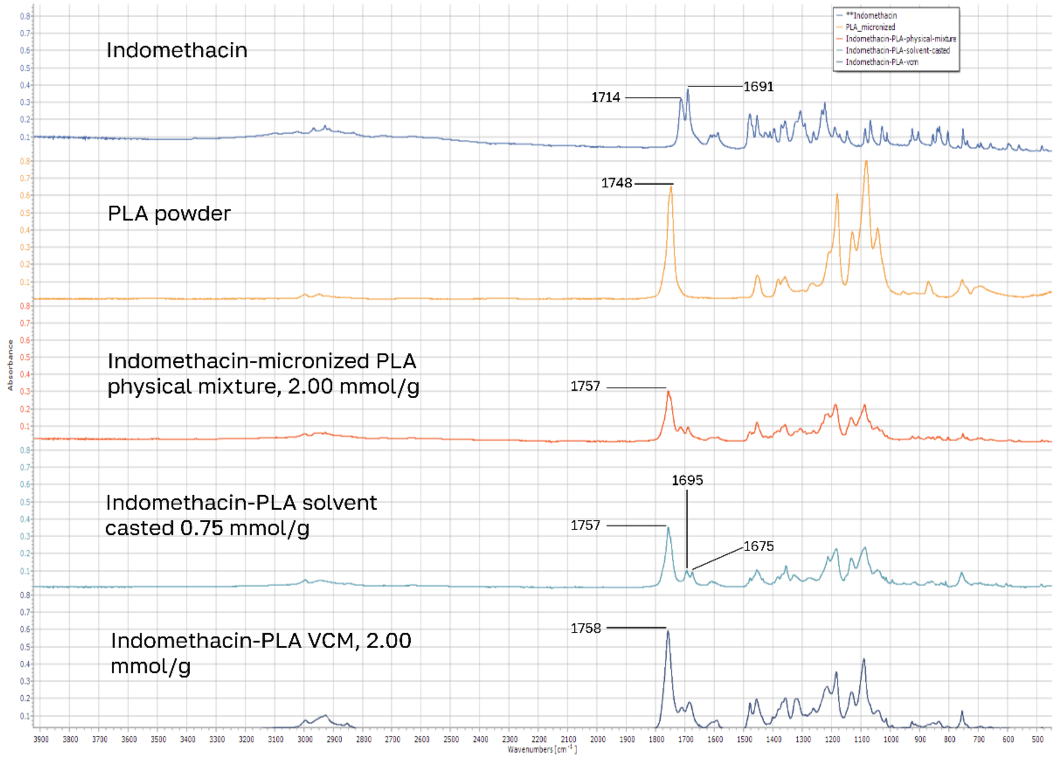

Figure S17. – FTIR-ATR analysis of indomethacin samples.

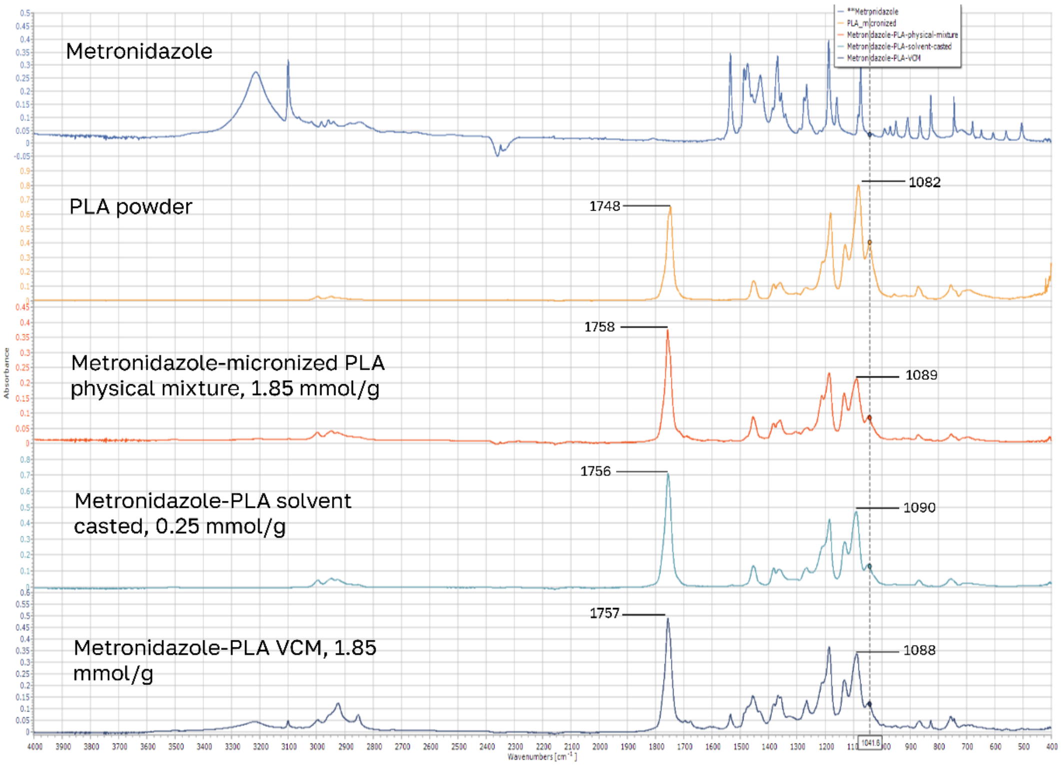

Figure S18. – FTIR-ATR analysis of metronidazole samples.

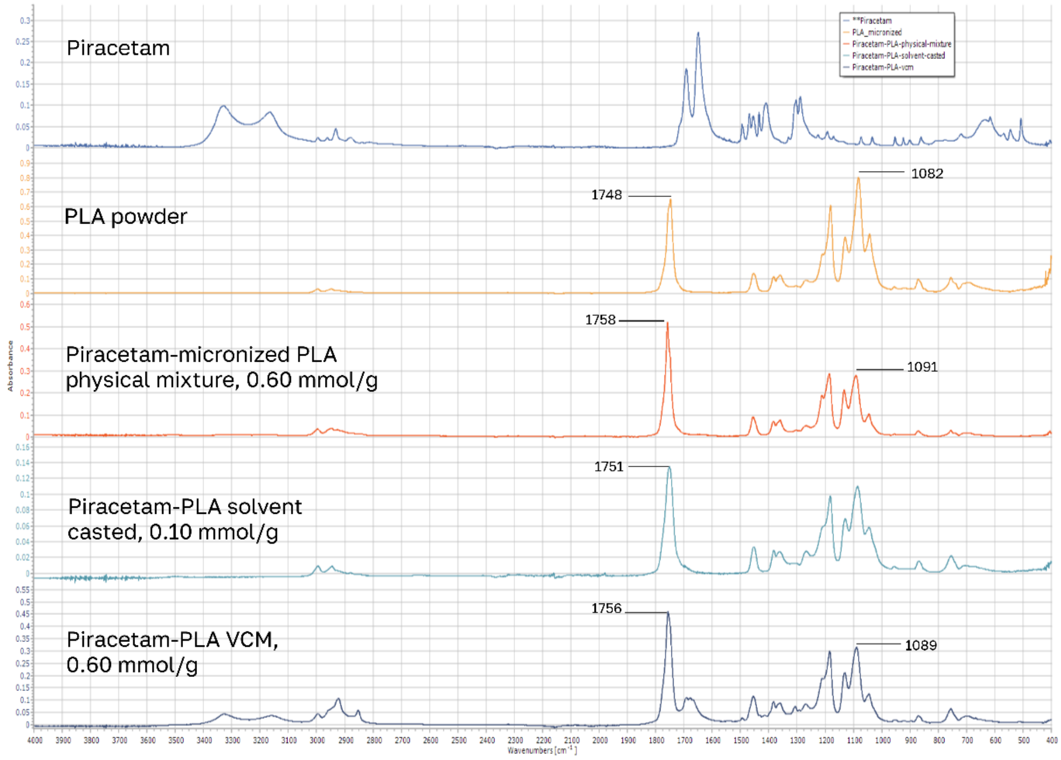

Figure S19. – FTIR-ATR analysis of piracetam samples.

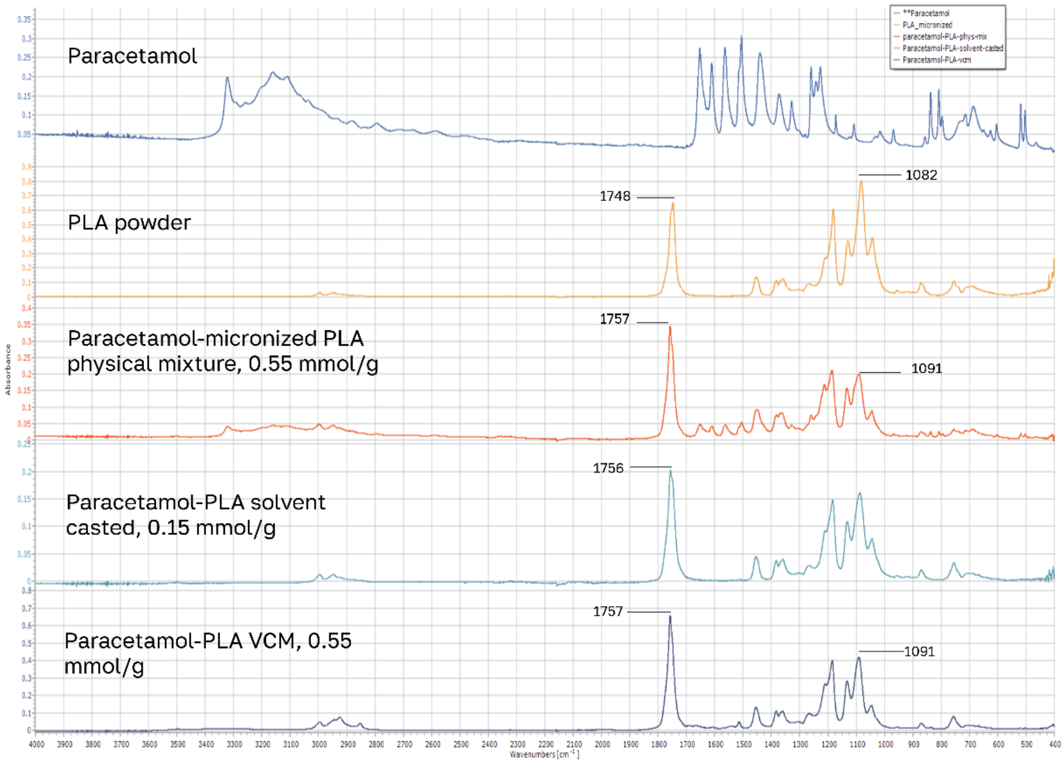

Figure S20. – FTIR-ATR analysis of paracetamol samples.

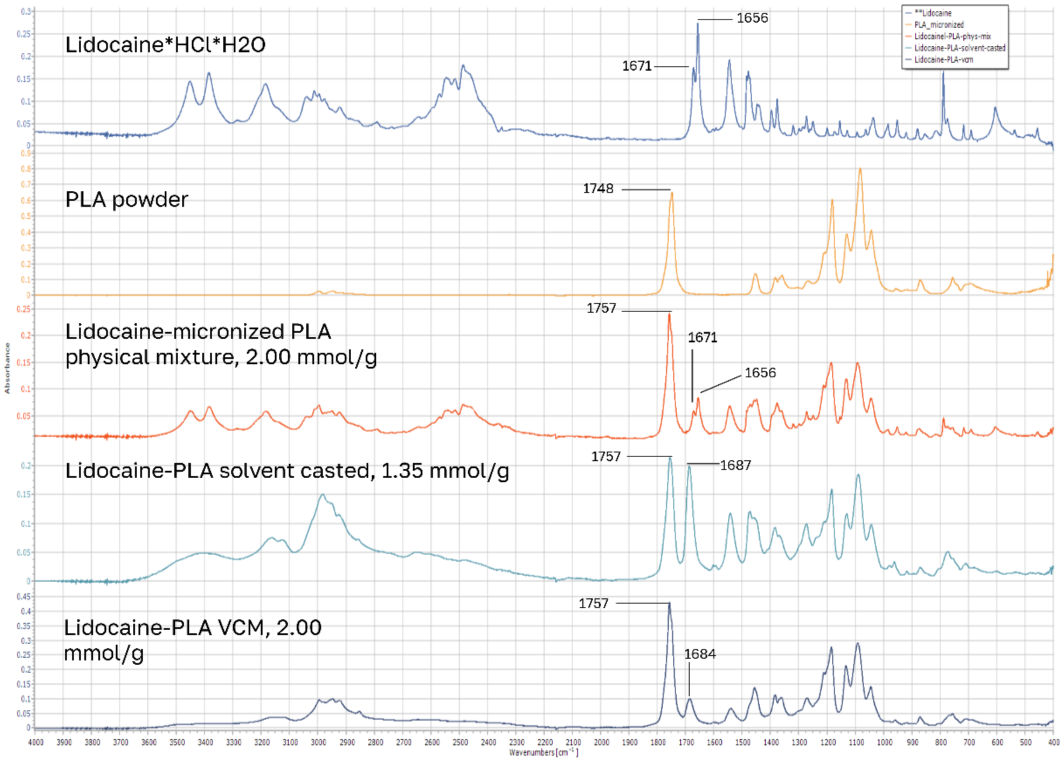

Figure S21. – FTIR-ATR analysis of lidocaine samples.

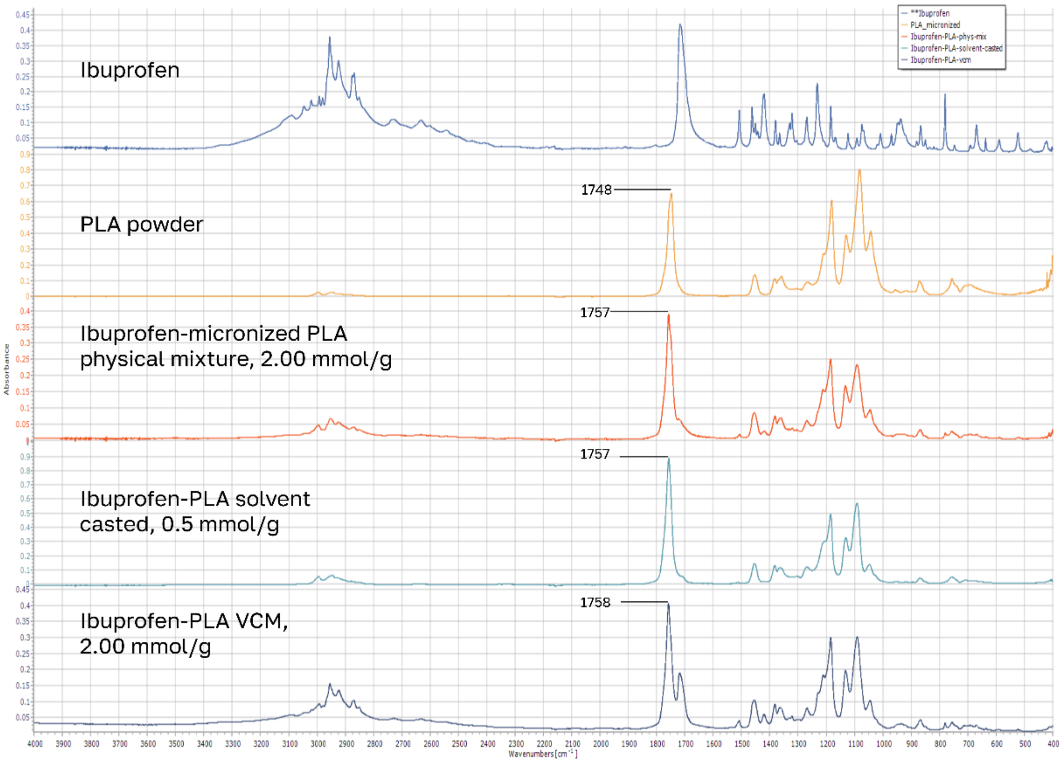

Figure S22. – FTIR-ATR analysis of ibuprofen samples.

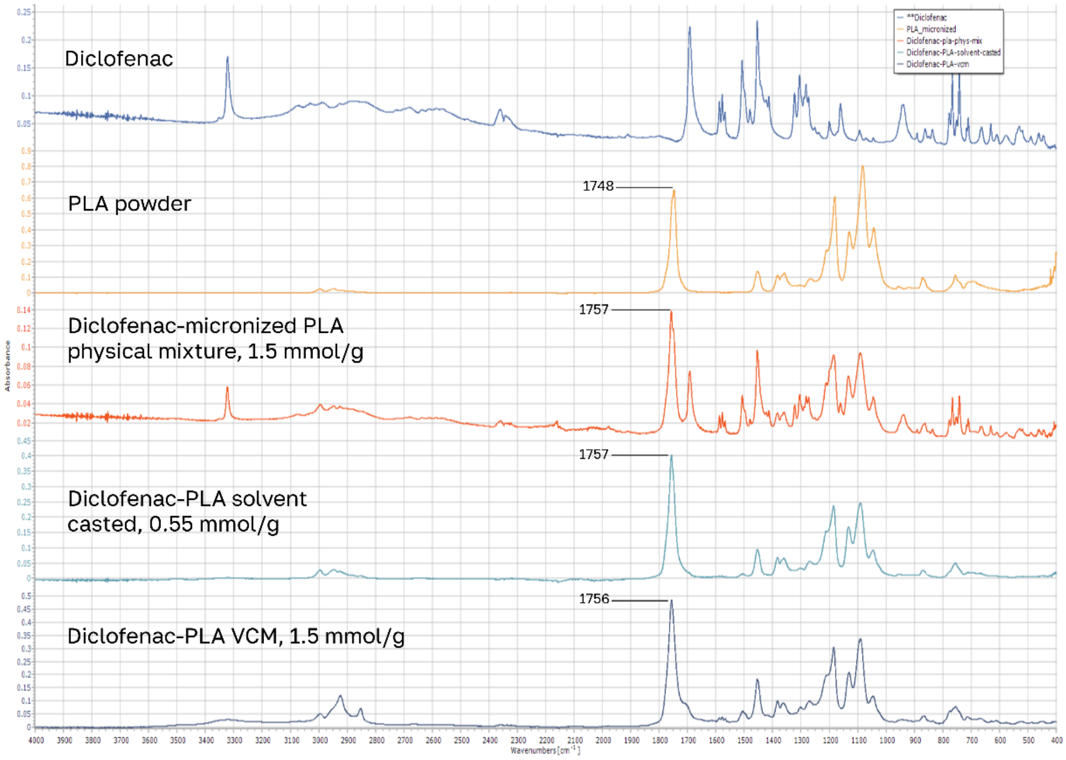

Figure S23. – FTIR-ATR analysis of diclofenac samples.

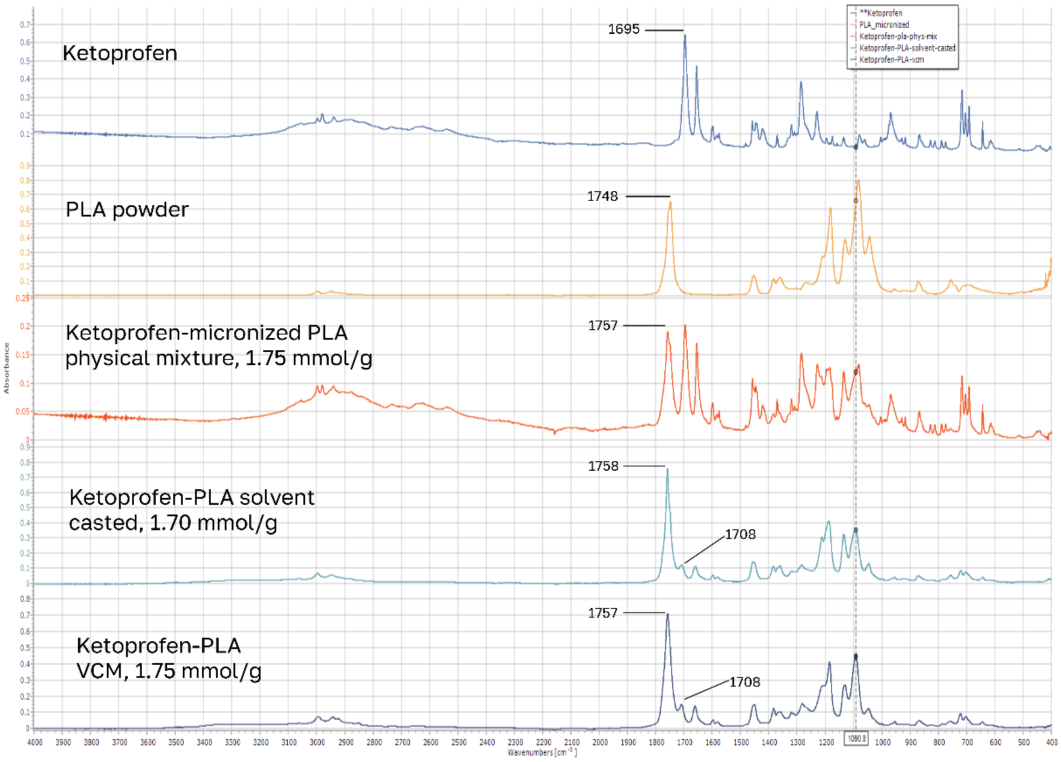

Figure S24. – FTIR-ATR analysis of ketoprofen samples.

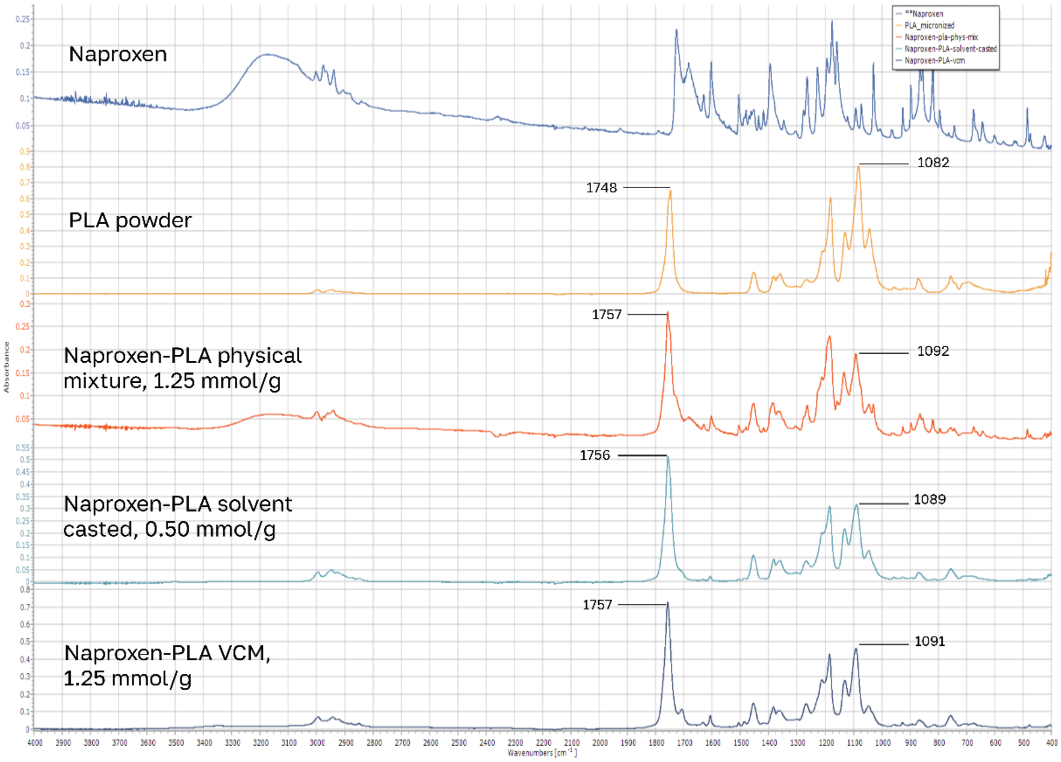

Figure S25. – FTIR-ATR analysis of naproxen samples.

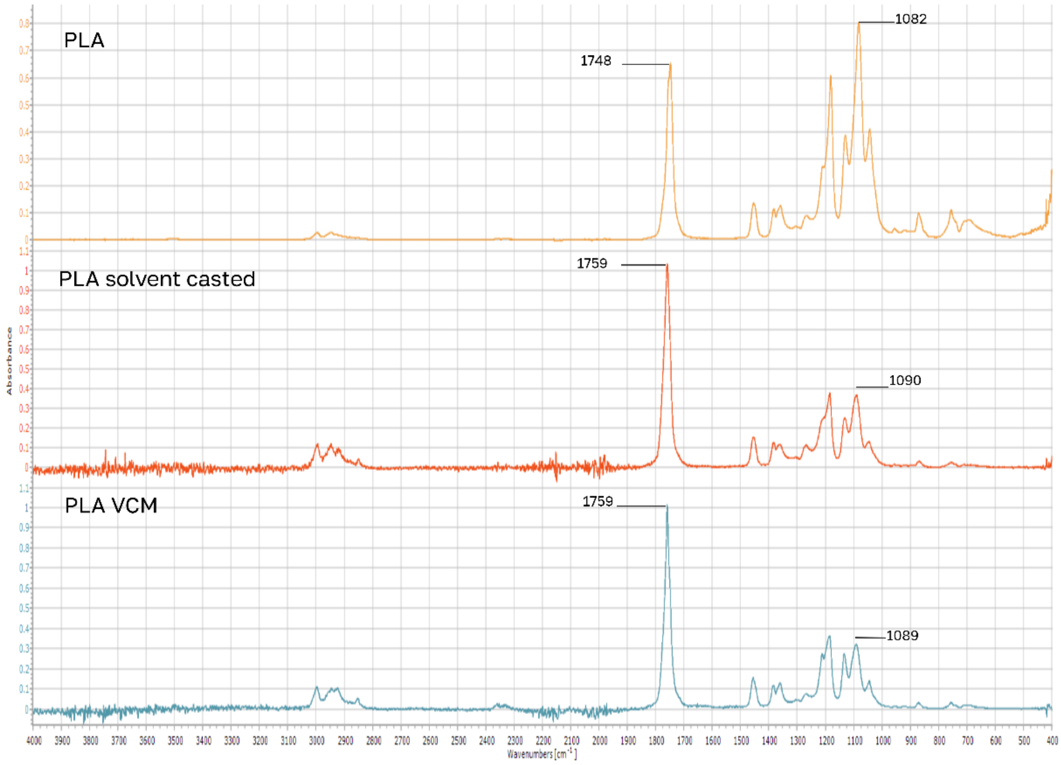

Figure S26. – FTIR-ATR analysis of blank PLA samples.

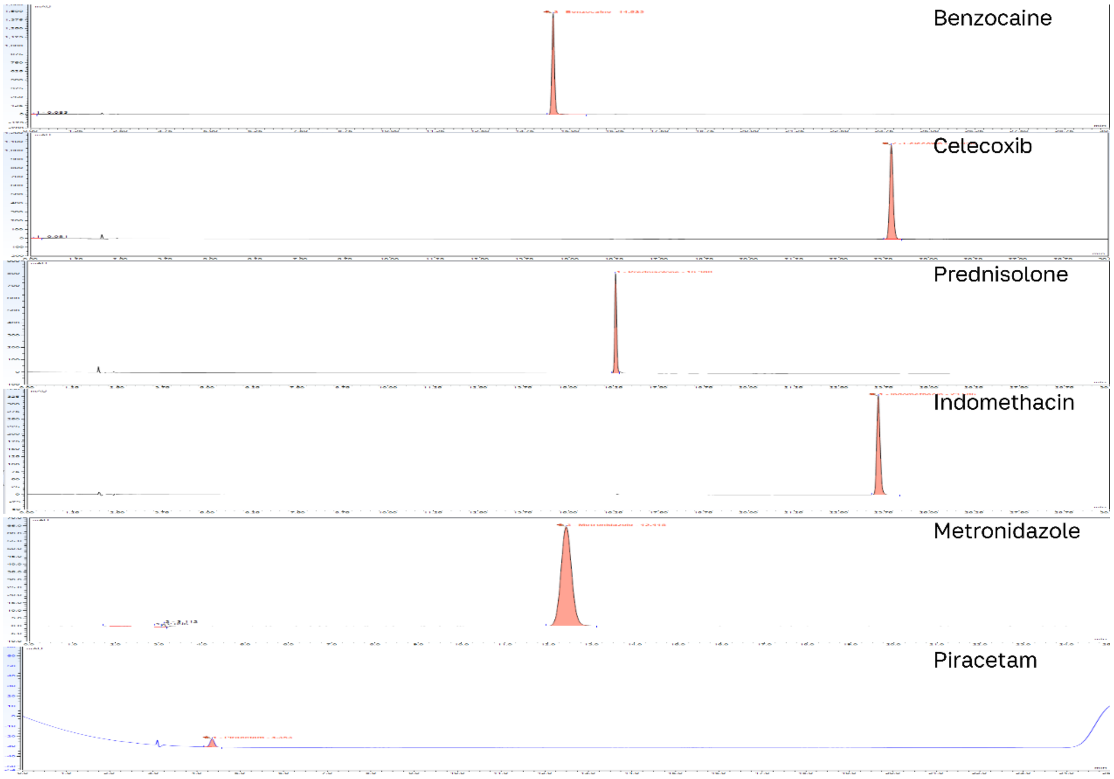

Figure S27. – Chromatogramms of saturated, amorphous solvent casted PLA samples.

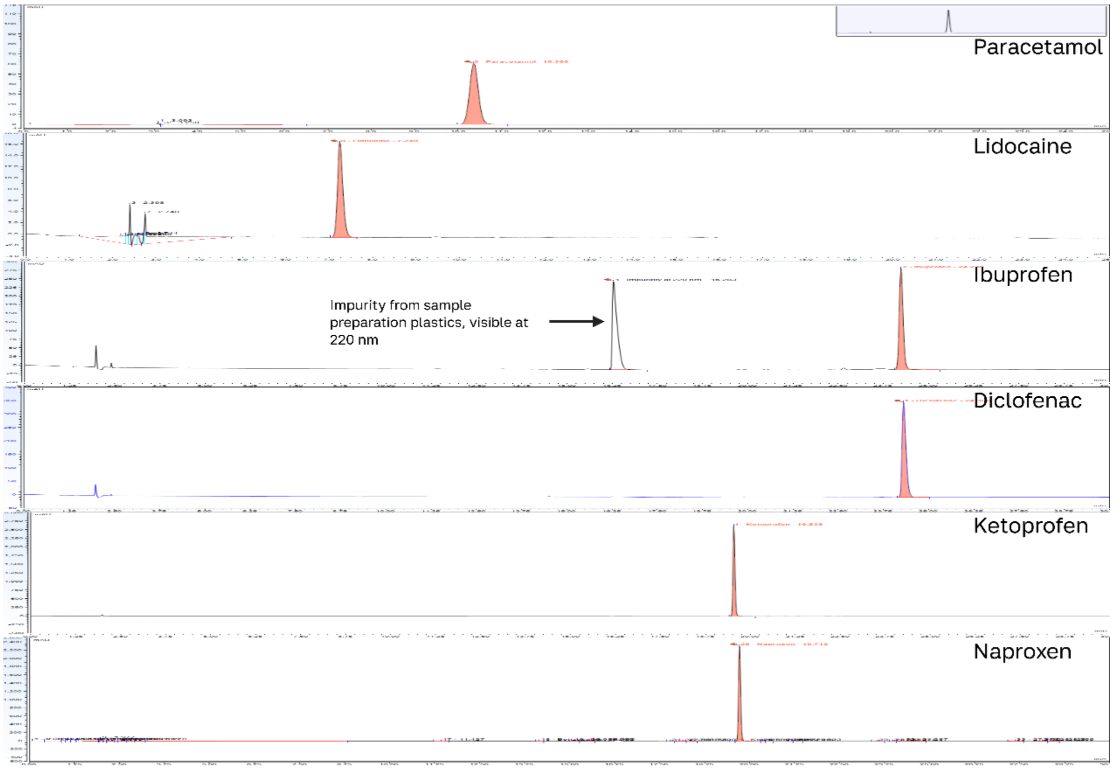

Figure S28. – Chromatogramms of saturated, amorphous solvent casted PLA samples.

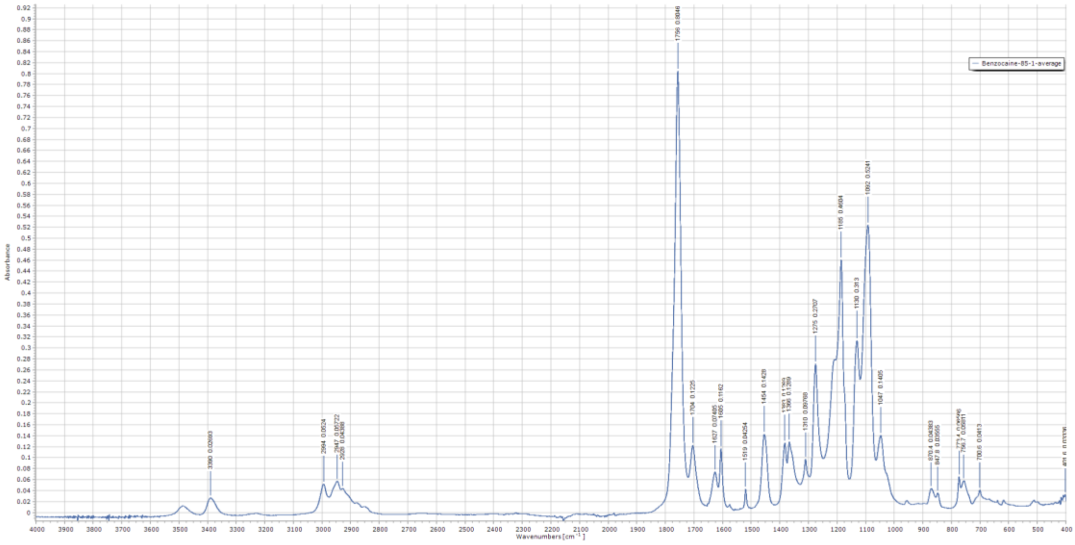

Figure S29. – Benzocaine-PLA solvent casted amorphous sample.

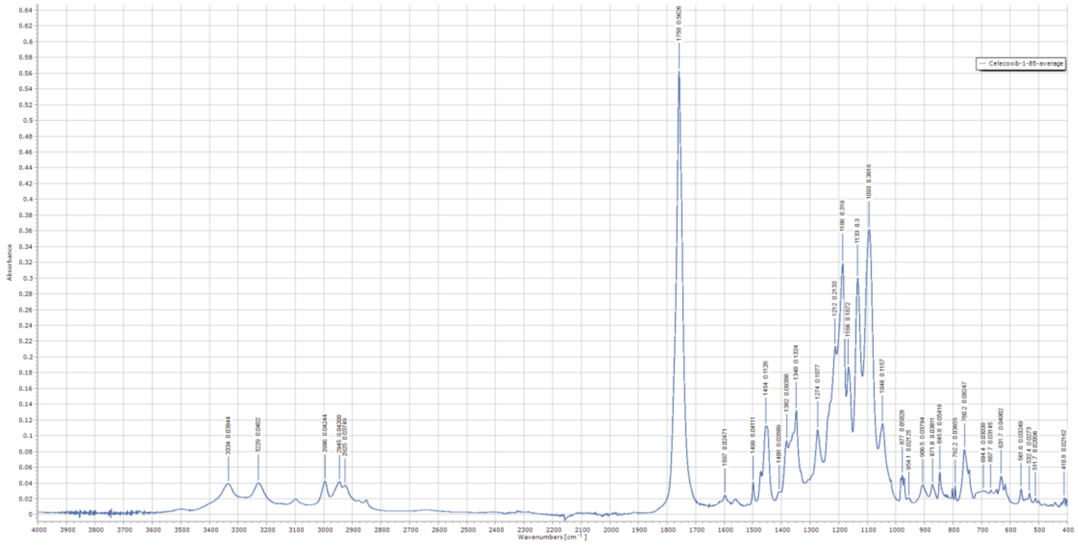

Figure S30. – Celecoxib-PLA solvent casted amorphous sample.

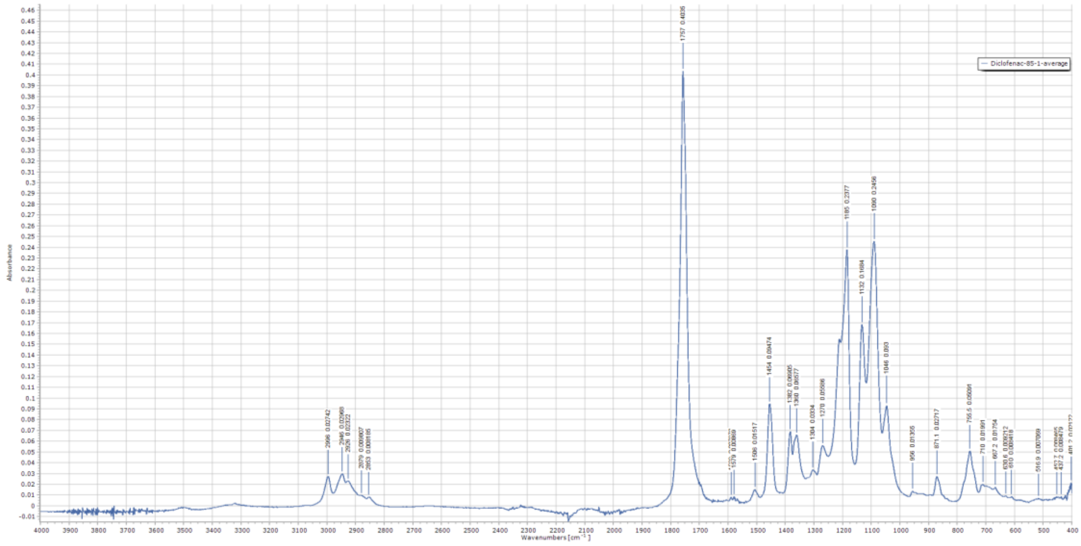

Figure S31. – Diclofenac-PLA solvent casted amorphous sample.

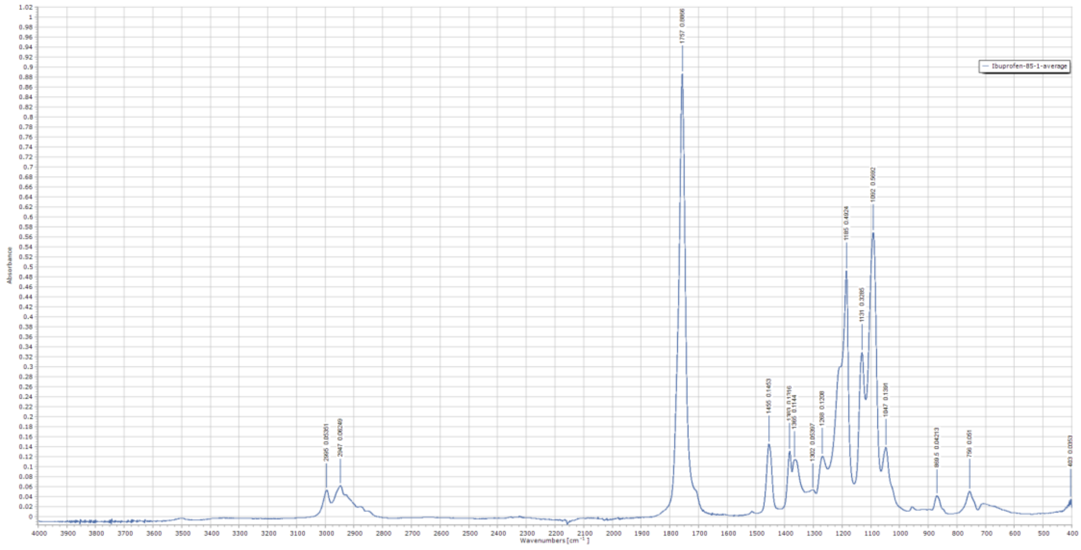

Figure S32. – Ibuprofen-PLA solvent casted amorphous sample.

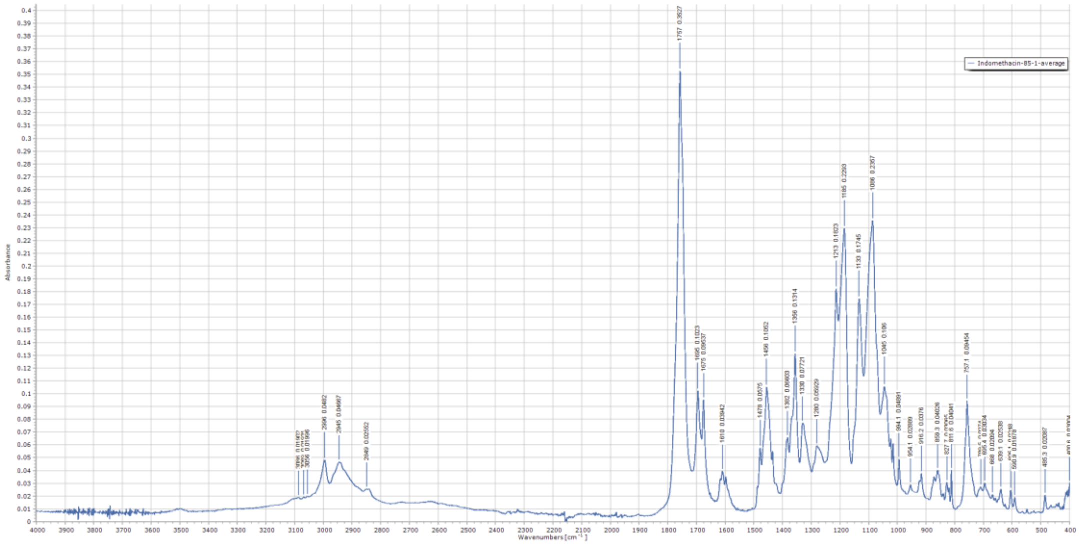

Figure S33. – Indomethacin-PLA solvent casted amorphous sample.

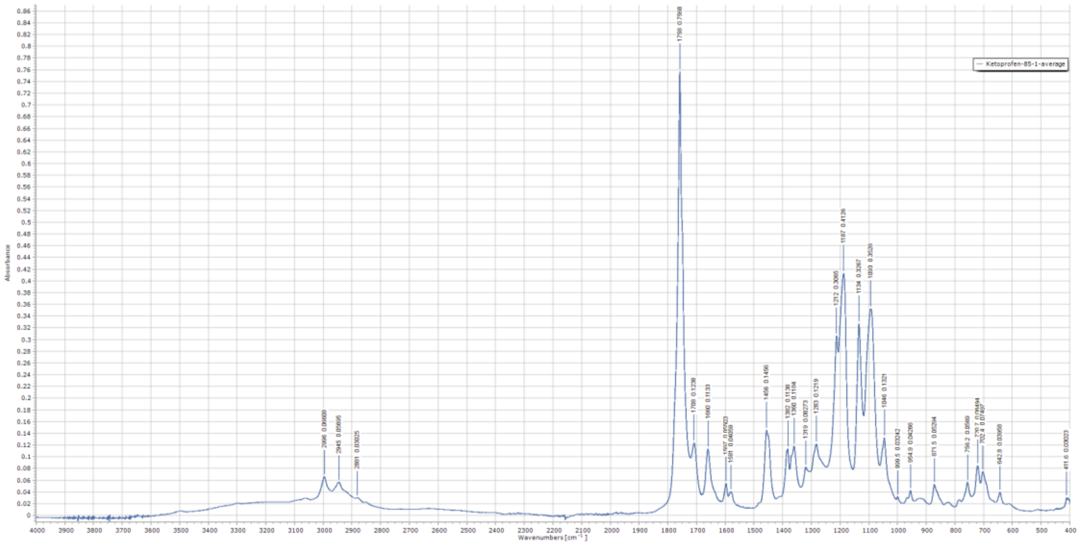

Figure S34. – Ketoprofen-PLA solvent casted amorphous sample.

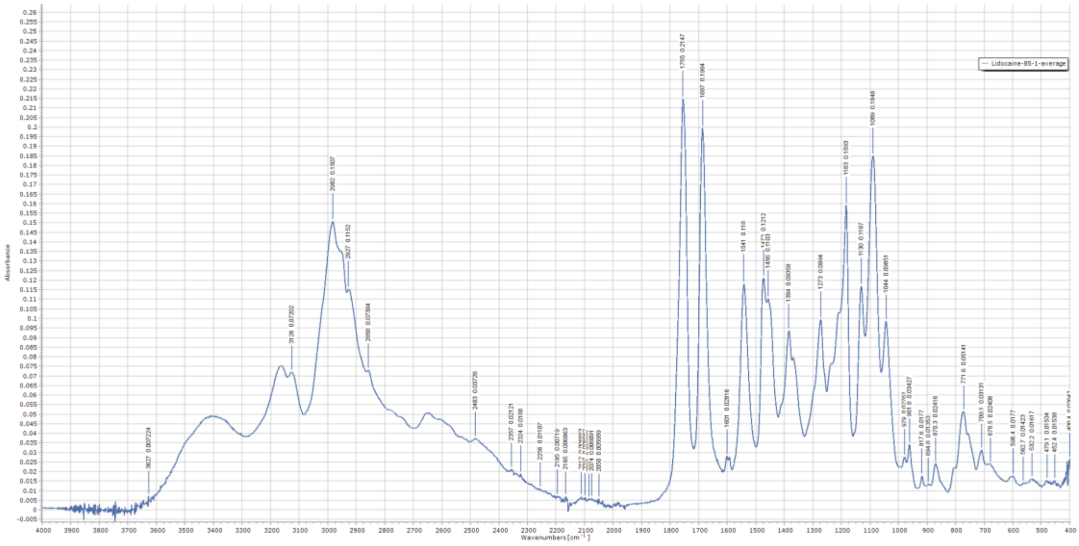

Figure S35. – Lidocaine-PLA solvent casted amorphous sample.

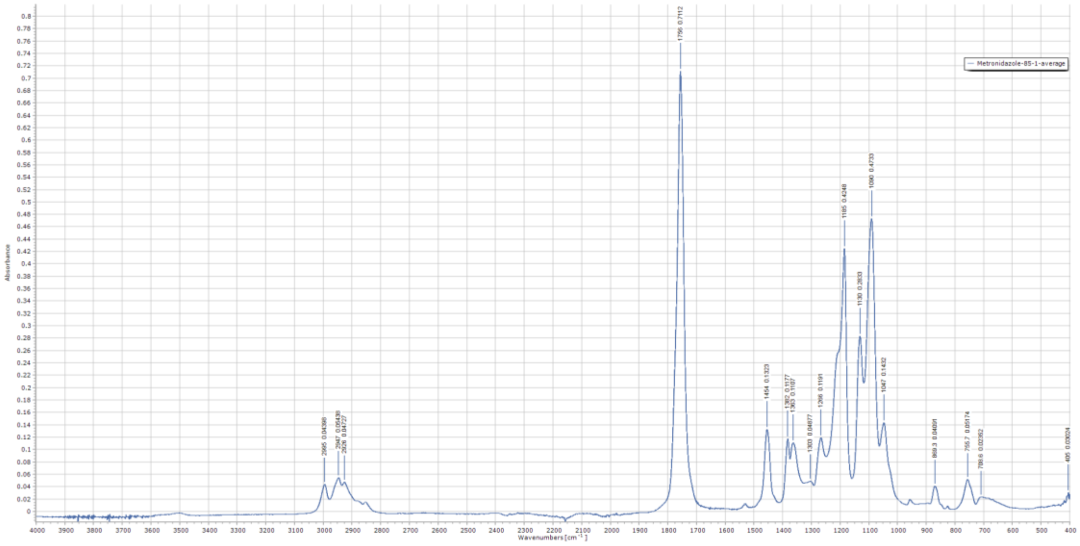

Figure S36. – Metronidazole-PLA solvent casted amorphous sample.

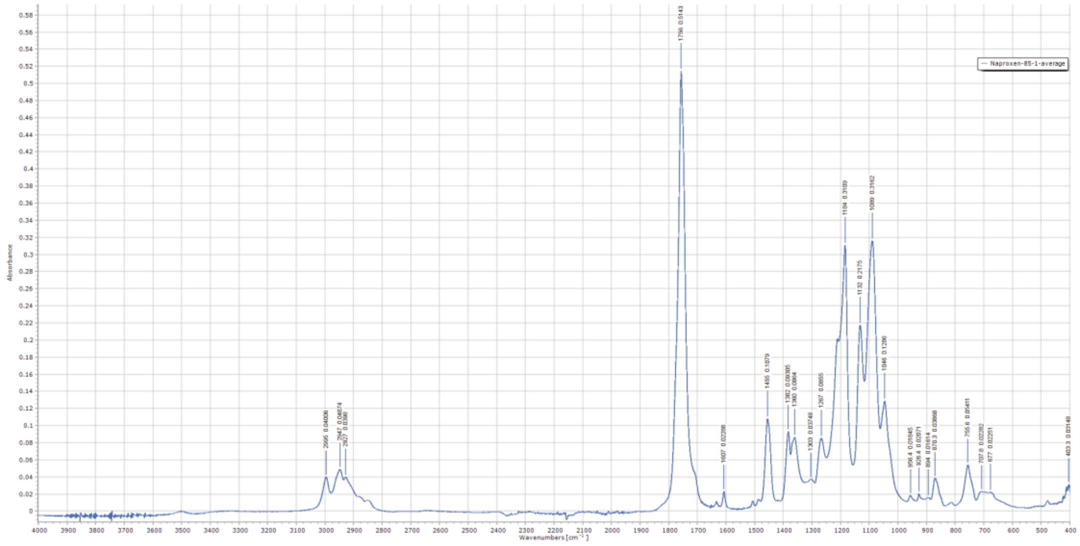

Figure S37. – Naproxen-PLA solvent casted amorphous sample.

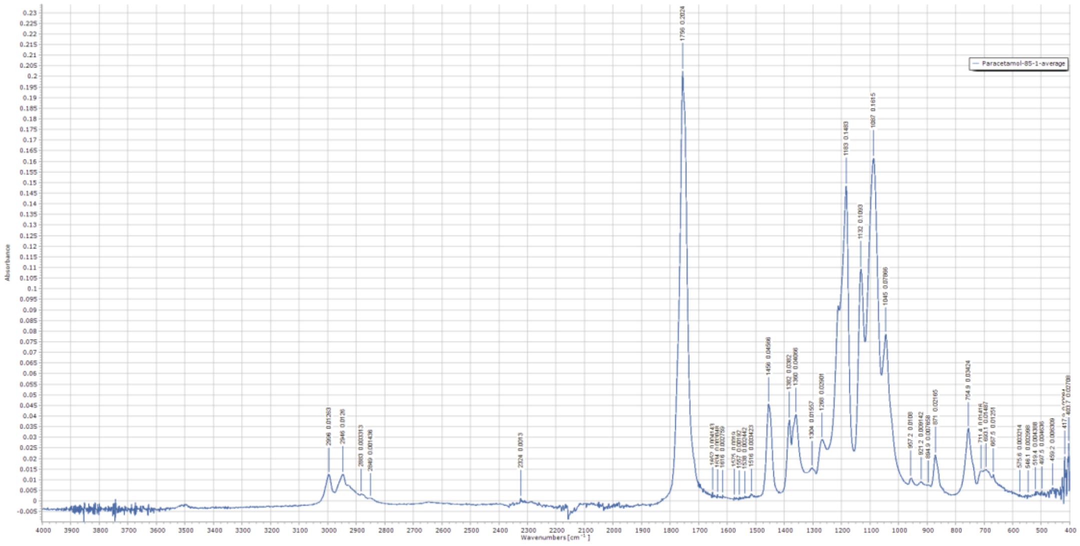

Figure S38. – Paracetamol-PLA solvent casted amorphous sample.

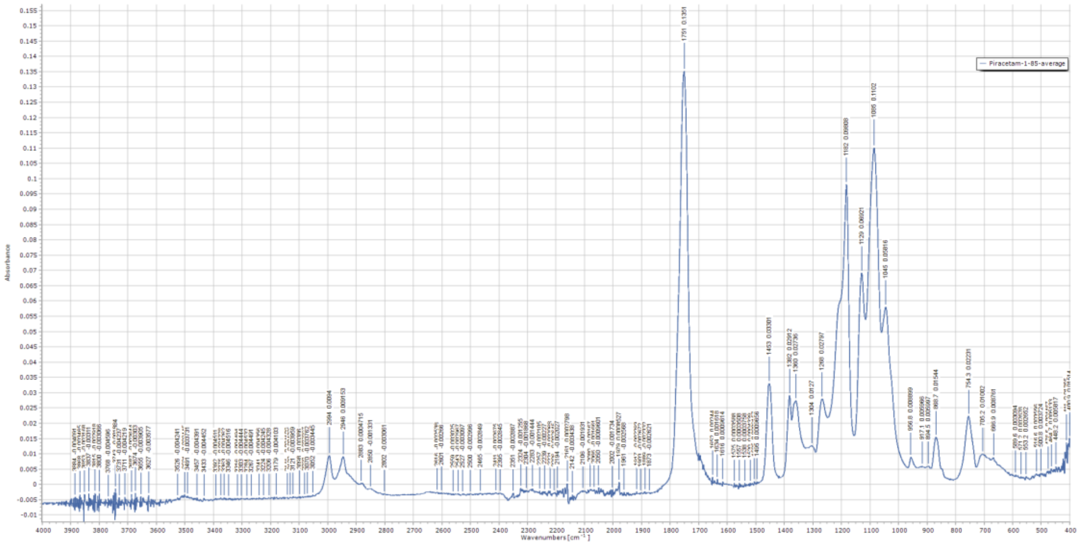

Figure S39. – Piracetam-PLA solvent casted amorphous sample.

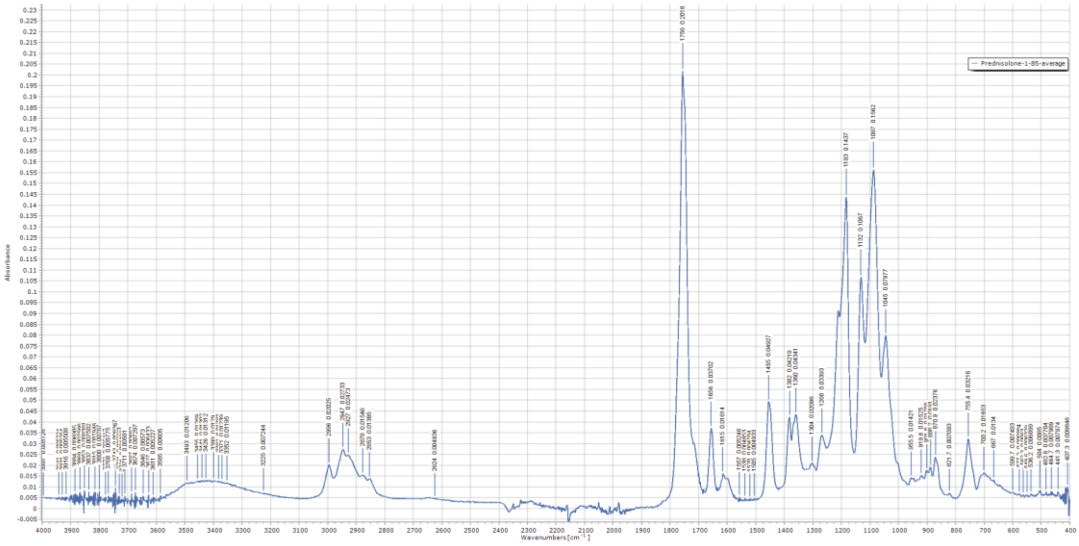

Figure S40. – Prednisolone-PLA solvent casted amorphous sample.

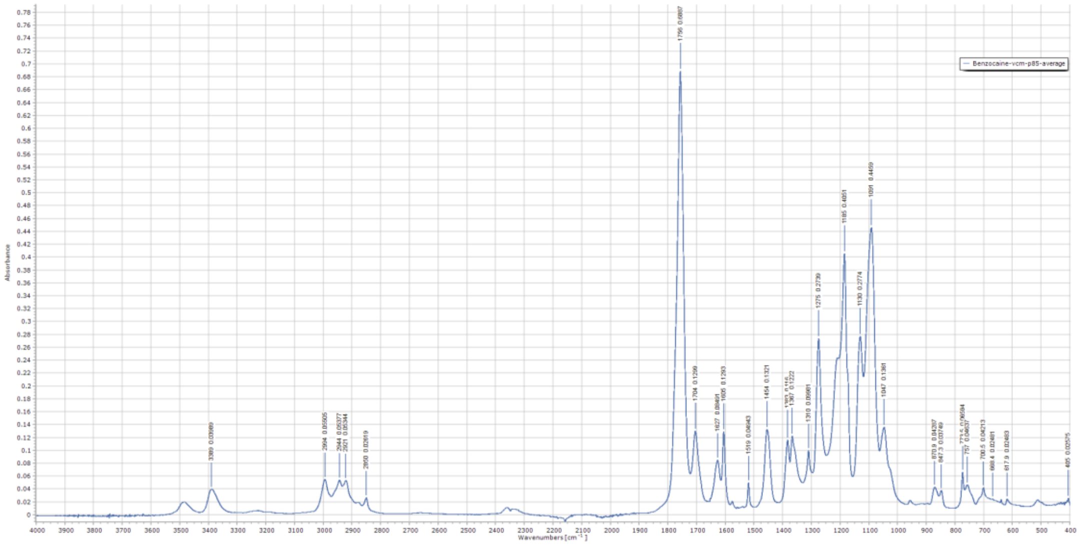

Figure S41. – Benzocaine-PLA VCM amorphous sample.

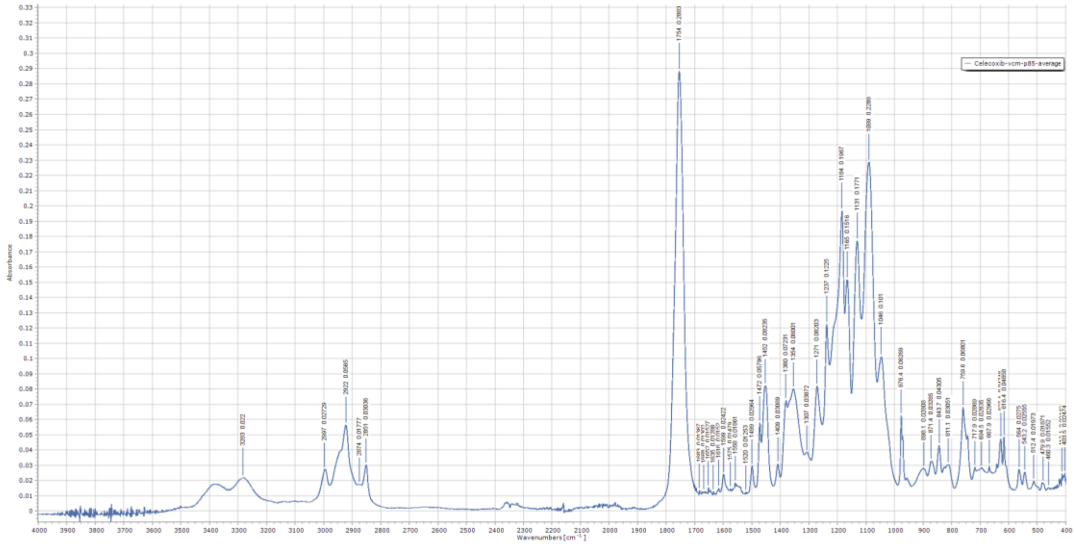

Figure S42. – Celecoxib-PLA VCM amorphous sample.

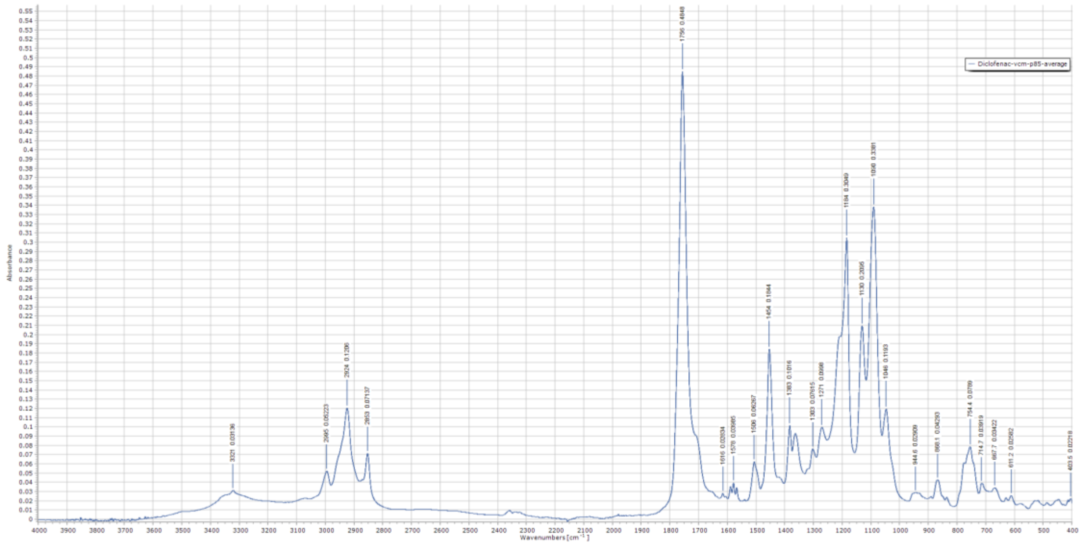

Figure S43. – Diclofenac-PLA VCM amorphous sample.

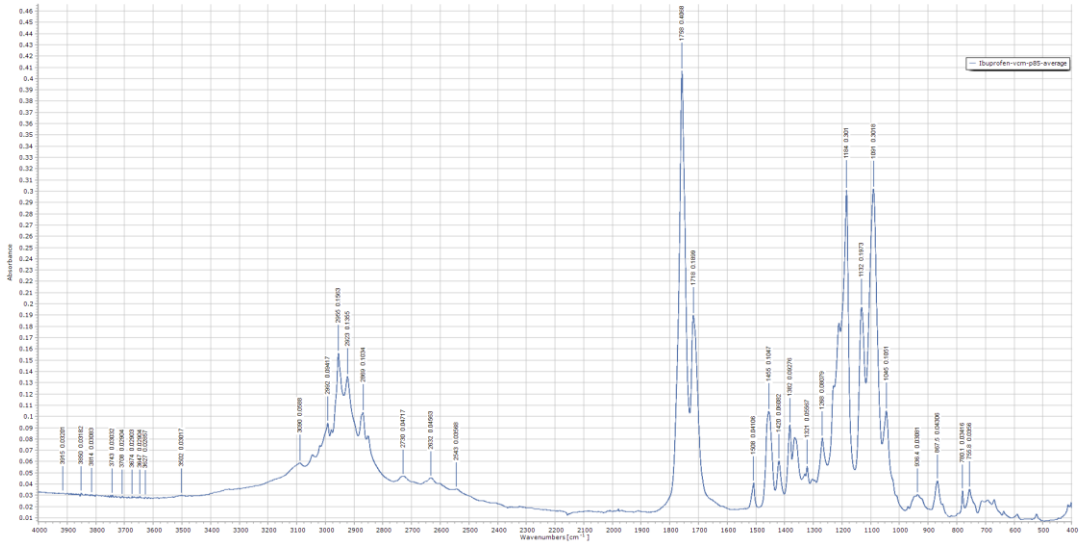

Figure S44. – Ibuprofen-PLA VCM amorphous sample.

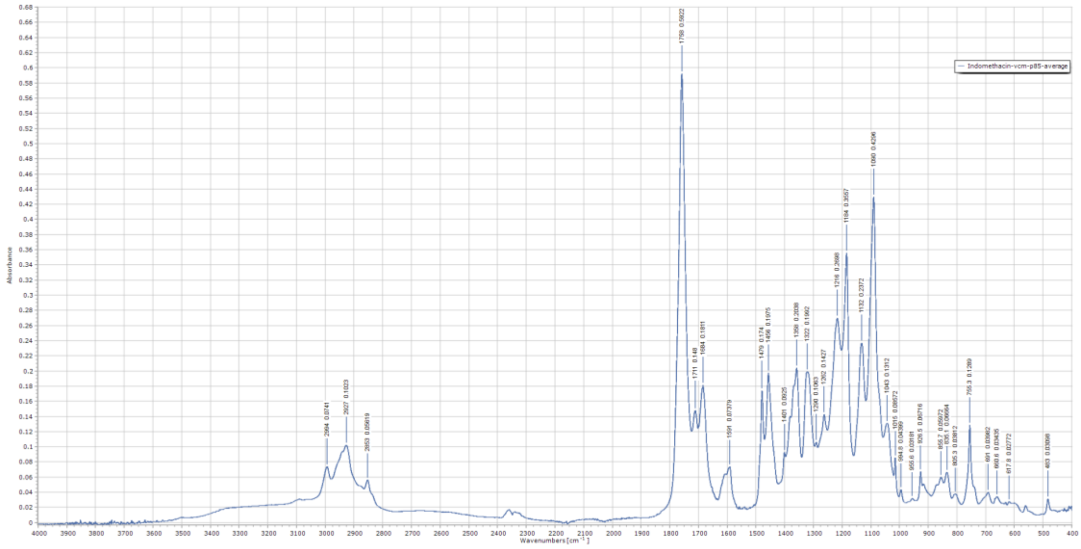

Figure S45. – Indomethacin-PLA VCM amorphous sample.

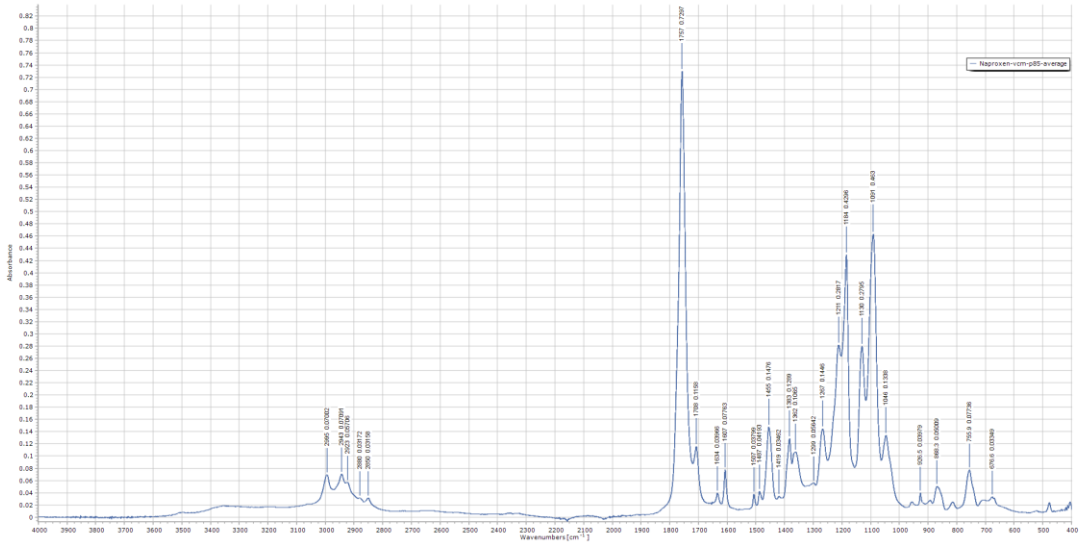

Figure S46. – Ketoprofen-PLA VCM amorphous sample.

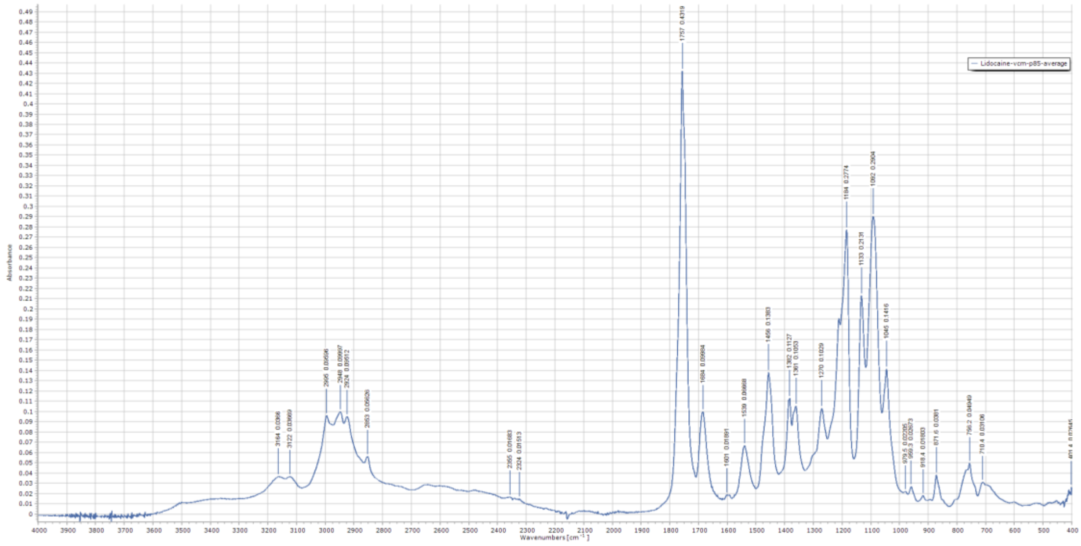

Figure S47. – Lidocaine-PLA VCM amorphous sample.

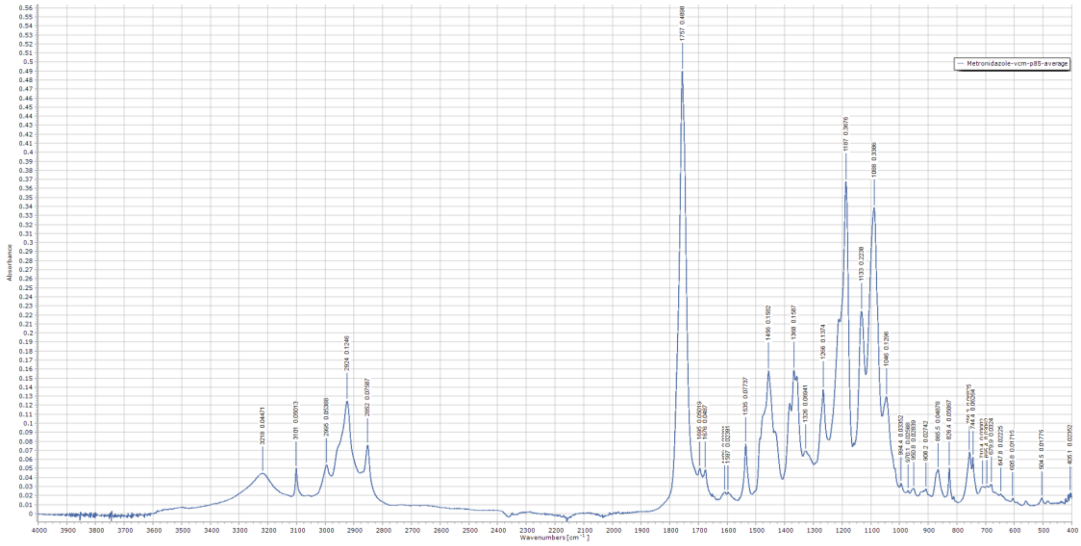

Figure S48. – Metronidazole-PLA VCM amorphous sample.

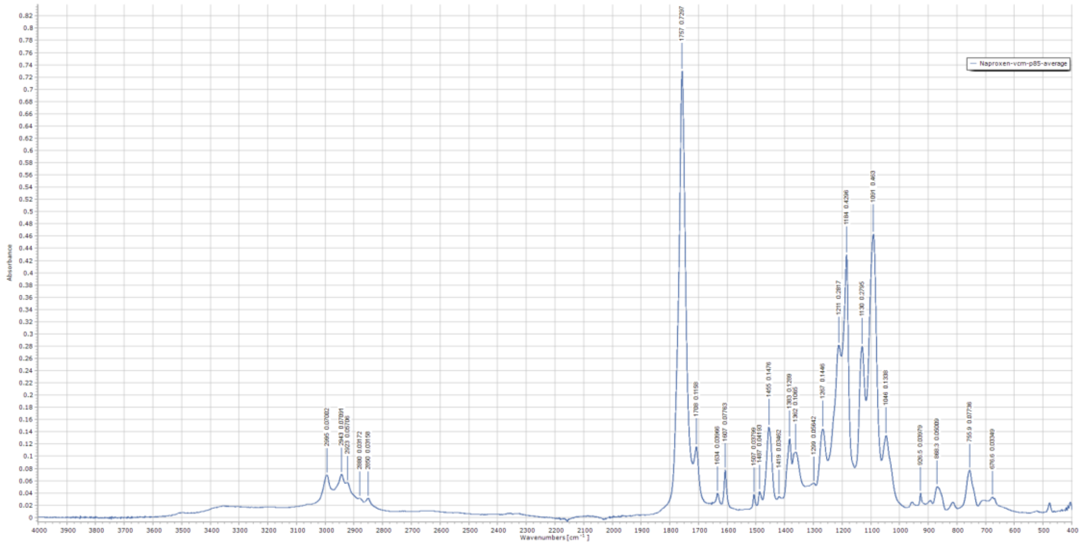

Figure S49. – Naproxen-PLA VCM amorphous sample.

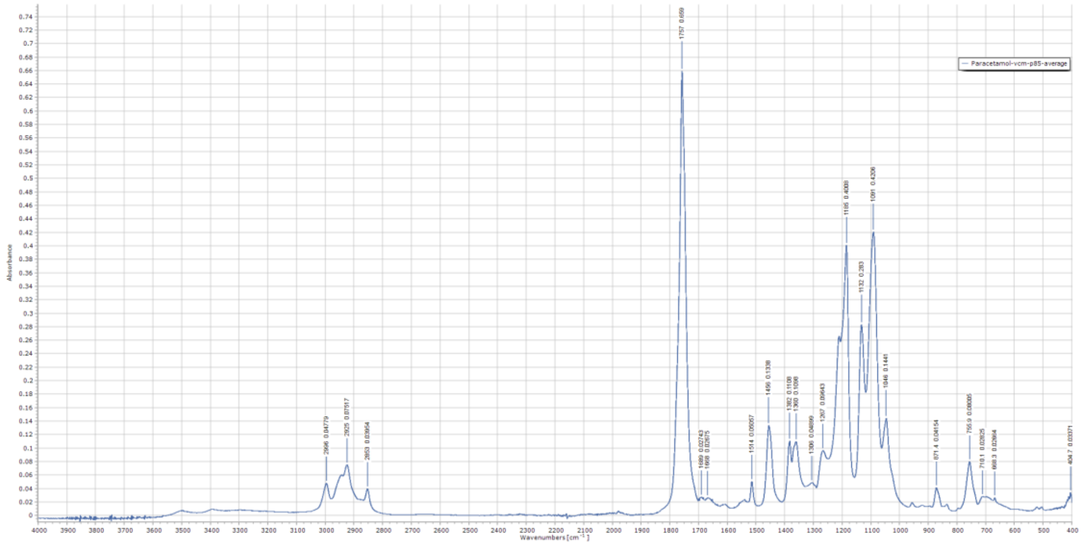

Figure S50. – Paracetamol-PLA VCM amorphous sample.

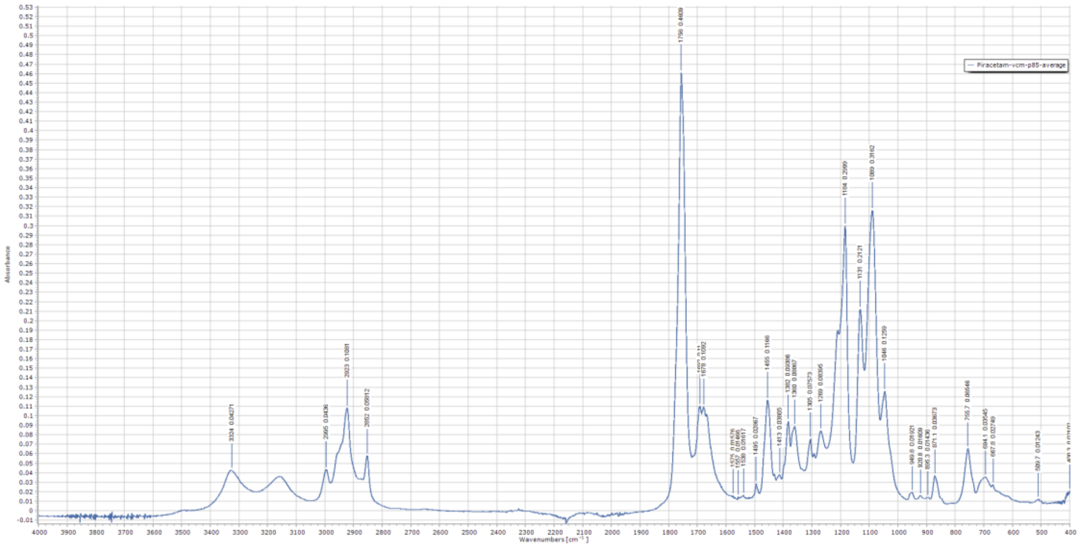

Figure S51. – Piracetam-PLA VCM amorphous sample.

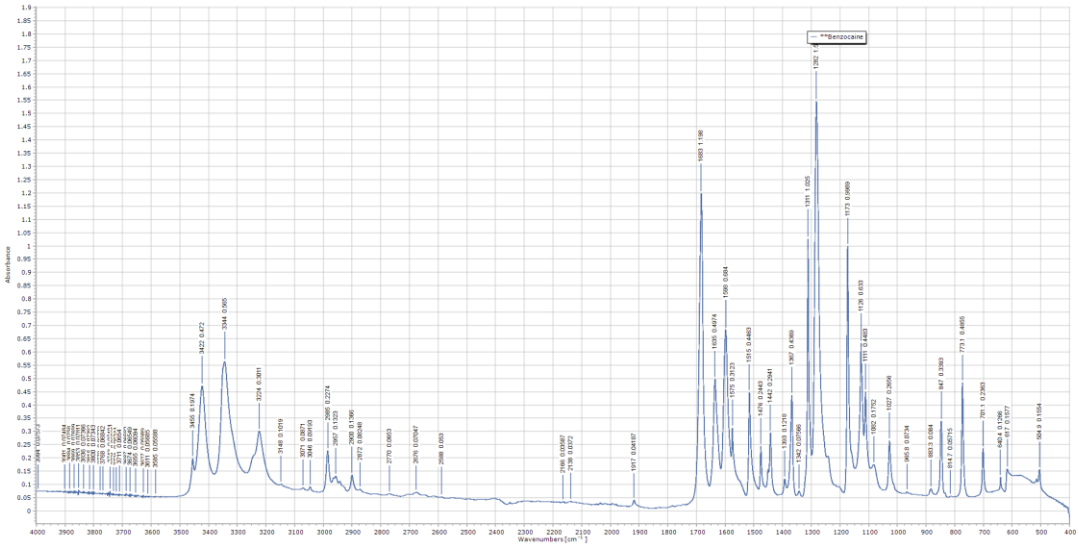

Figure S52. – Benzocaine.

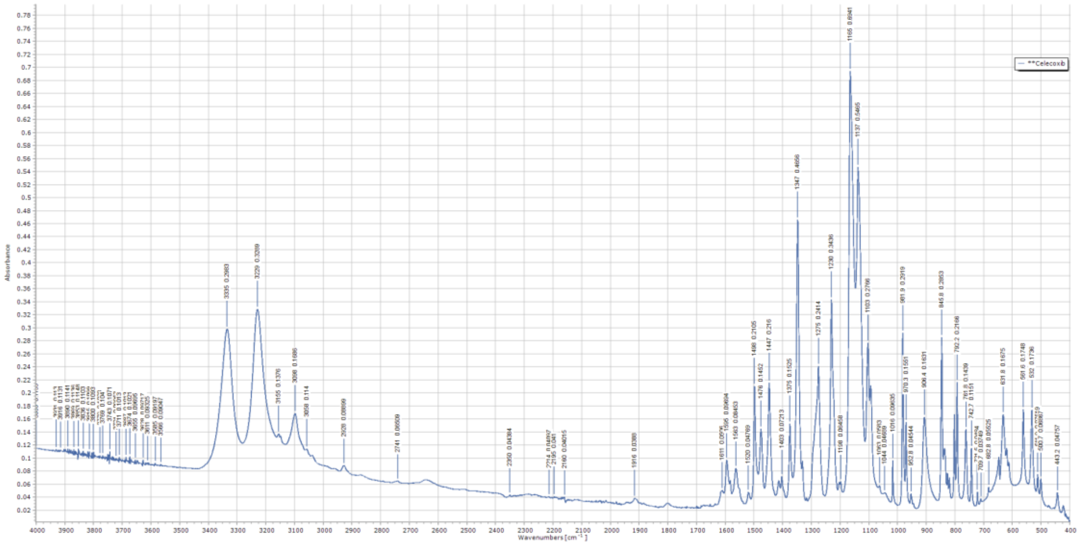

Figure S53. – Celecoxib.

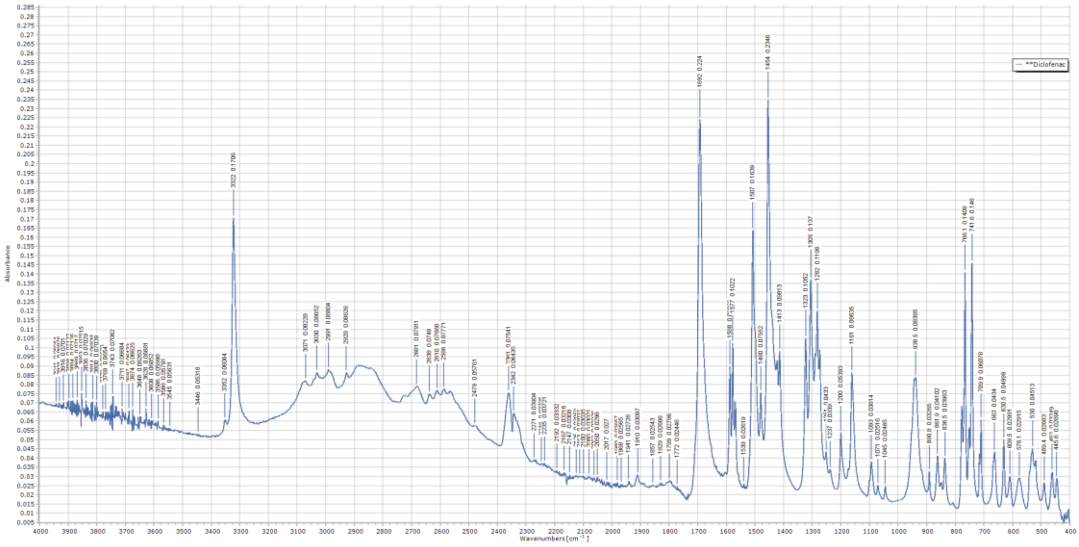

Figure S54. – Diclofenac.

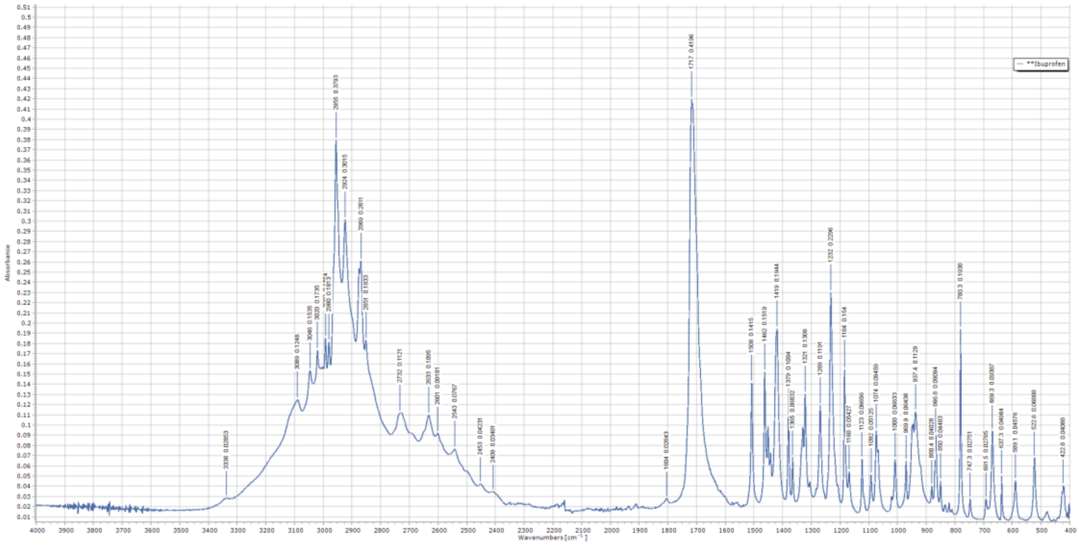

Figure S55. – Ibuprofen.

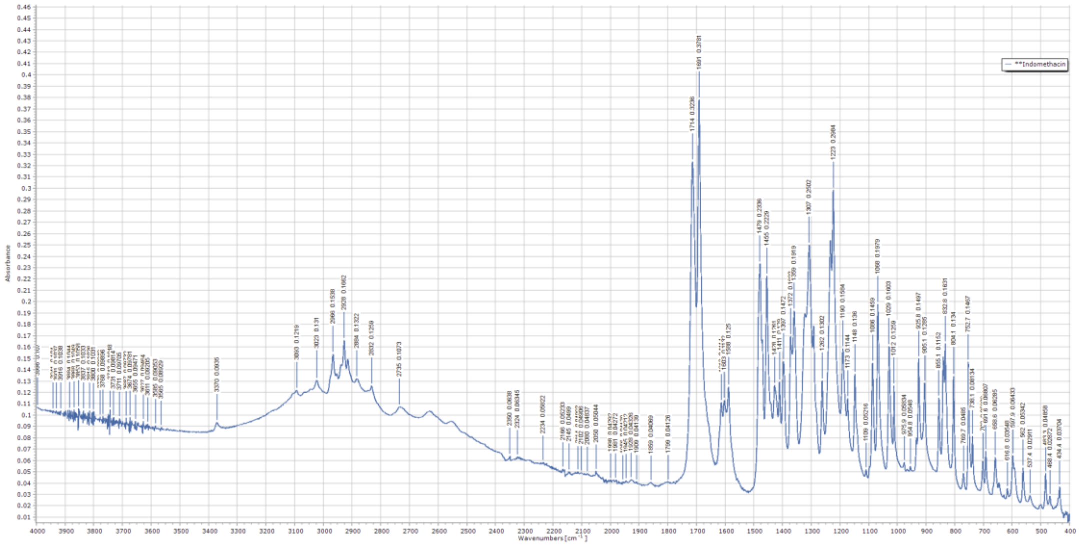

Figure S56. – Indomethacin.

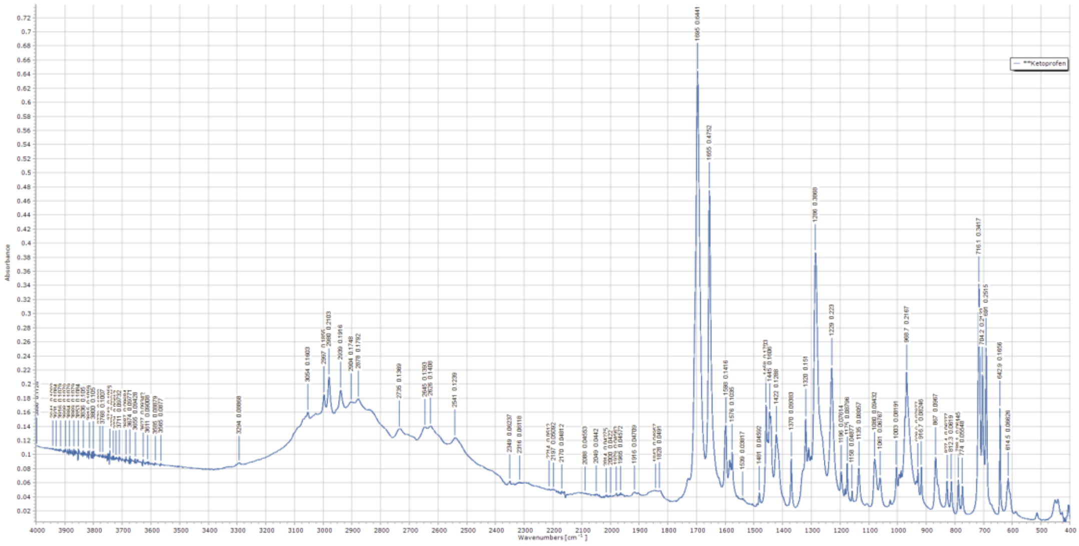

Figure S57. – Ketoprofen.

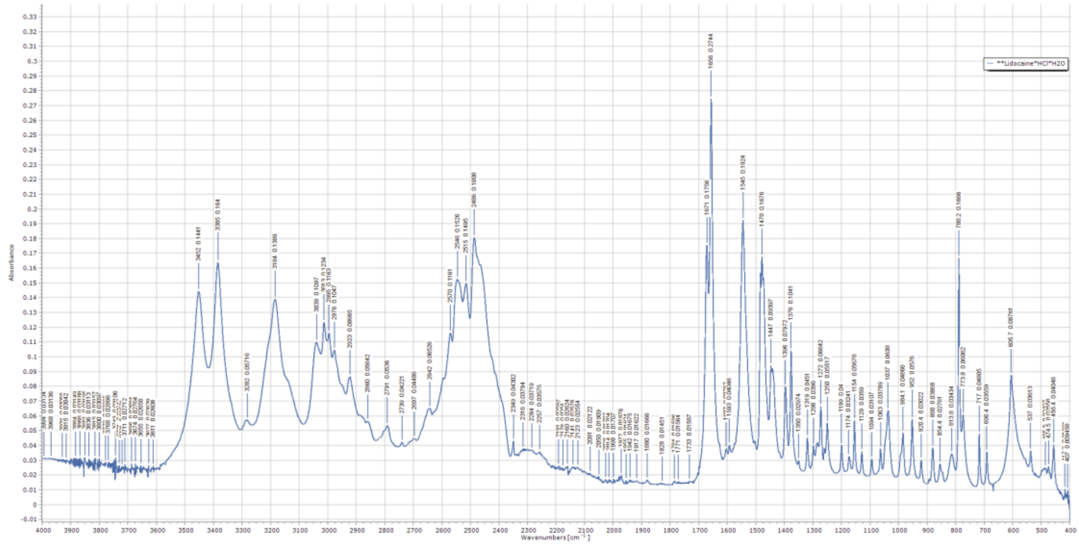

Figure S58. – Lidocaine*HCl*H2O.

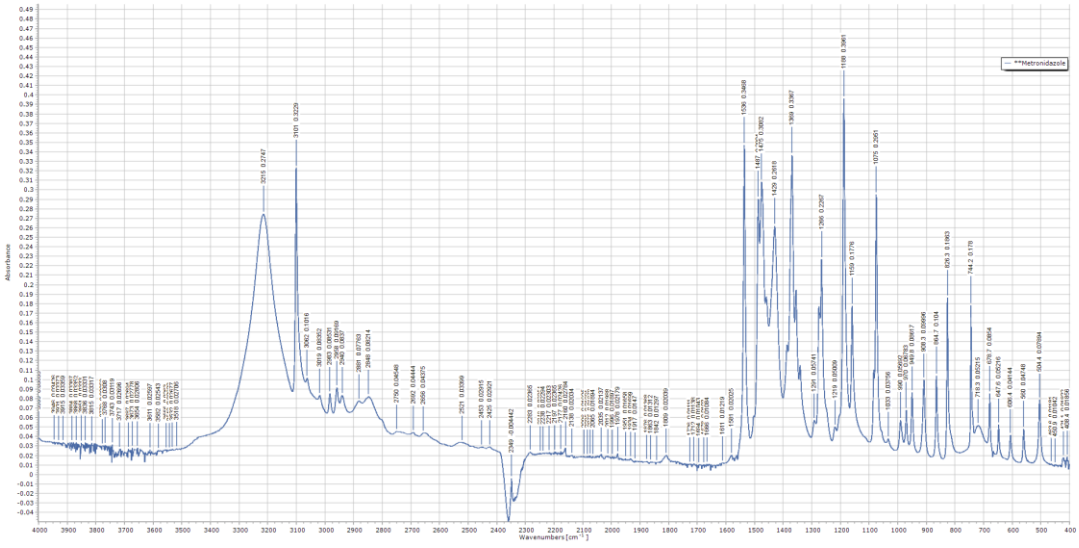

Figure S59. – Metronidazole.

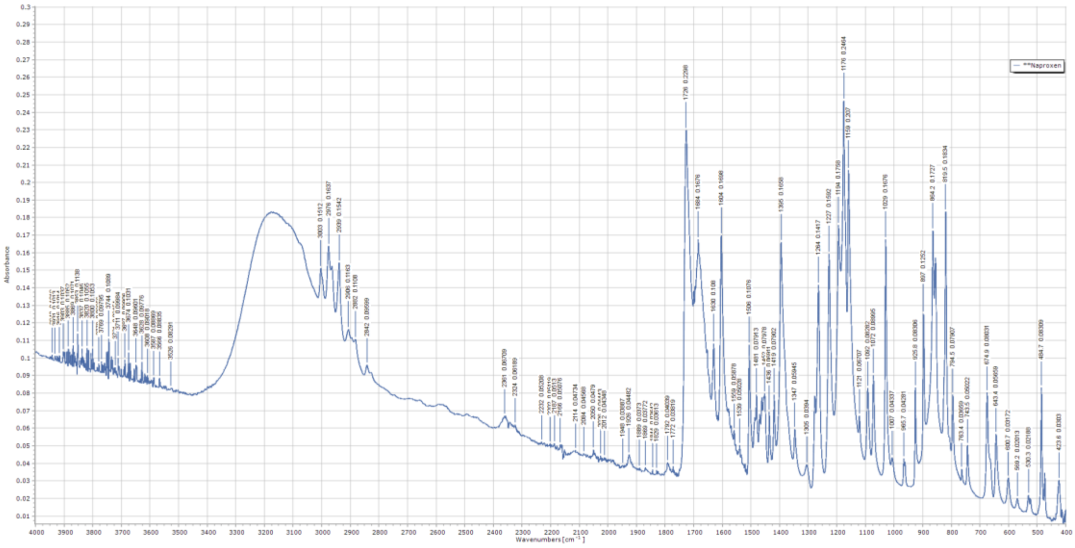

Figure S60. – Naproxen.

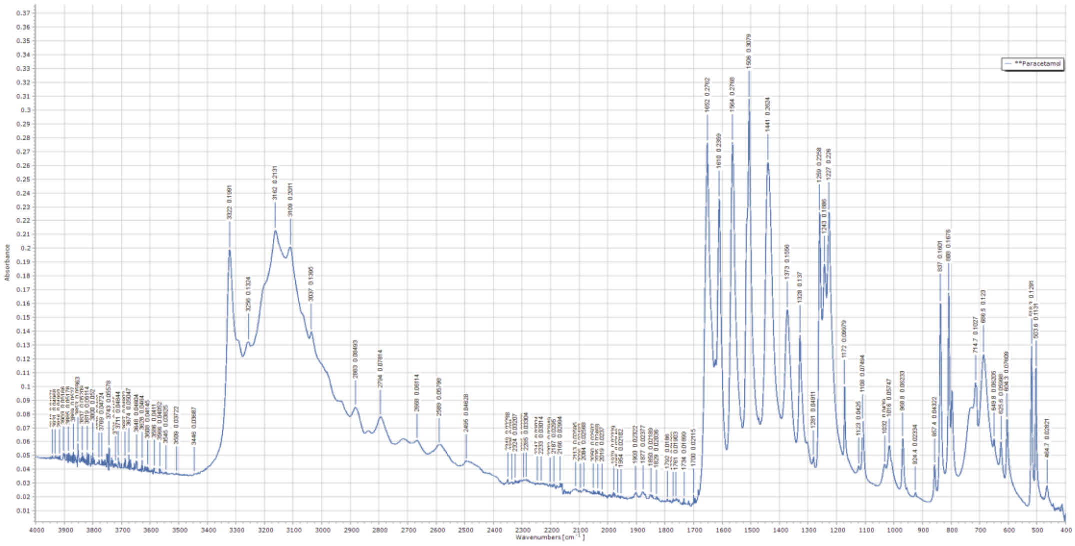

Figure S61. – Paracetamol.

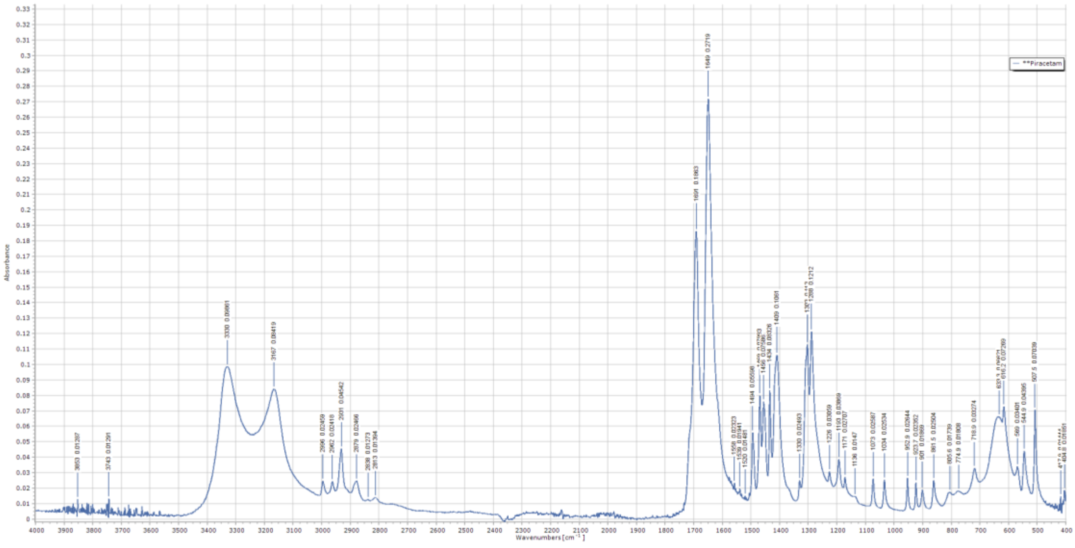

Figure S62. – Piracetam.

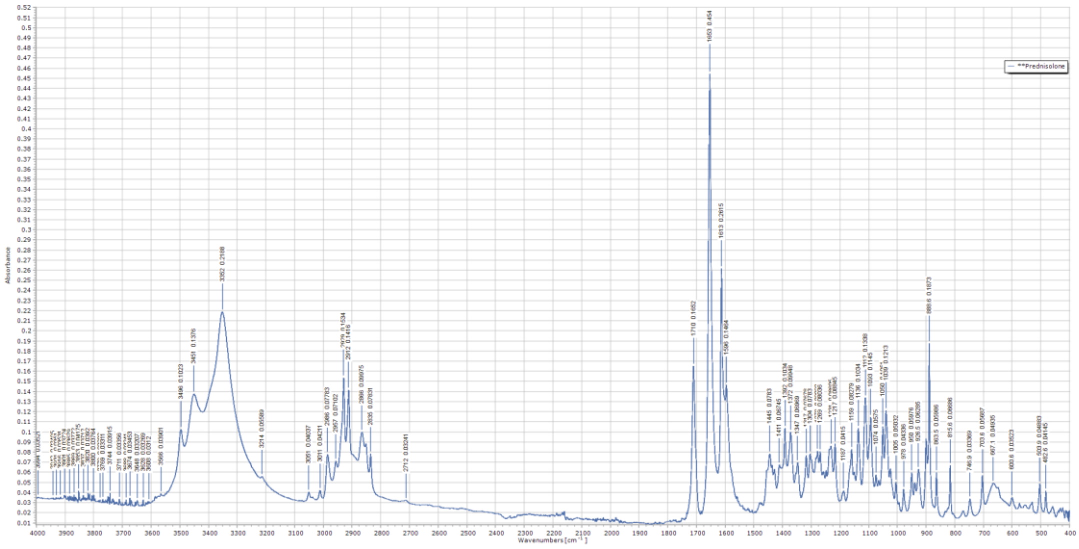

Figure S63. – Prednisolone.

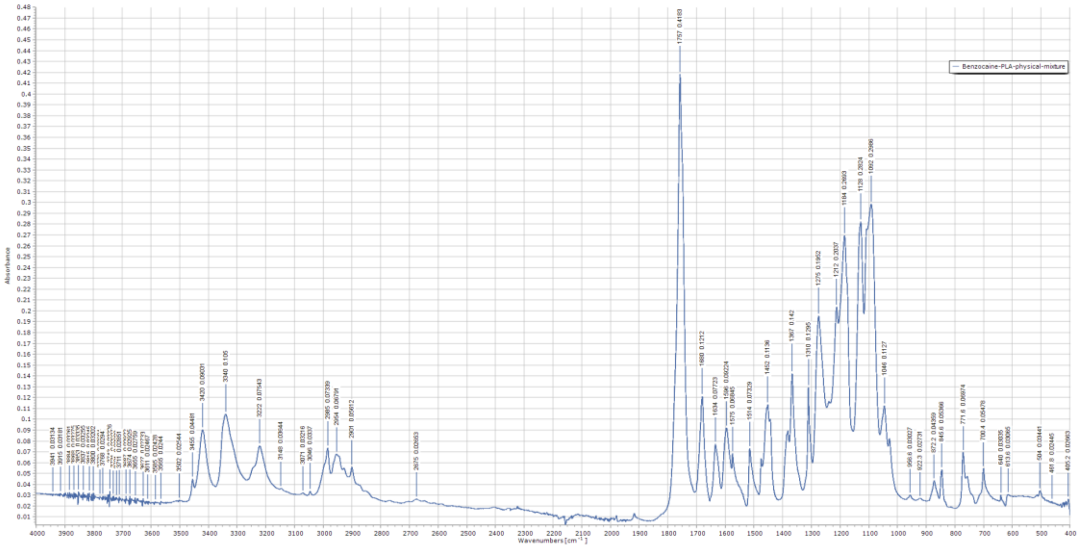

Figure S64. – Benzocaine-PLA physical mixture.

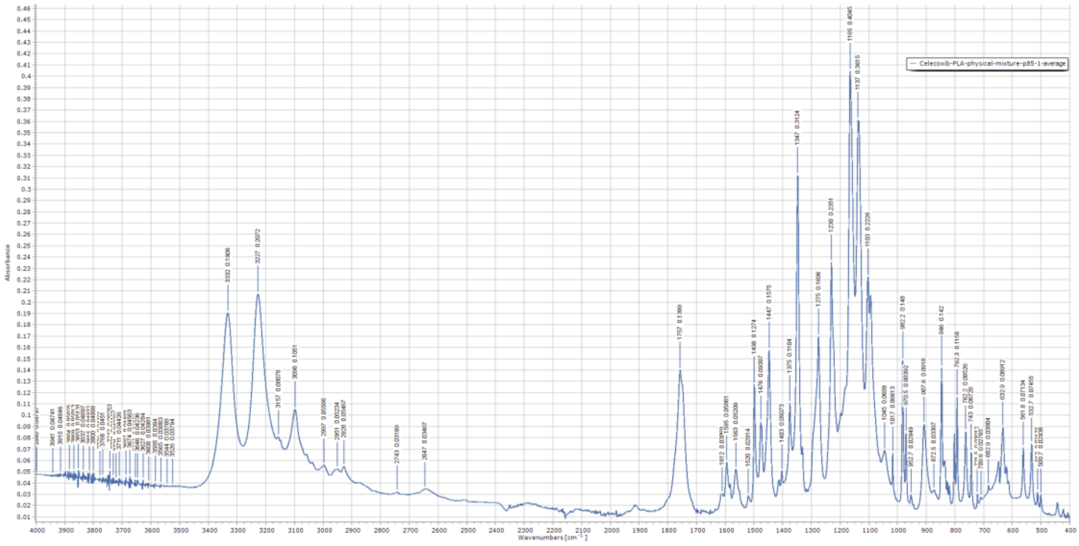

Figure S65. – Celecoxib-PLA physical mixture.

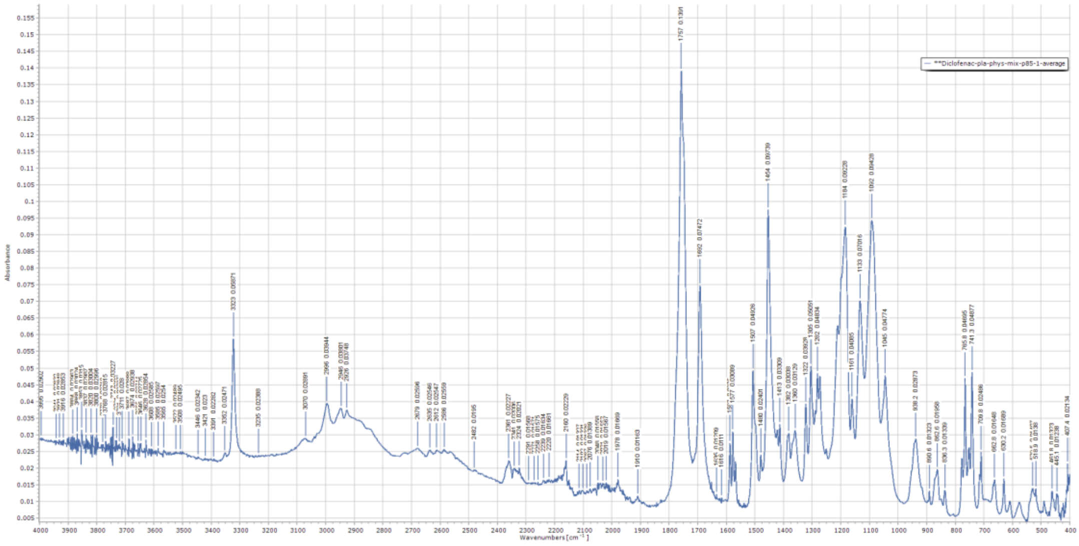

Figure S66. – Diclofenac-PLA physical mixture.

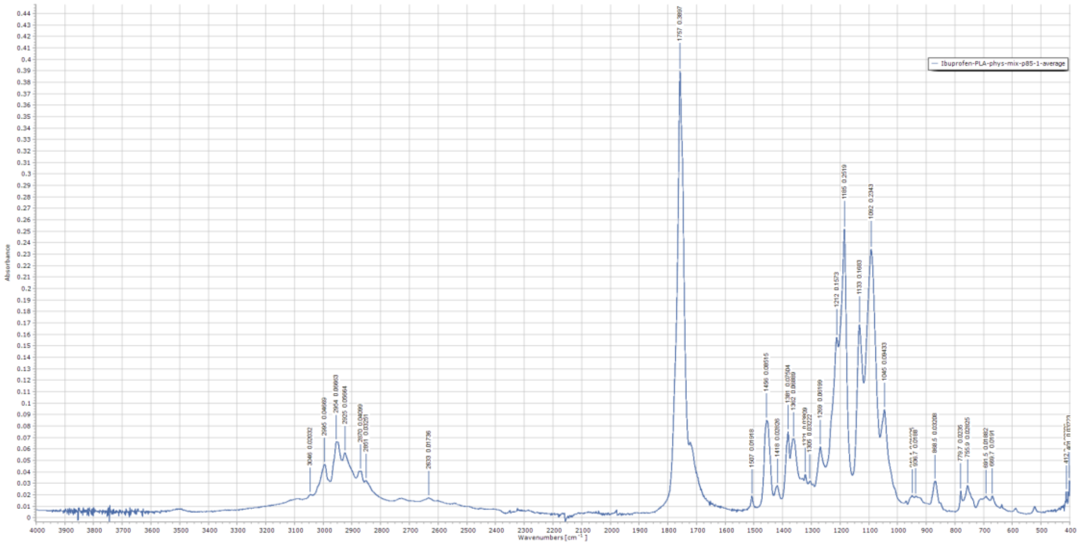

Figure S67. – Ibuprofen-PLA physical mixture.

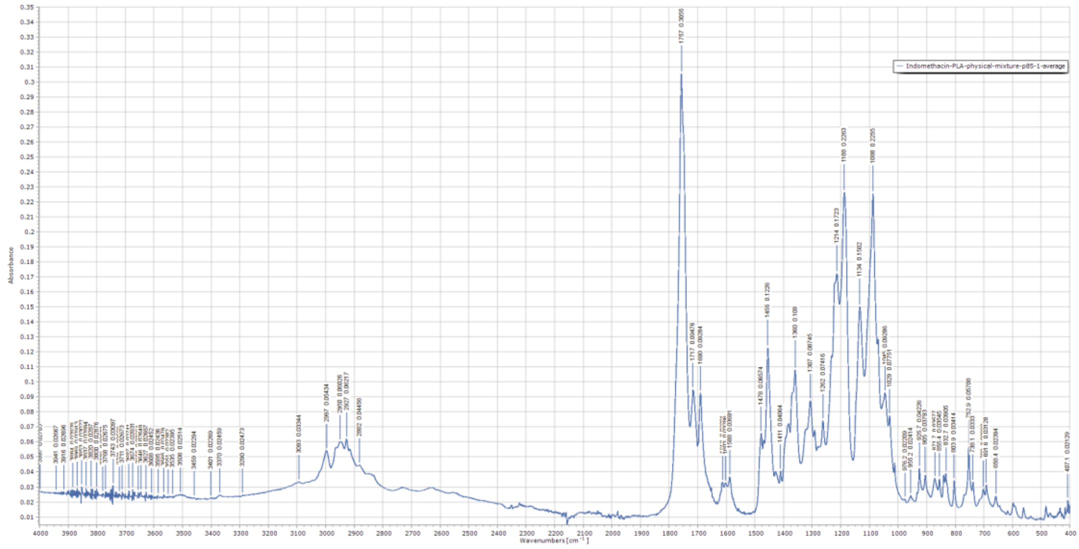

Figure S68. – Indomethacin-PLA physical mixture.

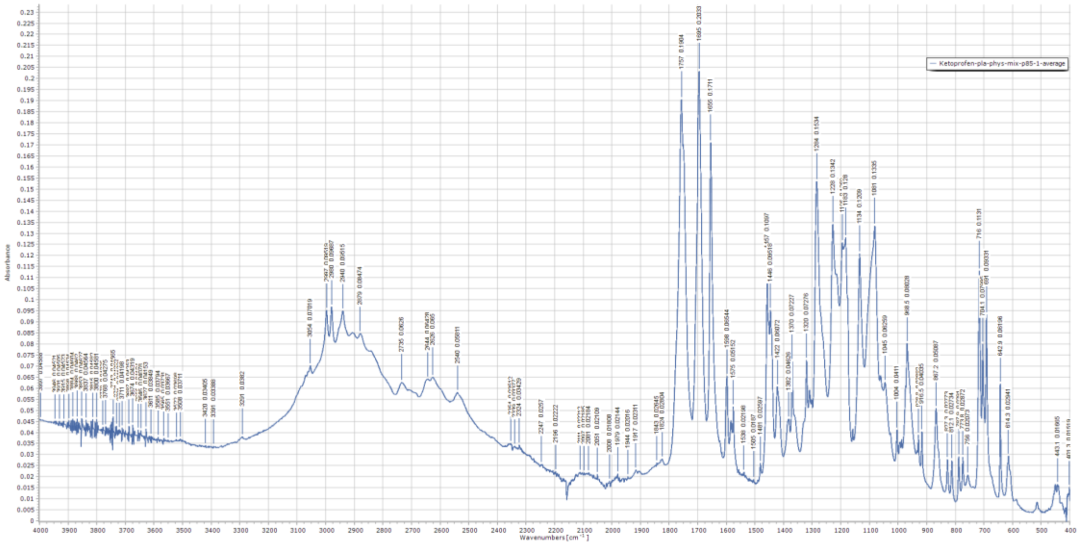

Figure S69. – Ketoprofen-PLA physical mixture.

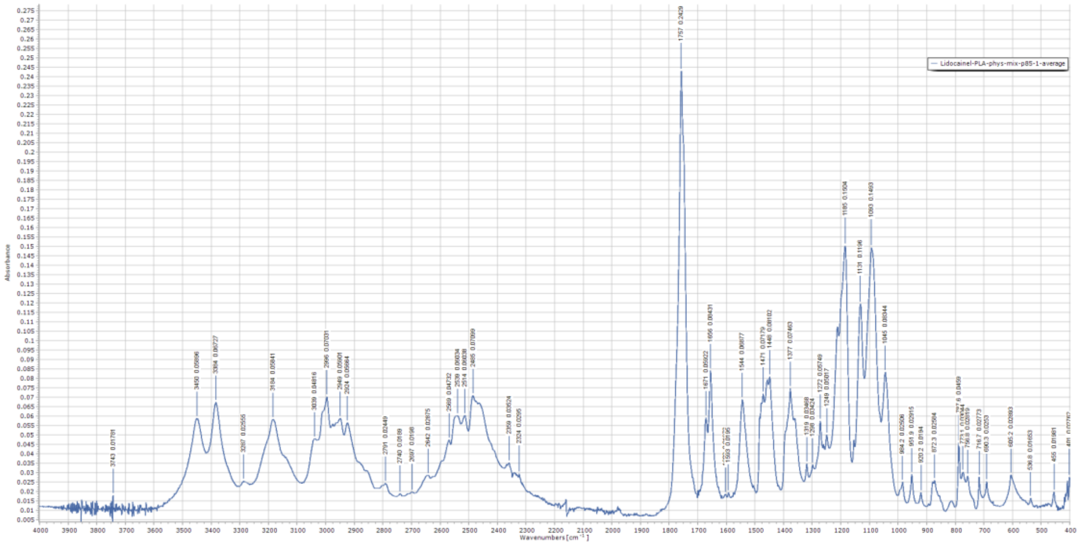

Figure S70. – Lidocaine-PLA physical mixture.

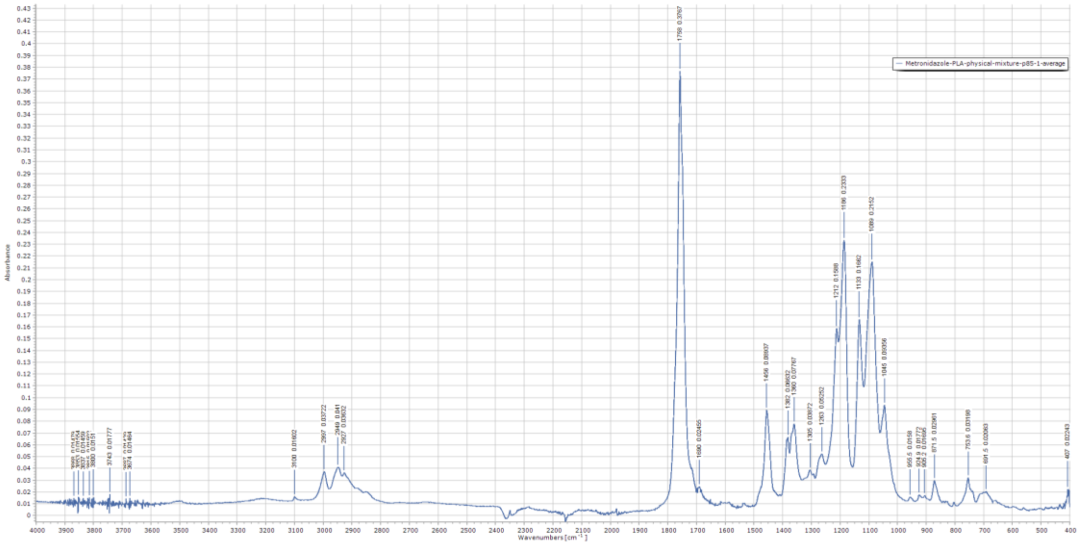

Figure S71. – Metronidazole-PLA physical mixture.

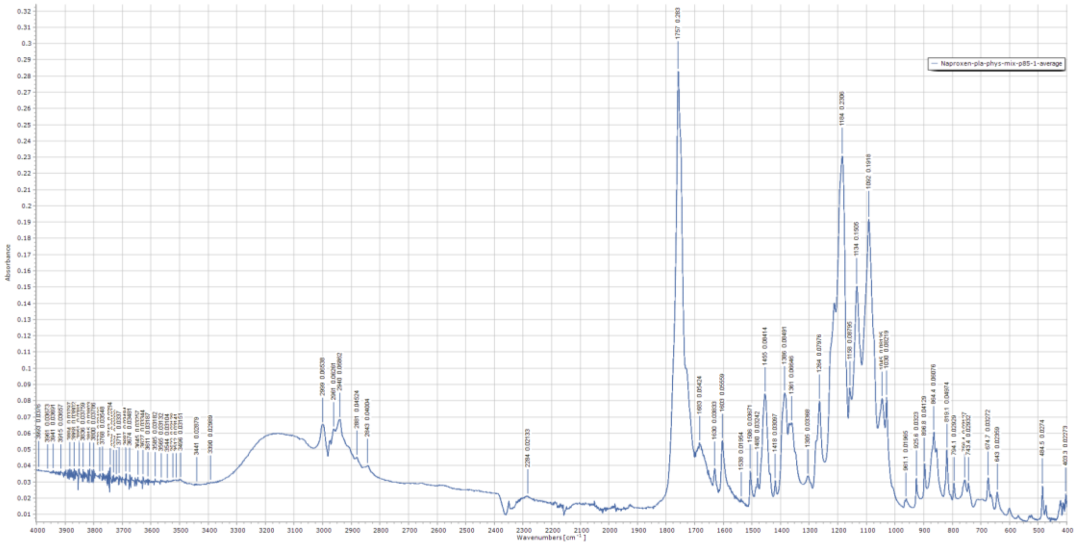

Figure S72. – Naproxen-PLA physical mixture.

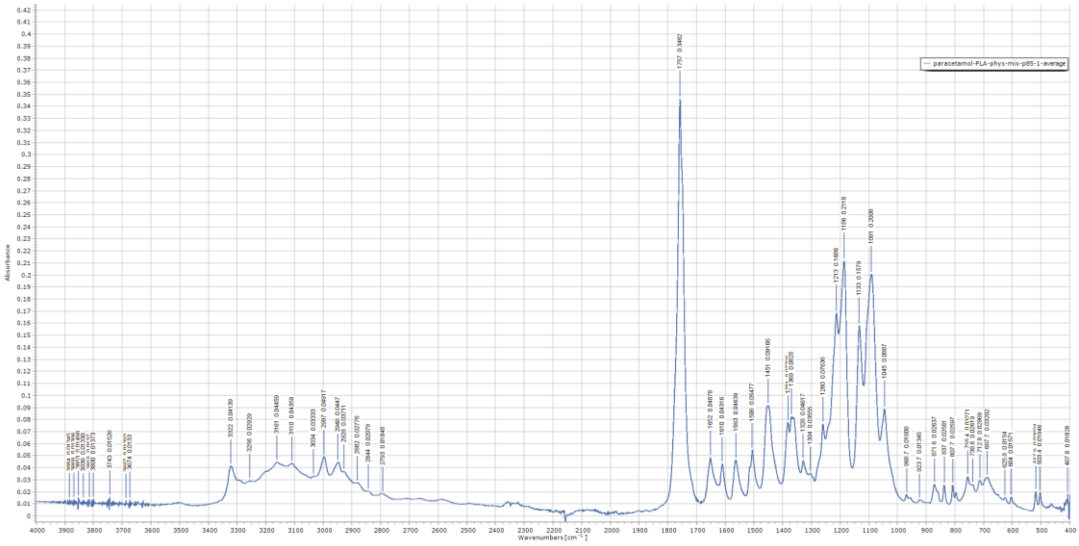

Figure S73. – Paracetamol-PLA physical mixture.

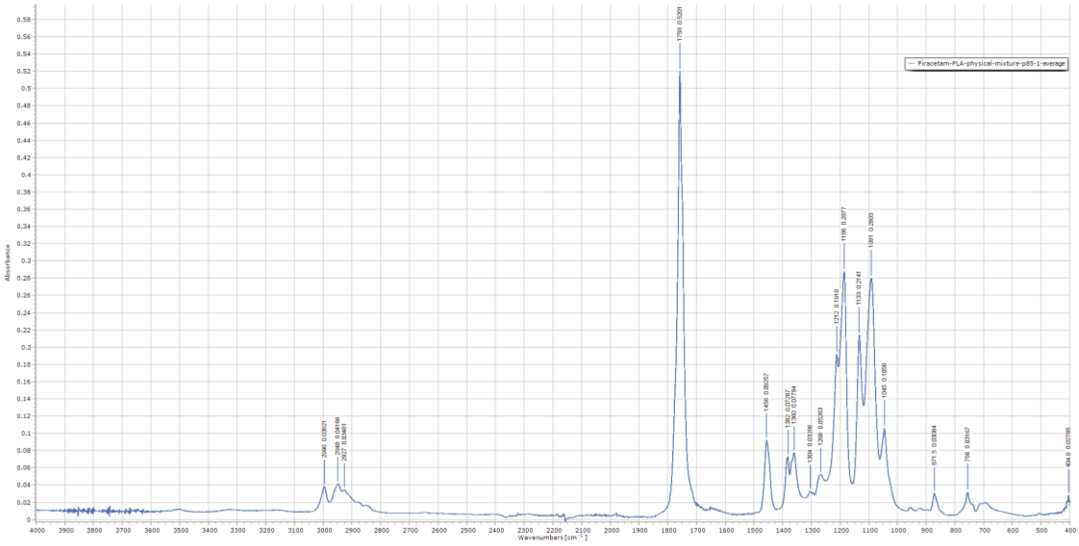

Figure S74. – Piracetam-PLA physical mixture.

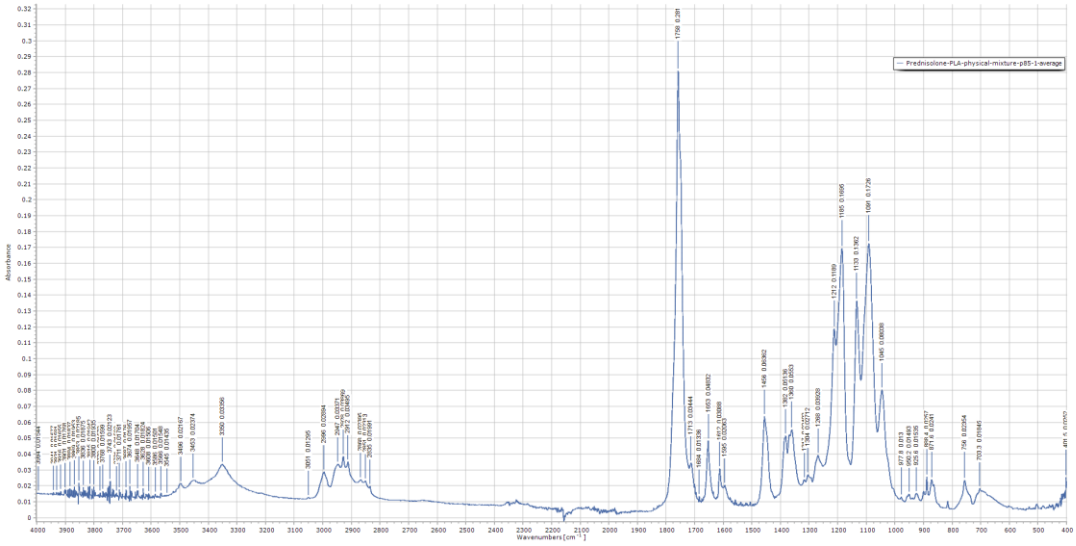

Figure S75. – Prednisolone-PLA physical mixture.
